# Embracing Phase Transitions to Rationalize the High Oxygen Evolution Reaction Activity of Nickel Selenides in Alkaline Electrolytes

**DOI:** 10.1021/jacs.6c09277

**Published:** 2026-11-02

**Authors:** Felix Hiege, Chun-Wai Chang, Oliver Trost, Pouya Hosseini, Sheila Hernandez, Carsten Dosche, Gabriel Boitel-Aullen, Raquel Aymerich-Armengol, Christoph J. Bondue, Andrea M. Mingers, Jan Clausmeyer, Alvin Chang, Qing Ma, Gunther Wittstock, Zhenxing Feng, Kristina Tschulik

**Affiliations:** † Faculty of Chemistry and Biochemistry, Chair of Analytical Chemistry, 9142 Ruhr University Bochum, Universitätsstrasse 150, 44801 Bochum, Germany; ‡ School of Chemical, Biological, and Environmental Engineering, 2694 Oregon State University, Corvallis, Oregon 97333, United States; § Max-Planck-Institut für Nachhaltige Materialien GmbH, Max-Planck-Straße 1, 40237 Düsseldorf, Germany; ∥ Institute of Chemistry, School of Mathematics and Science, Carl von Ossietzky University Oldenburg, Ammerländer Heerstrasse 114–118, 26129 Oldenburg, Germany; ⊥ DND-CAT, Synchrotron Research Center, Northwestern University, Evanston, Illinois 60208, United States

## Abstract

The transformation of electrodeposited NiSe nanocatalysts was studied under oxygen evolution reaction (OER) conditions in alkaline electrolytes by a combination of *operando* X-ray absorption (XAS) and *operando* electrochemical impedance spectroscopy (EIS). The upper layers of nickel selenides transform into nickel oxide/oxyhydroxide species, which exhibit higher catalytic OER activity than the as-synthesized nickel oxide/oxyhydroxide, as reported previously. The coupled *operando* XAS and EIS experiments reveal that the Ni^3+^/Ni^4+^ transition occurs at lower potentials than the OER, involving a transient phase transformation at the foot of the nickel oxidation peak. Based on these findings and using insights from complementary *operando* (Raman spectroscopy, scanning flow cell inductively coupled plasma mass spectrometry) and *ex situ* measurements (XAS and X-ray photoelectron spectroscopy), a catalytic cycle is proposed within the framework of frustrated phase transitions linking the molecular transformation of substrate and products (H_2_O/O_2_) to structural and electronic changes of the solid catalyst (β-Ni­(OH)_2_/β-NiOOH). Specifically, the Ni–O distance of the formed β-NiOOH surface species on top of the NiSe layers elongates, facilitating rapid local transitions between different catalyst phases and oxidation states, which are essential for rapid completion of the catalytic cycle under *operando* conditions.

## Graphical abstract

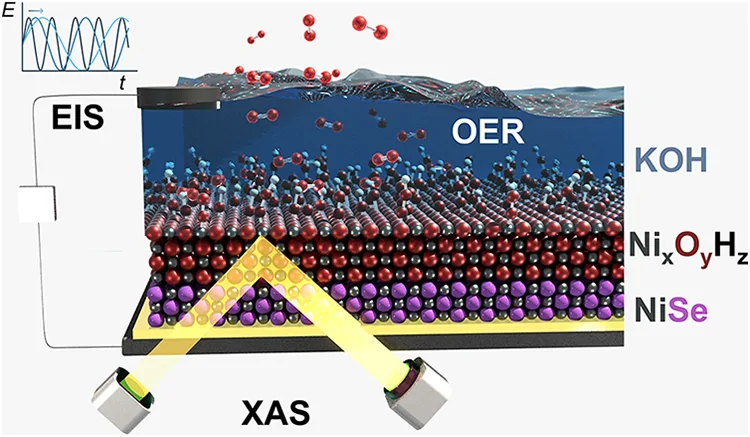

## 1 Introduction

In recent years, nickel selenides have been studied extensively as a potentially promising replacement for precious metal electrocatalysts in water-splitting applications.^1–4^ However, nickel selenides were found to be precatalysts under oxygen evolution reaction (OER) conditions in alkaline electrolytes. When the applied potential is positive (anodic) enough to drive the OER, they are transformed into nickel oxide species, e.g., (oxy)­hydroxides.^4,5^ Interestingly, nickel (oxy)­hydroxide generated from nickel selenide precatalysts showed superior catalytic properties to as-synthesized nickel (oxy)­hydroxides.^5,6^ The reasons for this finding of improved catalytic activity are still under debate, even though several hypotheses based on experimental and theoretical approaches have been made in the literature. For example, Zhong et al.^7^ reported a downshift of the d-band based on combined results from chronoamperometric experiments and density functional theory (DFT) calculations. This shift is proposed to facilitate the deprotonation of OH* intermediates in the OER-catalytic cycle relative to as-synthesized nickel hydroxides.^7^ Furthermore, they concluded that the e_g_* band of nickel selenide-based electrocatalysts broadens close to the Fermi level, which, as they suggest, promotes electron transfer mobility and, hence, OER performance.^7^

Furthermore, the addition of a selenium precursor to an alkaline electrolyte solution during OER testing of as-synthesized nickel oxyhydroxide can improve the OER-catalytic activity, according to Shi et al.^8^ Further studies reported the adsorption and intercalation of selenate ions, which were generated during the transformation of nickel selenides to (oxy)­hydroxides, as well as other (oxy)­anions using X-ray photoelectron (XPS) and Raman spectroscopies. They concluded that stabilization of the OOH* compared to the OH* intermediate is responsible for the enhanced OER activity in electrochemical experiments, either when adding selenium to the alkaline electrolyte or when generating the active species from nickel selenide precatalyst materials.^8–10^

*Operando* X-ray absorption spectroscopy (XAS) studies were frequently conducted to investigate the active site of nickel oxide-based OER electrocatalysts under applied potentials.^3,11–16^ Recent XAS studies of the groups of Dau and Strasser indicated that the presence of the Ni^4+^ states is crucial for a high OER activity of nickel oxide/oxyhydroxide-based catalysts.^17–19^ For example, Drevon et al. found stable OOH* intermediates when looking on Ni, Fe, and O K-edges using soft and hard X-ray XAS.^17^ They reported that the concentration of Ni^4+^ states, which is necessary for O–O coupling, could be decreased by a significant iron content in the catalyst. However, they concluded that the metal oxidation state changes from Ni^2+^ to Ni^3+^/Ni^4+^ already at pre-OER potentials (<1.4 V_RHE_).^17^

Electrochemical impedance spectroscopy (EIS) is a powerful tool for probing the electrochemical interface under potential perturbation.^20,21^ Different OER intermediates form at the interface of the electrocatalyst and the electrolyte.^22–24^ Hence, we herein utilize changes in EIS at selected potentials of interest to improve our understanding of the chemical transformations of the catalyst–electrolyte interphase during the catalytic process. Notably, EIS has rarely been applied to relate changes in the oxidation states of catalytic metal centers (known from spectroscopy) to different charge transfer or mass transport processes under catalytic conditions.

In this study, we combined *operando* XAS in fluorescence mode at the Ni K-edge with EIS and chronoamperometric methods to study the reaction-induced changes in the chemical identity, e.g., oxidation states and bond lengths, of electrodeposited nickel selenide at potentials in pre-OER and OER ranges in alkaline electrolytes.

Furthermore, we used *operando* Raman spectroscopy to support our conclusions extracted from the previously mentioned experiments. Our goal is to derive a model that combines the precatalyst transformation, the potential-dependent phase transitions of the different relevant Ni (oxy-)­hydroxide species, and the role of the remaining unreacted nickel selenide material in the OER performance of the active species.

## 2 Experimental Section

The experimental details of the electrodeposition, characterization, and electrochemical as well as spectroscopic investigations of the electrodeposits are provided in the Supporting Information (SI-1).

Electrodeposition of the precatalyst was selected because it provides good adhesion and electrical contact to the gold-modified carbon support, allowing for the deposition of a well-defined amount of precatalyst by terminating the process after a preset charge is passed.^25,26^

All OER experiments were conducted in 100 mM KOH + 1 mM Fe­(NO_3_)_3_ according to the procedure used in previous publications.^27,28^ Noteworthy, the presence of Fe significantly boosts the catalytic activity of Ni oxyhydroxide-based catalyst systems, whereas pure NiOOH exhibits only low OER activity, as it has been shown, e.g., by the group of Boettcher.^27,29,30^ Thus, we used an electrolyte with a similar KOH:Fe ratio as used in a study of Trotochaud et al.^27^

## 3 Results and Discussion

### 3.1 *Ex Situ* Characterizations

Carbon paper was chosen as a typical X-ray transmissive substrate.^31,32^ To improve the deposition efficiency and to exploit the synergistic effects in activity enhancement of nickel (oxy)­hydroxide-based OER electrocatalysts, gold^33–35^ was electrodeposited on the uppermost fibers of the carbon paper (see the SI for details). As depicted in Figure 1a, a nanoparticulate film with particles, tens to hundreds of nanometers in size, was formed by the potentiostatic deposition from a precursor solution containing NiSO_4_ and SeO_2_.^36^ Electrochemical OER investigations with such nickel selenide films were carried out in 100 mM KOH solutions to which 1 mM of Fe­(NO_3_)_3_ was added (see SI-1.4 and Figure S8), since the presence of Fe ions significantly enhances the catalytic activity of nickel chalcogenide-based catalysts.^16,27,37–39^ A nickel selenide deposit on an Au rotating disk electrode under similar conditions to the one on Au-modified carbon paper showed a Tafel slope of ≈26 mV dec^–1^ (at 4000 rpm, Figure S20a).^40^ This agrees well with earlier results for similar OER electrocatalysts of high activity.^5,41,42^

**Figure 1:**
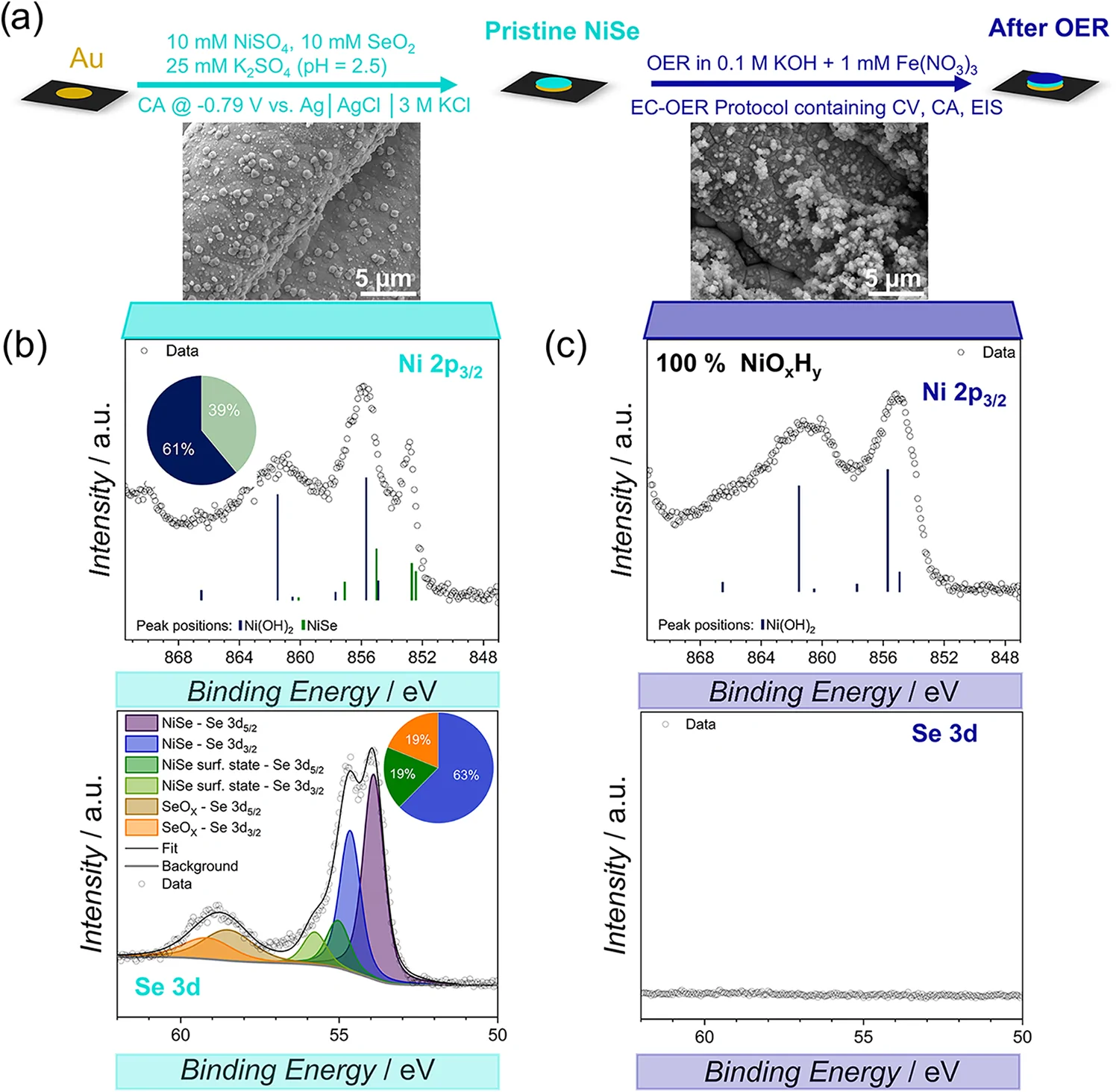
(a) Schematic deposition route of nickel selenide and transformation under OER conditions on Au-modified carbon paper with SEM images in pristine and after-OER states; (b, c) *ex situ* XPS of the Ni 2p_3/2_ and Se 3d signals of (b) pristine deposit and (c) the sample after OER. The peak positions and ratios of NiSe and Ni­(OH)_2_ reference samples in the Ni 2p_3/2_ spectra are marked by green and dark blue bars, respectively.

We conducted *ex situ* XPS measurements to characterize the surface of the pristine and after-OER samples. The experimental conditions and details of the data analysis are provided in SI-1.5 and SI-2.1, respectively. By integration of the Ni 3p and Se 3d signals in the XPS survey spectrum of the pristine sample (Figure S18c), a molar ratio of Ni:Se of ≈54%:46% was observed (Table S14). To characterize the nickel selenide species formed on the surface of the pristine sample, the Ni 2p_3/2_ and Se 3d signals were analyzed and compared to NiSe and Ni_3_Se_2_ reference samples.^28^

The Ni 2p_3/2_ and Se 3d XPS signals of the pristine sample in Figure 1b are in good agreement with NiSe reference nanoparticles of hexagonal structure. More than 80% (63% NiSe reference and 19% surface state NiSe with undercoordinated Se) of the Se content within the information depth can be assigned to NiSe (see SI-1.5 and SI-2.1 for details). The remaining part is found as SeO_2_, corresponding to the occurrence of Ni­(OH)_2_ in the Ni 2p_3/2_ spectrum and is a result of surface oxidation under ambient conditions.

For the sample after OER, no Se signal was detected by XPS (Figure S18d and Table S14), indicating that the near-surface layer probed by XPS was converted to Ni–O species under OER conditions.^4–7^ Although the Ni 2p_3/2_ and Se 3d signals recorded after OER (Figure 1c) are not a perfect fit for the Ni­(OH)_2_ reference data (Figure S12 and Tables S6, S7), no other nickel (oxide) species resulted in a better fit. Further, the comparison of the O 1s and Ni LMM signals to the nickel selenides deposited under similar conditions on a different substrate (see SI-2.1.1 for details) indicates that the character of the surface species of the sample after OER is similar to Ni­(OH)_2_ (Figure 1c).^43–45^

Noteworthy, both, pristine NiSe and the material after OER, show no signals in XRD other than those related to graphite or gold (Figure S22). We additionally utilized (scanning) transmission electron microscopy ((S)­TEM) to characterize the crystallinity and chemical composition of the pristine sample at a local scale (SI-1.7 and SI-2.3). High-resolution (S)­TEM and the corresponding fast Fourier transform (FFT) analyses demonstrate that the electrodeposited material is nanocrystalline, with reflections matching the hexagonal NiSe phase (Figures 2a and S23). Furthermore, energy-dispersive X-ray (EDX) mapping was conducted to verify the material’s stoichiometry (Figure S24), finding it in excellent agreement with that obtained by XPS.

**Figure 2:**
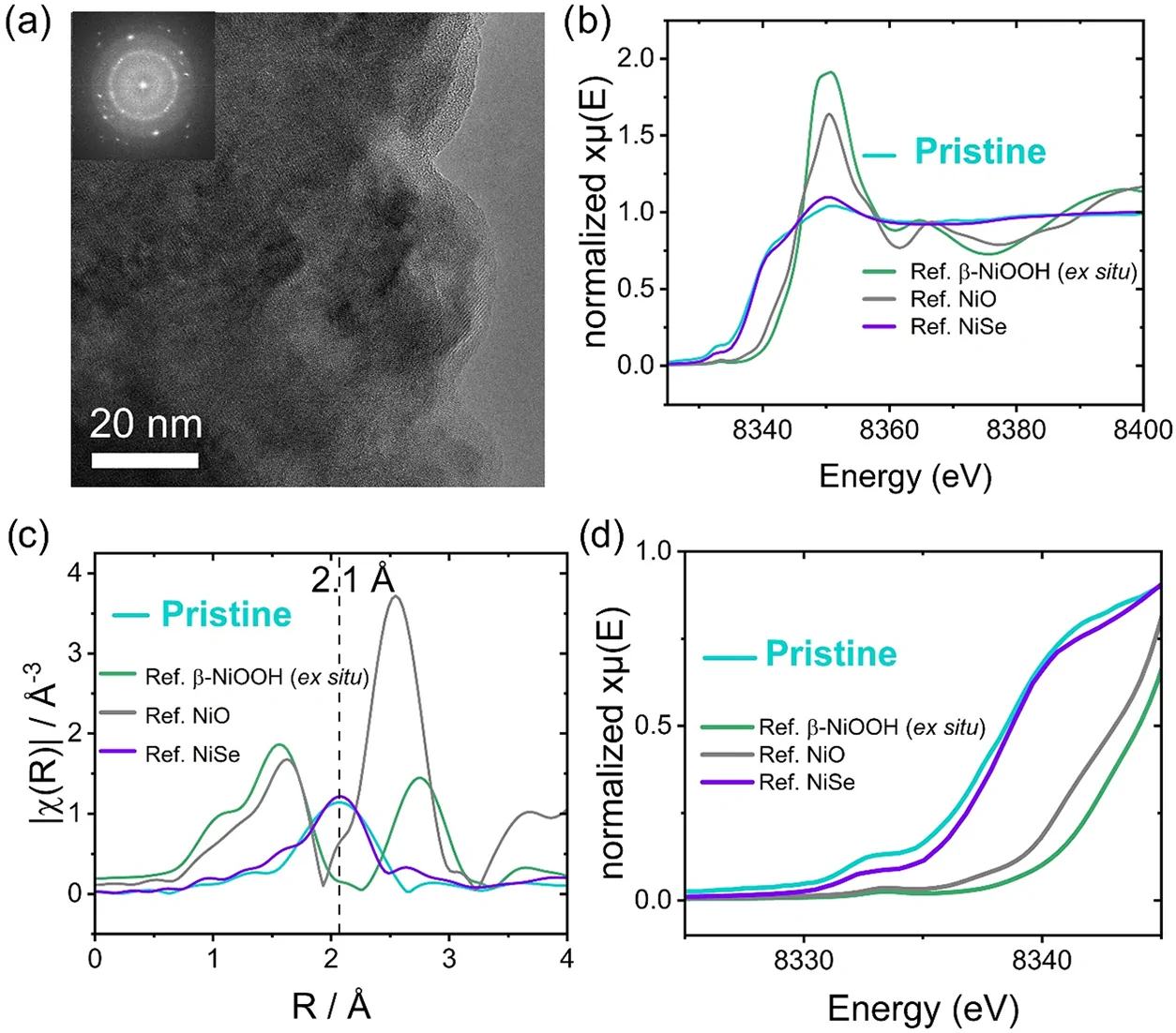
(a) TEM micrograph and corresponding FFT (inset) showing the polycrystalline structure of NiSe grown on Au-modified carbon paper; (b) XANES region of the *ex situ* XAS spectra at the Ni K-edge of the pristine NiSe in comparison to reference materials; (c) Fourier transforms in R-space extracted from the EXAFS region of the *ex situ* XAS spectra at the Ni K-edge show the typical Ni–Se bond distance (≈2.1 Å)^4,14,46^ for the pristine sample; and (d) magnification of the pre-edge region of XANES spectra in panel (b).

Hence, we conclude from the XPS, XRD, and (S)­TEM data that nanocrystalline NiSe is deposited, which transforms into NiO_*x*_H_*y*_, most likely Ni­(OH)_2_ near the surface under OER conditions.

To investigate the bulk oxidation states and local coordination environment of the pristine NiSe deposit, e*x situ* XAS measurements at the Ni K-edge were employed. In contrast to XPS, which is a surface-sensitive method that only probes a few nanometers into the sample material,^47^ hard XAS can probe the bulk material as well.^48^ Figure 2b shows that the pristine NiSe deposit matches the NiSe reference in the X-ray absorption near-edge structure (XANES) pre-edge region. The pre-edge feature of the pristine deposit at ≈8333 eV and near-edge region at ≈8340 eV in Figure 2d indicate that the local electronic structure, oxidation state, and site symmetry of Ni in the deposited film are consistent with NiSe standards.^28^ In Figure 2c, the Fourier-transformed extended X-ray absorption fine structure (EXAFS) in radial (R-) space highlights the typical bond distance of ≈2.1 Å for Ni–Se bonds in nickel selenide species^4,14,46^ present in pristine and NiSe reference samples. Beyond this short-range region, however, the R-space spectra display only weak or absent long-range features, which is consistent with the nanocrystallinity of the sample observed by XRD and (S)­TEM (Figure 2a and SI-2.3).

To further verify the phase purity of the electrodeposited material, linear combination fittings (LCF) of the pristine sample were performed, using a library of relevant Ni-containing reference spectra (Figure S25). The best fit was obtained using only the NiSe standard, with no detectable contribution from alternative Ni phases such as NiO, mixed-valence β-Ni­(OH)_2_/β-NiOOH (ref β-NiOOH (*ex situ*) in Figure 2b–d; see SI-2.5 and Figure S26 for further explanations of the character of the reference material), or metallic Ni. The LCF reproduces both the edge and pre-edge regions with low discrepancy, indicating that the deposited sample is well-described by a single NiSe environment.

Overall, the characterization by XPS, XRD, (S)­TEM, and XAS indicates that the electrodeposited pristine sample has a nanocrystalline hexagonal NiSe structure, passivated by a thin surface Ni­(OH)_2_ layer at ambient conditions. After OER, XPS suggests a transformation of NiSe into NiO_*x*_H_*y*_ species, most likely Ni­(OH)_2_, which results from self-discharge from NiOOH,^49^ and was further investigated in *operando* conditions.

### 3.2 *Operando* XAS Investigations

We conducted *operando* XAS at the Ni K-edge of the deposited NiSe to determine the oxidation state of Ni under reaction conditions. Figure 3a,b shows the changes in the XANES region and Fourier-transformed R-space at OCP and applied potentials *E*_1_ (1.37 V_RHE_) to *E*_4_ (1.57 V_RHE_), respectively. The investigated potentials are indicated in the CV curves recorded in the activation period in Figure 3c and the full electrochemical protocol is described in SI-1.4. After electrochemical activation of the catalyst, the XANES region of the catalyst spectrum suggests the oxidation state of the catalyst is similar to the mixed-valence β-Ni­(OH)_2_/NiOOH reference material (ref. β-NiOOH (*ex situ*) with a calculated baseline oxidation state of ∼2.5+ to ∼2.7+, see SI-2.5) under open-circuit potential (OCP) conditions, as shown in Figure 3a. Applying increasingly positive potentials to drive the OER, the edge positions of the *operando* spectra shift to a higher energy, revealing an increasing oxidation state, and eventually reach a position close to the nickel 4+ standard (as shown in Figure S28a). When reversing the potential back to the OCP, the initial XAS spectrum is recovered, indicating a reversible transformation of the catalyst back to a mixed-valence β-Ni­(OH)_2_/β-NiOOH-like resting state, with an average oxidation state of ∼2.5+ (Figure 3a). This is also in agreement with the Fourier-transformed R-space plot in Figure 3b.

**Figure 3:**
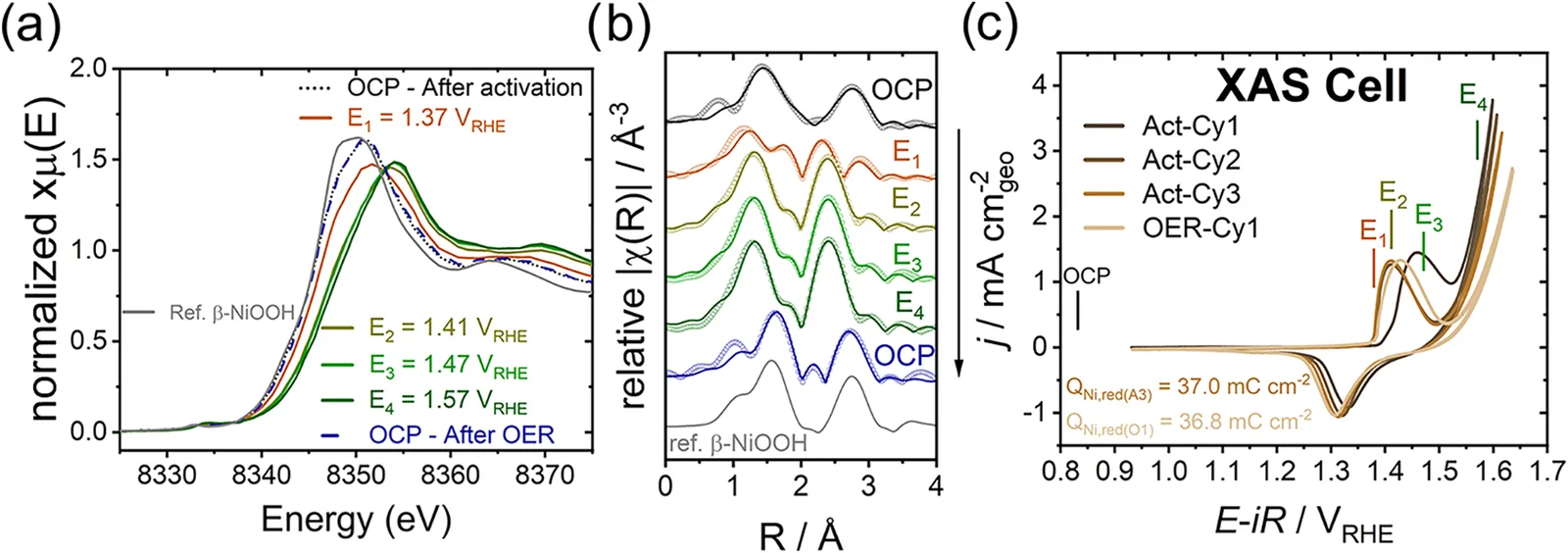
(a) *Operando* XANES spectra and (b) Fourier transforms of *operando* EXAFS spectra in R-space on the Ni K-edge of the electrocatalyst at different potentials; (c) cyclic voltammogram of the activated electrocatalyst, indicating the potentials chosen for *operando* investigations in panels (a) and (b).

Table 1 summarizes the Ni–O coordination numbers and bond distances obtained from EXAFS fitting, and Ni oxidation states derived from three different methods (described in Figure S28 and Tables S18–S21) at different applied potentials. The full data are provided in Tables S12–S14. At OCP, an average oxidation state of Ni of ≈2.7+ is observed for the catalyst, indicating a mixture of Ni hydroxide (Ni^2+^ dominant) and (oxy)­hydroxide species (Ni^3+^ dominant).^37^ This matches the findings from EXAFS R-space spectra, where the curve at OCP shows a good agreement with the β-NiOOH (*ex situ*) reference, which is in fact a mixed-valence β-Ni­(OH)_2_/β-NiOOH phase (Figure 3b). An average Ni–O distance of around 2.0 Å for the sample at OCP is obtained by EXAFS fitting (Tables 1 and S20).^50^ When the potential is increased to the onset of the voltammetric Ni oxidation wave (*E*_1_ = 1.37 V_RHE_; Figure 3c), the average Ni oxidation state increases to above +3, and the average Ni–O distance decreases to 1.91 Å. This implies a (nearly) quantitative oxidation of Ni­(OH)_2_ to NiOOH at these potentials. When further increasing the potential to the Ni redox peak maximum (*E*_2_ = 1.41 V_RHE_), the average Ni oxidation state increases to ≈+3.6. The coordination number estimated from EXAFS fitting suggests that >60% of the Ni content reached an oxidation state of +4 (Table 1). The +4 oxidation state agrees with predictions of Ni^4+^ based on DFT calculations, although the experimental data of the same authors showed a lower average oxidation state of Ni in Ni­(Fe)­OOH species.^17,18^ Notably, Fe^3+^ was reported to change the rate-determining step of nickel oxide-based OER electrocatalysts and stabilize Ni in lower oxidation states.^39,51,52^ Thus, the direct detection of high-valent Ni states at the Ni K-edge in catalysts with increased Fe^3+^ content appeared difficult.^17,38,53^ Since our samples only contain about 2 at% Fe relative to Ni (Figure S8), it seems reasonable that a significant fraction of nickel is present in the Ni^4+^ state.

**Table 1:** Coordination Numbers, Average Ni–O Distances, and Oxidation States of the Electrocatalyst, Extracted from EXAFS, XANES, and Ni–O Calibration, Respectively, Under *Operando* Conditions^a^

	coordination number EXAFS			averaged Ni–O distance	oxidation state	oxidation state	oxidation state
	Ni^2+^	Ni^3+^	Ni^4+^	Å	from EXAFS	from XANES	from Ni–O calibration
OCP = 0.82 V_RHE_	1.6 ± 0.9	3.3 ± 1.1		2.05 ± 0.04	2.7 ± 0.1	2.7 ± 0.2	2.2 ± 0.3
*E*_1_ = 1.37 V_RHE_		2.5 ± 0.9	3.3 ± 0.9	1.91 ± 0.02	3.5 ± 0.1	3.1 ± 0.2	3.4 ± 0.2
*E*_2_ = 1.41 V_RHE_		1.4 ± 0.8	3.6 ± 0.8	1.90 ± 0.02	3.7 ± 0.1	3.6 ± 0.2	3.7 ± 0.3
*E*_3_ = 1.47 V_RHE_		1.6 ± 1.4	3.6 ± 0.8	1.89 ± 0.05	3.7 ± 0.2	3.6 ± 0.2	3.7 ± 0.7
*E*_4_ = 1.57 V_RHE_		1.7 ± 0.9	3.3 ± 0.9	1.89 ± 0.03	3.7 ± 0.1	3.8 ± 0.2	3.7 ± 0.4
OCP (after OER)	4.0 ± 0.1	1.5 ± 0.1		1.97 ± 0.01	2.3 ± 0.1	2.8 ± 0.2	2.8 ± 0.1

a The oxidation states from EXAFS were obtained by calculating average oxidation states based on their coordination proportion. The methods to obtain oxidation states from XANES and Ni–O calibration curve are described in Figure S29 and Tables S18–S21.

When the potential is further increased to drive the OER, only a minor increase in the average Ni oxidation state and a slight decrease in the average Ni–O distance to ≈1.89 Å are observed (Table 1). Thus, the *operando* XAS investigations indicate that Ni^3+^/Ni^4+^ species, which are already formed at pre-OER potentials, are dominant under OER conditions. This finding is in good agreement with earlier studies by the Dau group.^17,18^

Some literature reports^54,55^ still assign the electrochemical Ni redox wave in CV experiments to the transition of Ni­(OH)_2_ into NiOOH, implying a pure Ni^2+^/Ni^3+^ transition. We herein accentuate that the assignment of the pure Ni^2+^/Ni^3+^ transition to the Ni redox wave in CV data is misleading. Instead, Ni is present in oxidation states +2, + 3, and +4 at these potentials.

EXAFS fitting of the *operando* spectra remains most consistent with models containing both Ni–O and residual Ni–Se coordination environments (Figure S28 and Tables S20 and S21). Although the Ni–Se contribution becomes significantly attenuated due to the formation of dominant Ni–O and Ni–Ni scattering paths during reconstruction, inclusion of residual Ni–Se coordination yields a structural model that is more consistent with the combined spectroscopic and compositional data set, including *operando* Raman measurements (see Section 3.4) and ICP-MS quantification showing retention of ≈45 ± 2% of the original selenium content after OER. Our results agree with previous *operando* EXAFS studies of Ni chalcogenide-based OER electrocatalysts, which found that the R-space spectra were dominated by Ni–O, suppressing the Ni–X (X = S, Se) signal.^56–58^

### 3.3 Coupled *Operando* EIS and XAS Investigations

Electrochemical impedance spectroscopy (EIS) was conducted simultaneously to the *operando* XAS measurements at different potentials, to link potential-dependent changes of the Ni oxidation state to the catalyst/electrolyte interphase, e.g., adsorption and transformation processes, and the resulting charge transfer kinetics and capacitances.

We recorded one EIS spectrum at the start and the end of the XAS scan at the respective constant potentials discussed in Section 3.2 (OCP, *E*_1_, *E*_2_, *E*_3_, and *E*_4_) to uncover transient changes of the catalyst, which could not be resolved on the longer time scale of the XAS experiments (see SI-1.4 for details). Figure 4 shows the Nyquist plots of the experimental EIS data and the equivalent circuits used for fitting the EIS data at the indicated potentials. A detailed description of the fitting procedure of the experimental EIS data using different equivalent circuits is provided in SI-2.6 (Figures S29–S33 and Table S24).

**Figure 4:**
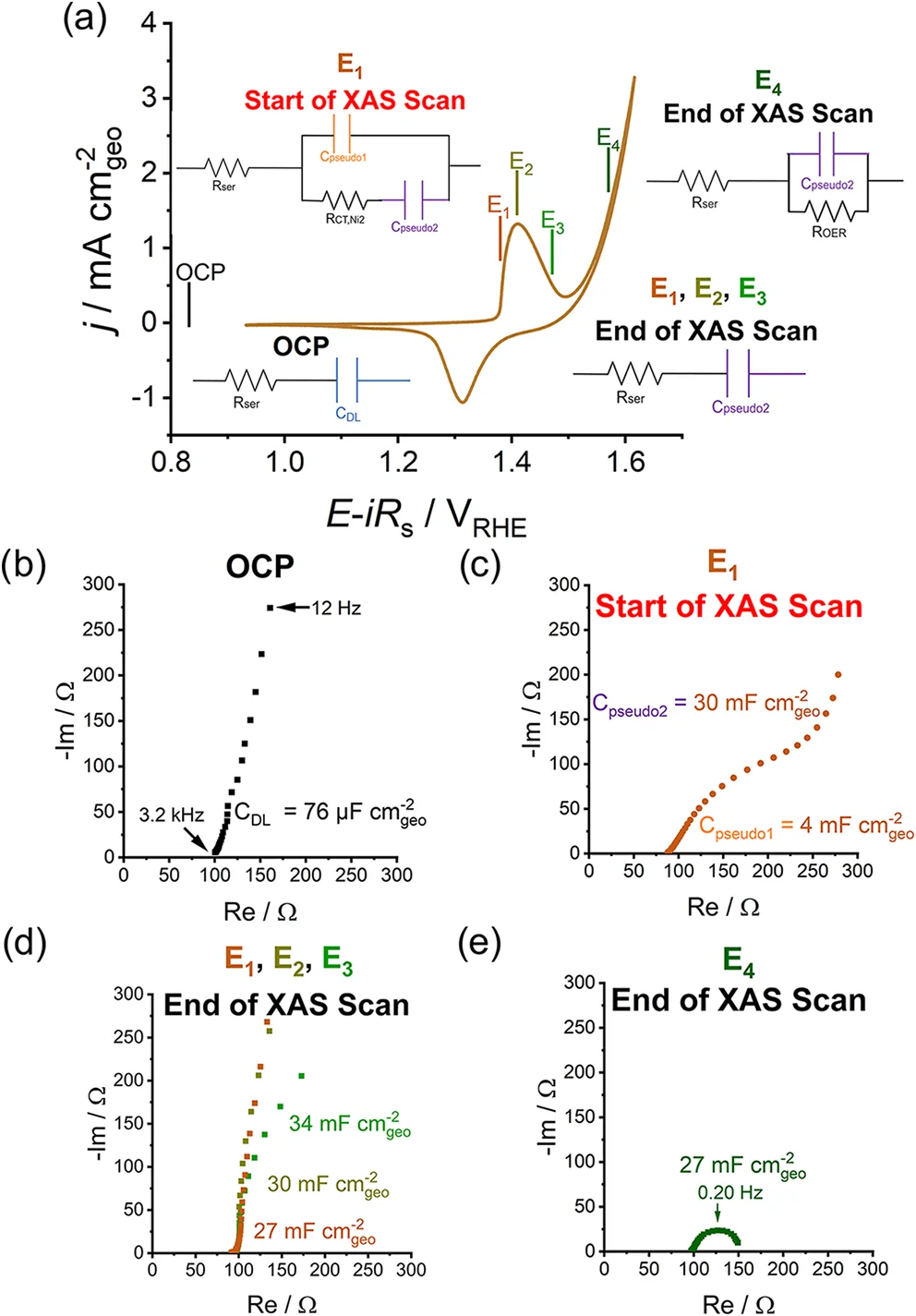
*Operando* EIS analysis at different potentials: (a) Potential-induced catalyst transformation results in different resistive and capacitive behaviors, as rationalized by the indicated distinct equivalent circuits used to fit the experimental data at the respective potentials. EIS spectra at (b) OCP (≈0.82 V_RHE_), (c) *E*_1_ = 1.37 V_RHE_ at the start of the XAS scan, (d) *E*_1_ = 1.37 V_RHE_, *E*_2_ = 1.41 V_RHE_, and *E*_3_ = 1.47 V_RHE_ at the end of the XAS scan, and (e) *E*_4_ = 1.57 V_RHE_ at the end of the XAS scan.

At OCP (≈0.82 V_RHE_) after activation (Figure 4b), the system can be described by an R–C circuit with a serial combination of the electrolyte resistance *R*_ser_ and double-layer capacity *C*_DL_ (Figure 4a). The extracted double-layer capacitance *C*_DL_ at this potential (≈76 μF cm_geo_^–2^) reflects the high electrochemically accessible surface area of the electrocatalyst on the Au-modified carbon substrate. When the potential is increased to the onset of the Ni oxidation peak, the EIS spectra differ significantly between the start and the end of the XAS scan at *E*_1_ = 1.37 V_RHE_, indicating a transient change in the chemical structure and associated processes at the catalyst/electrolyte interphase (Figure 4c+d). In the EIS spectrum, two R–C elements can be fit at the start of the XAS scan at E_1_ = 1.37 V_RHE_ (Figure 4c), whereas only one R–C element remains present for the spectrum at the end of the XAS scan (Figure 4d). The extracted capacitances are in the range of ≈4–34 mF cm_geo_^–2^. Since the detected double-layer capacity was in the μF cm_geo_^–2^ range, its contribution is negligible when applying a more positive potential. Directly after sweeping the potential to 1.37 V_RHE_, we find another resistance, which we assign to a charge transfer to the Ni centers, *R*_CT,Ni2_. We assign this resistance to the oxidation of Ni^3+^ to Ni^4+^, implicitly postulating that another charge transfer resistance, *R*_CT,Ni1_, exists, reflecting the oxidation of Ni^2+^ to Ni^3+^. However, *R*_CT,Ni1_ is assumed to be very small compared to *R*_CT,Ni2_ and thus negligible since a significant fraction of Ni centers is in the +3 state already at OCP (≈0.82 V_RHE_; Table 1). Two capacitances, *C*_pseudo1_ and *C*_pseudo2_, with *C*_pseudo1_ ≈4 mF cm_geo_^–2^ ≪ *C*_pseudo2_ (Table S24) being significantly smaller, are found only transiently when the potential reaches the onset of the Ni oxidation peak (*E*_1_ = 1.37 V_RHE_), and the first EIS spectrum is recorded at the start of the XAS scan. We assign *C*_pseudo1_ to the oxidation of Ni­(OH)_2_ to NiOOH with the adsorption of hydroxyl ions.^59^ This transformation could not be resolved using *operando* XAS due to the longer time scale of the experiment (see SI-1.4). Note that this finding at *E*_1_ = 1.37 V_RHE_ differs from all other potentials studied, where the EIS spectra at the start and the end of the XAS scan are similar (Figures S29 and S30). Thus, we conclude that the most severe transformation at the interface of the catalyst occurs at *E*_1_ = 1.37 V_RHE_.

After the completion of the oxidation process of Ni­(OH)_2_ to NiOOH, *C*_pseudo2_ remains the only capacitive element (EIS at the end of the XAS scan at *E*_1_ = 1.37 V_RHE_). We attribute this capacitance to the oxidation of NiOOH to higher Ni oxides (e.g., NiO_2_) with the adsorption of hydroxyl ions. The value of *C*_pseudo2_ remains relatively constant when the potential is further increased to *E*_2_ = 1.41 V_RHE_ and *E*_3_ = 1.47 V_RHE_ (Figure 4d), whereas no significant resistance other than *R*_ser_ is present, i.e., *R*_CT,Ni1_ and *R*_CT,Ni2_ are negligibly small. Notably, both *C*_pseudo1_ and *C*_pseudo2_ are pseudocapacitances, since they originate from charge transfer reactions, i.e., changes in Ni oxidation states.

When the potential is increased to *E*_3_, *C*_pseudo2_ rises due to the formation of higher Ni oxides on the surface and further accumulation of hydroxyl ions from *E*_2_ to *E*_3_. Under OER conditions (*E*_4_ = 1.57 V_RHE_), OH^–^ are transformed into surface-adsorbed intermediates. Additionally, to the slightly reduced *C*_pseudo2_ (27 mF cm_geo_^–2^) during the O_2_ evolution at *E*_4_, another resistance originates from charge transfer of the OER process. Accordingly, the semicircle-like Nyquist plot of the EIS data in Figure 4e can be described by a typical Randles circuit (Figure 4a, top right).^37^

Using *operando* XAS, we were able to determine the Ni oxidation state under an applied potential and found that the Ni^3+^/Ni^4+^ transition is already dominating at pre-OER potentials. *Operando* EIS showed two (pseudo)­capacitors, *C*_pseudo1_ and *C*_pseudo2_, in EIS spectra recorded directly after applying a potential of 1.37 V_RHE_ (foot of Ni oxidation wave). *C*_pseudo1_ vanishes when holding the potential for longer times, indicating that *C*_pseudo1_ is associated with a change of the Ni oxidation state within the electrode material.

Thus, we conclude the occurrence of *C*_pseudo1_ alongside *C*_pseudo2_ to be related to the oxidation of Ni­(OH)_2_ to NiOOH, which are still present when the potential is swept to *E*_1_ = 1.37 V_RHE_ (start of XAS scan). The charge transfer, accompanied by the adsorption of hydroxyl ions on Ni­(OH)_2_ during the transformation to NiOOH, might be responsible for the occurrence of the smaller pseudocapacitance.

### 3.4 *Operando* Raman Investigations

Due to the missing long-range order of the electrodeposited pristine nickel selenide (Figure 2 and SI-2.3) and the similarity of Ni­(OH)_2_ and NiOOH in XAS,^51,60,61^ we conducted *operando* Raman spectroscopy to follow the transformation of NiSe into Ni­(OH)_2_ and its subsequent transformation into NiOOH and higher Ni oxides in more detail. The experimental details of the *operando* Raman experiments are described in SI-1.8.

Figure 5a shows the sample in the pristine state (before activation), at OCP after activation, and at 1.37 V_RHE_ (onset of the Ni oxidation peak). For the pristine sample, signals were found related to the E_g_ (167 cm^–1^) and A_g_ (216 cm^–1^) vibrations in NiSe (enlarged region in Figure 5b).^2,62,63^ Those vibrations are absent for a pure Se deposit (Figure S36), confirming the identity of the pristine sample as NiSe. These spectra support our previous observation of a nanocrystalline NiSe structure in the pristine sample, in line with the discussion in Section 3.1.

**Figure 5:**
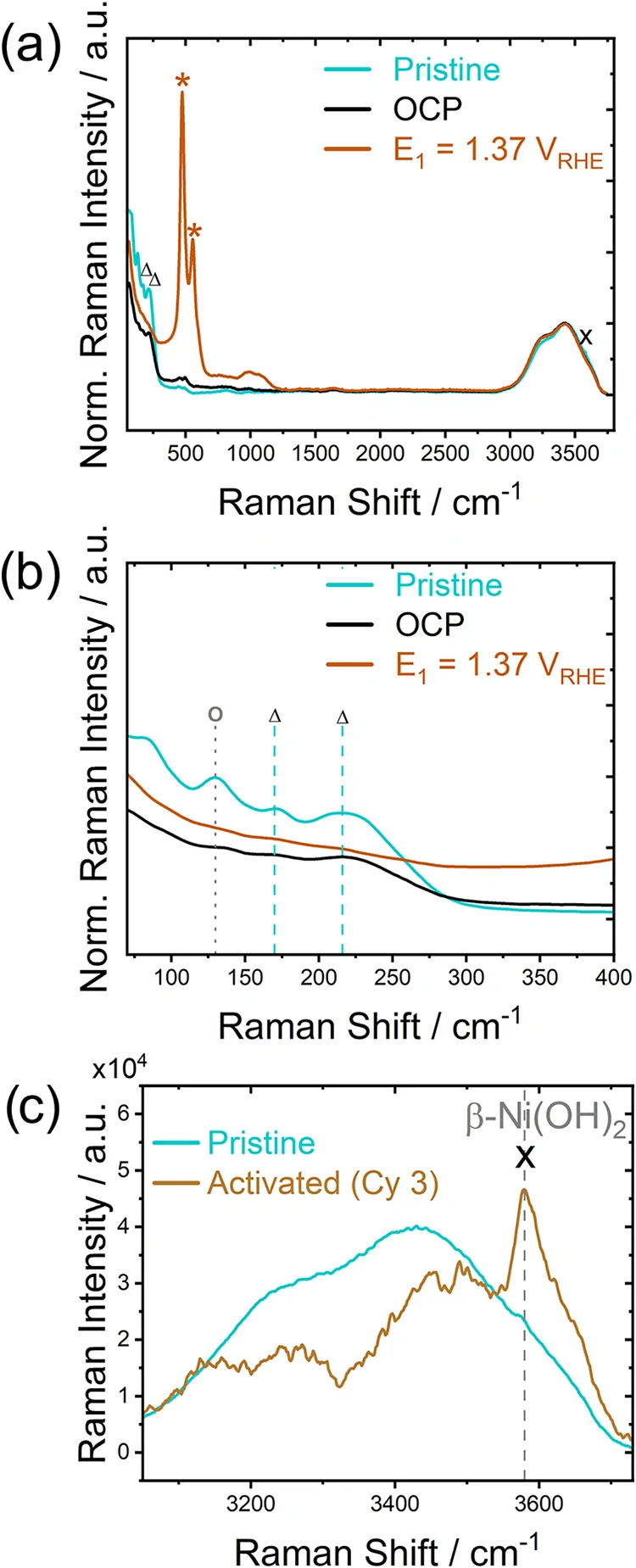
*Operando* Raman spectra of pristine sample, at OCP after activation, and *E*_1_ = 1.37 V_RHE_ in (a) full range and (b) enlarged range focusing on NiSe signals; (c) sum *operando* Raman spectra in the higher wavenumber range of the pristine and activated samples (third CV cycle during catalyst activation) with the evolution of the typical OH band in β-Ni­(OH)_2_ at 3580 cm^–1^ during activation.

While the potential is swept to 1.37 V_RHE_, the intensities of the OH-bending (471 cm^–1^) and OH-stretching (549 cm^–1^) bands in NiOOH dominate the spectrum.^62^ These bands are amplified due to known resonance effects.^64^ Their increase compared to the state after activation proves a significant formation of NiOOH occurring at the foot of the Ni oxidation wave, as concluded from the *operando* XAS data (Section 3.2). Furthermore, this finding supports the hypothesis derived from the *operando* EIS analysis that the transformation of Ni­(OH)_2_ into NiOOH might be responsible for the transient occurrence of a second, smaller pseudocapacitance at 1.37 V_RHE_, as suggested in Section 3.3.

Figure 5c shows the region of larger wavenumbers (3050–3730 cm^–1^) with the sum spectra of the pristine sample and the activated catalyst (sum spectrum of the third CV cycle). During the activation by potential cycling, a signal at 3580 cm^–1^ evolves. This signal is related to the OH-stretching vibration in β-Ni­(OH)_2_.^61,62,65^ Several studies report that less-ordered α-Ni­(OH)_2_/γ-NiOOH is converted into more-ordered β-Ni­(OH)_2_/β-NiOOH by aging and/or cycling in a moderate potential range, which is in line with our observations.^27,37^

Figure S37 shows that the intensity of this band increases with prolonged cycling when the potential is swept back to the range of the Ni reduction peak. Notably, the band at 3580 cm^–1^ remains visible in the third CV cycle even in the potential range of the Ni oxidation wave. Thus, we conclude that an increasing fraction of the Ni-containing electrode material is converted to β-Ni­(OH)_2_/β-NiOOH during prolonged OER.^62^

### 3.5 Proposed Transformation Scheme of Nickel Selenide

Based on our data comprising multiple *operando* measurements, we propose a transformation scheme for OER electrocatalysts derived from NiSe that rationalizes their superior performance to oxide- or hydroxide-derived electrocatalysts. As detailed in Sections 3.1 and 3.4, the pristine (pre)­catalyst exhibits a hexagonal *P*6_3_/*mmc* NiSe nanocrystalline structure,^59,60,66^ depicted in Figure 6 (left). Under ambient conditions or when applying positive potentials in an alkaline electrolyte, NiSe transforms into Ni­(OH)_2_, which is in a potential-dependent equilibrium with NiOOH. NiOOH is already the dominating species under pre-OER potentials, as shown in our *operando* experiments (Sections 3.2, 3.3, and 3.4). For simplicity, we assigned the Ni centers in Ni­(OH)_2_ to Ni^2+^ and in NiOOH to Ni^3+^ in the proposed transformation scheme (Figure 6). Furthermore, we included the crystal structure of β-Ni­(OH)_2_ (P3̅m1)^37^ since the presence of this phase under operation conditions was proven by *operando* Raman studies (Section 3.4).

**Figure 6:**
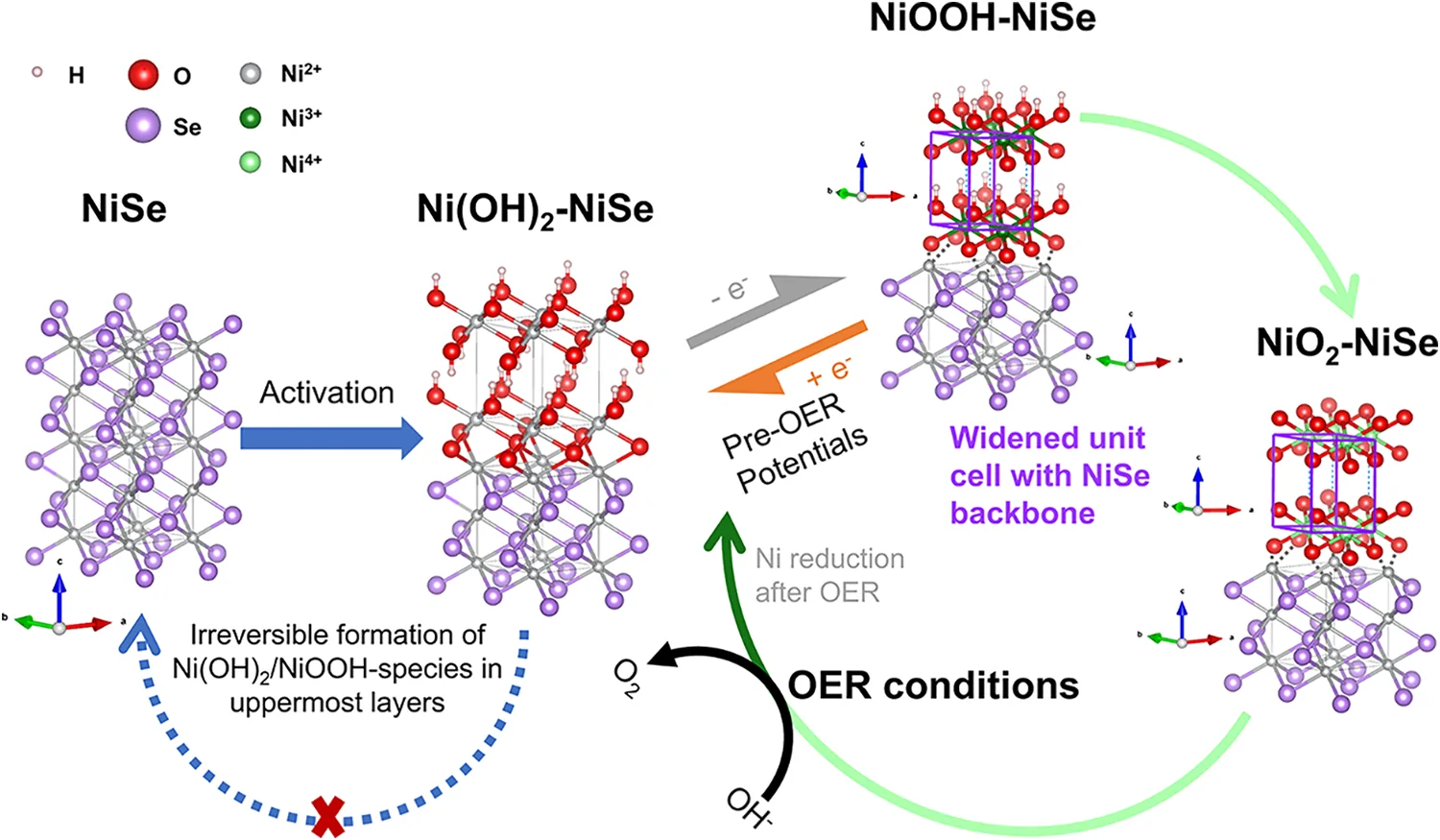
Schematic illustration of the catalytic cycle. Short-range order in NiSe (*P*6_3_/*mmc*) and in β-Ni­(OH)_2_ (P3̅m1) is assumed. During the activation, the uppermost layers of nickel selenide are irreversibly transformed to β-Ni­(OH)_2_, which is in a potential-dependent equilibrium with β-NiOOH (*C*2/*m*) at pre-OER potentials. Notably, the elongated average Ni–O distance of the β-NiOOH-NiSe species, originating from the presence of underlying NiSe, is assumed to slightly shift this potential-dependent equilibrium toward β-Ni­(OH)_2_-NiSe. Under OER conditions, Ni^4+^ species dominate. For simplicity, Ni centers are depicted in 2+, 3+, and 4+ states, neglecting that a significant number of Ni centers have already reached oxidation state 4+ at pre-OER potentials. We propose that the remaining unreacted nickel selenide assists in the reduction of the Ni centers to Ni­(OH)_2_ species as an instructive example for the role of frustrated phase transitions for high activity in heterogeneous (electro)­catalysis.

The transformation mechanism summarized in Figure 6 shows that the crystal structures of “backbone” NiSe and β-Ni­(OH)_2_ show very similar atomic configurations along some planes, irrespective of their different space groups. This may enable the observed spontaneous transformation to Ni­(OH)_2_ as a passivation layer under ambient conditions, which is further enhanced in alkaline electrolytes at positive potentials. However, ≈45 ± 2% of the original selenium content remains within the catalyst film after extended OER operation according to *ex situ* ICP-MS, indicating that reconstruction is incomplete and that a substantial fraction of the initial NiSe framework remains beneath the transformed surface layer. These remaining NiSe domains are not directly involved in the catalytic OER process (XPS shows no NiSe on the sample surface after OER, see Figure 1c, while ICP-MS probes the full film and with this also deeper sample layers).^5^ When β-Ni­(OH)_2_ is oxidatively transformed to β-NiOOH, the structure changes from cubic P3̅m1-like to monoclinic *C*2/*m*-like of an irregular prismatic and tetrahedral structure.^67,68^ O*perando* XAS investigations of our NiSe-derived catalyst showed an average Ni–O distance of 1.89 Å at pre-OER and OER potentials (Table 1), whereas previous studies reported 1.86 Å for selenide-free β-NiOOH under similar conditions.^69,70^ We hypothesize that the elongated average Ni–O distance in β-NiOOH on underlying NiSe, compared to pure β-NiOOH, indicates a frustrated phase transition of NiSe-supported β-Ni­(OH)_2_ into β-NiOOH. This is also in line with the EIS analysis in Section 3.3, showing a transient pseudocapacitance for the Ni^2+^/Ni^3+^ oxidation. Thus, we will denote the structure as the β-NiOOH-NiSe phase below when the underlying layers consist of NiSe.

Under OER conditions, the β-NiOOH-NiSe phase contains an increasing amount of higher Ni oxides, visualized as NiO_2_-NiSe in Figure 6. Notably, NiO_2_-NiSe is suggested here as a transient phase that only exists at reaction conditions under potential perturbation for a fraction of the Ni centers. We hypothesize that the structure is similar to that of the β-NiOOH-NiSe phase since it is formed by deprotonation of β-NiOOH-NiSe, accompanied by the release of O_2_.

A recent study by Sun et al.^56^ investigated several nickel chalcogenide OER electrocatalysts, including NiSe_2_, a nickel-selenide-based system with a different stoichiometry compared to the one used in our study. Sun et al.^56^ identified a lattice oxygen mechanism (LOM) on the NiSe_2_-based OER electrocatalyst by applying differential electrochemical mass spectrometry (DEMS) and supporting DFT calculations. To investigate a potential LOM contribution in the OER of the electrodeposited NiSe in this work, we performed DEMS experiments with ^18^O isotope-labeled water as well. A detailed description of the experimental conditions, data processing, and analysis (including a plausibility check) can be found in SI-1.8 and SI-2.8 (Figures S38 and S39). Our results show no significant change in the *m*/*z* = 34 signal and the mole fraction χ­(^16^O^18^O) being close to natural abundance. Thus, we concluded from three different DEMS measurements that the LOM is contributing with <10% in the OER on our electrodeposited nickel selenide (see SI-2.8 and Figures S38 and S39). This agrees with findings from a recently published paper of our group, where neither on Co–Cr-based materials, nor on RuO_2_, indications for a significant contribution of the LOM were found during OER under ambient conditions.^71^

Furthermore, previous experimental studies reported higher OER activity for α-Ni­(OH)_2_/γ-NiOOH structures than for β-Ni­(OH)_2_/β-NiOOH, which was attributed to higher disorder and larger defect concentration in α-Ni­(OH)_2_/γ-NiOOH, facilitating the oxidation of OER intermediates.^37,72^ In this work, the presence of unreacted NiSe might induce disorder and defects in the structure of the β-NiOOH-NiSe phase that cause a similar OER activity increase.^73,74^

We suggest that this enhancement of the OER activity can be rationalized based on local and dynamic phase transformations of the working catalysts, in line with the concept of frustrated phase transitions, which were introduced by Schlögl et al. for thermal catalysis.^75,76^ In this concept, heterogeneous catalysis is discussed as a convoluted interplay of two reaction networks: (i) the molecular transformation of the reactant to the product and (ii) the associated dynamic transformation and regeneration of the catalyst structure at active sites.

Herein, the molecular transformation is the conversion of OH^–^ to O_2_, involving the adsorption of reactants and intermediates alongside electron and proton transfers and product formation. These processes change the local chemical potential and oxidation state of the involved metal centers, which induce local and dynamic phase transformations of the working catalyst. This is the second reaction network,^75^ in our case, the frustrated (partial and dynamic) transformation of NiSe into β-Ni­(OH)_2_ and β-NiOOH at the catalyst surface. The unreacted subsurface NiSe causes an elongated average Ni–O distance in the β-NiOOH-NiSe phase, resulting in higher structural similarities with β-Ni­(OH)_2_. These similarities facilitate an easier transition between the two phases. Thus, the reduction of Ni^3+^/Ni^4+^ centers back to Ni^2+^ states, which restarts the catalytic cycle, is sped up since the barrier for local rearrangements is lowered in the presence of the NiSe backbone. The local transformation of the catalytic centers between different oxidation states and the associated phases are summarized schematically in the OER-catalytic cycle, as shown in Figure 6. While we identified these frustrated—and hence facile—phase transformations as the key role of NiSe in alkaline OER catalysis, also an influence of selenite and selenate oxyanions on the electronic structure of the active site has recently been reported.^9,42,77^ Scanning flow cell (SFC) ICP-MS experiments (Figure S40) we conducted in this respect suggest that the adsorption of dissolved selenium on the transforming catalyst may assist in promoting its catalytic activity.

We conclude that the NiSe “backbone” of the catalyst promotes the transient transformation of the frustrated catalyst between a Ni^4+^ state (represented as NiO_2_-NiSe) back to β-Ni­(OH)_2_ and β-NiOOH when O_2_ is liberated from the catalyst within the OER-catalytic cycle, which allows a speedy start of the next cycle. The mechanistic rationale depicted in Figure 6 may also allow us to understand earlier observations of enhanced OER-catalytic activity for β-NiOOH containing structural defects compared to as-synthesized β-NiOOH.^73,74^

## 4 Conclusion

In summary, we successfully electrodeposited nanocrystalline nickel selenide as hexagonal NiSe phase on a gold-modified carbon paper support and converted a significant fraction of this precatalyst during an anodic potential sweep in alkaline media to nickel oxyhydroxide species. Using XAS and EIS under *operando* conditions, we proved that the Ni^3+^/Ni^4+^ transition occurs at pre-OER potentials (1.37 V_RHE_) at the foot of the voltammetric Ni oxidation wave (1.37 V_RHE_). Our *operando* Raman investigations showed an increasing fraction of β-Ni­(OH)_2_ with ongoing CV conditioning, indicating that β-Ni­(OH)_2_/β-NiOOH evolved under operation conditions on the remaining NiSe precatalyst backbone. This is also corroborated by ICP-MS analysis of samples after OER catalysis, which showed a remaining Se content of ≈45 ± 2% compared to the pristine NiSe. Although well-ordered β-Ni­(OH)_2_/β-NiOOH was reported to exhibit lower OER activity than the less-ordered α-Ni­(OH)_2_/γ-NiOOH couple,^37,72^ the catalytic activity of β-Ni­(OH)_2_/β-NiOOH on underlying NiSe in this study is improved by disordering and defects in the crystal structure. Our *operando* XAS results showed a larger Ni–O bond length of 1.89 Å at high OER overpotentials than 1.86 Å of β-NiOOH materials without an NiSe backbone.^69,70^

The results of XPS, XAS, EIS, and Raman spectroscopy indicate that frustrated phase transitions facilitate rapid dynamic changes in the Ni oxidation states of the active centers during the catalytic cycle by avoiding a full and slow solid-state phase transformation to a catalytically less active, thermodynamically stable phase. We hypothesize that the key contribution of NiSe toward the outstanding OER performance of the nickel selenide-derived OER catalyst rests in enabling a dynamic structural evolution of the OER active Ni–O catalyst, accompanied by further effects discussed in earlier studies.

The concept of frustrated phase transitions has recently been demonstrated to govern thermal oxidation catalysis at metal oxides.^75,76^ We demonstrate herein that the approach can also be applied to electrocatalysis, thus suggesting frustrated phase transitions as a promising design criterion for the knowledge-driven development of metal oxide electrocatalysts.

## Supplementary Material



## Notes

A detailed experimental section with the full electrochemical protocol, additional electrochemical activity data, all details of XAS, XPS, Raman, SEM/EDX measurement settings, and analysis details (PDF)

## #

Faculty of Mechanical Engineering, Chair of Atomic Scale Characterization, Surface Science & Electrochemistry, Ruhr University Bochum, 44801 Bochum, Germany

## Abbreviations

OER: oxygen evolution reaction
XAS: X-ray absorption spectroscopy
XANES: X-ray absorption near-edge structure
EXAFS: extended X-ray absorption fine structure
EIS: electrochemical impedance spectroscopy
XPS: X-ray photoelectron spectroscopy
DFT: density functional theory
TEM: transmission electron microscopy
FFT: fast Fourier transform
LCF: linear combination fitting
OCP: open-circuit potential
LOM: lattice oxygen mechanism
DEMS: differential electrochemical mass spectrometry
SFC-ICP-MS: scanning flow cell inductively coupled plasma mass spectrometry
