# Supplementary material for: Embracing Phase Transitions to Rationalize the High Oxygen Evolution Reaction Activity of Nickel Selenides in Alkaline Electrolytes
Source: J Am Chem Soc. 2026 Nov 2;148(44):47773–84. doi: 10.1021/jacs.6c09277 (PMC13761207; doi:10.1021/jacs.6c09277)
Supplement: Supplementary file 1 [file ja6c09277_si_001.pdf]

Supporting Information (SI): Embracing Phase Transitions to Rationalize the High Oxygen Evolution Reaction Activity of Nickel Selenides in Alkaline Electrolytes

*Felix Hiege,[a] Chun-Wai Chang,[b] Oliver Trost,[a] Pouya Hosseini,[c†] Sheila Hernandez,[a] Carsten Dosche,[d] Gabriel Boitel-Aullen,[a] Raquel Aymerich-Armengol,[a] Christoph J. Bondue,[a] Andrea M. Mingers,[c] Jan Clausmeyer,[a] Alvin Chang,[b] Qing Ma,[e] Gunther Wittstock,[d] Zhenxing Feng[*b] and Kristina Tschulik[*a,c]*

[a]Faculty of Chemistry and Biochemistry, Chair of Analytical Chemistry, Ruhr University Bochum, Bochum, Germany. E-mail: kristina.tschulik@rub.de

[b]School of Chemical, Biological, and Environmental Engineering, Oregon State University, USA. E-mail: zhenxing.feng@oregonstate.edu

[c]Max-Planck-Institut für nachhaltige Materialien GmbH, Max-Planck-Straße 1, 40237 Düsseldorf, Germany

[d]Institute of Chemistry, School of Mathematics and Science, Carl von Ossietzky University Oldenburg, Ammerländer Heerstrasse 114-118, 26129 Oldenburg, Germany

[e]DND-CAT, Synchrotron Research Center, Northwestern University, Evanston, IL, 60208, USA.

Content

1. Detailed Experimental Procedures S3
   1.1 Electrochemical Setup S3
   1.2 Fabrication and Pre-treatment of Gold-modified Carbon Paper Substrates S3
   1.3 Electrodeposition of Nickel Selenide Films S4
   1.4 *Operando* and *Ex situ* X-ray Absorption Spectroscopy Experiments S5
   1.5 *Ex situ* X-ray Photoelectron Spectroscopy S8
   1.6 E*x situ* XRD Experiments S9
   1.7 TEM Experiments S10
   1.8 DEMS Experiments S10
   1.9 RDE Experiments S11
   1.10 *Operando* Raman Spectroscopy S12
   1.11 *Operando* and *Ex situ* ICP-MS Investigations S14
   1.12 SEM/EDX Investigations S15
   1.13 Data Processing S15
   1.14 Visualisation of Crystal Structures S15
2. Supplementary Experimental Data S16
   2.1 XPS Data S16
      2.1.1 Iron Content in NiSe Material after OER in Fe-Supplemented Electrolyte Solution S16
      2.1.2 Reference spectra of NiSe and Ni_3Se_2 Samples and Peak Models S16
      2.1.3 Nickel Selenide Films Deposited on Au Modified Carbon Paper S20
      2.1.4 Nickel Selenide Films Deposited on Au-Coated Glass Slides S23
      2.1.5 Details of O 1s Peak Fitting Deposited on Au-Coated Glass Slides and Au-Modified Carbon Paper S26
      2.1.6 Comparison of the Ni LMM Auger Peak of the Samples after OER S28
      2.1.7 Survey Scans of the Nickel Selenide Films S29
      2.1.8 XP-Spectra of Selenium Dioxide S30
   2.2 Au-RDE vs. XAS-Cell S32
   2.3 XRD Data S35
   2.4 TEM Data S36
   2.5 XAS Data S37
   2.6 EIS Data S45
      2.6.1 Explanations Regarding EIS Analysis S48
      2.6.2 Reproducibility of the EIS Fitting Using Constant Phase Elements (CPEs) S53
   2.7 Raman Data S56
   2.8 DEMS Data S57
   2.9 SFC-ICP-MS Data S61
References S61

1. Detailed Experimental Procedures

1.1 Electrochemical Setup

Electrochemical measurements were conducted using a three-electrode setup using different potentiostats. If not stated differently, a carbon rod (d = 6 mm) was used as counter electrode (CE). For the measurements in alkaline electrolytes in the XAS cell and RDE experiments, a commercial Hg|HgO reference electrode (RE) (ALS RE61-AP) filled with 0.1 M KOH in the body was used. In some experiments, an in-house-made Ag|AgCl|3 M KCl RE was used; its potential shift was verified to match an accuracy of at least ±5 mV against a commercial calomel electrode in 3 M KCl before and after any experiment.

1.2 Fabrication and Pre-treatment of Gold-modified Carbon Paper Substrates

Gold was electrodeposited on carbon paper (Toray Carbon Paper, PTFE treated, TPG-H-60) from a commercial Au-plating bath (Jenter Plating Technology, JE250, c_{Au} = 5 g L^{-1}) in a Teflon cell using a PalmSens4 potentiostat (PalmSens). The circular area of the carbon paper with a diameter of 12 mm was exposed to the plating bath under chronoamperometric conditions at -1.2 V vs. an in-house-made RE (Ag|AgCl|3 M KCl) for 1 h. Mass-transport was enhanced using an in-house-made syringe pump by inducing convection in the plating bath.

Figure S1 shows that Au was deposited on the surface of the carbon paper substrate exposed to the Au-plating bath.

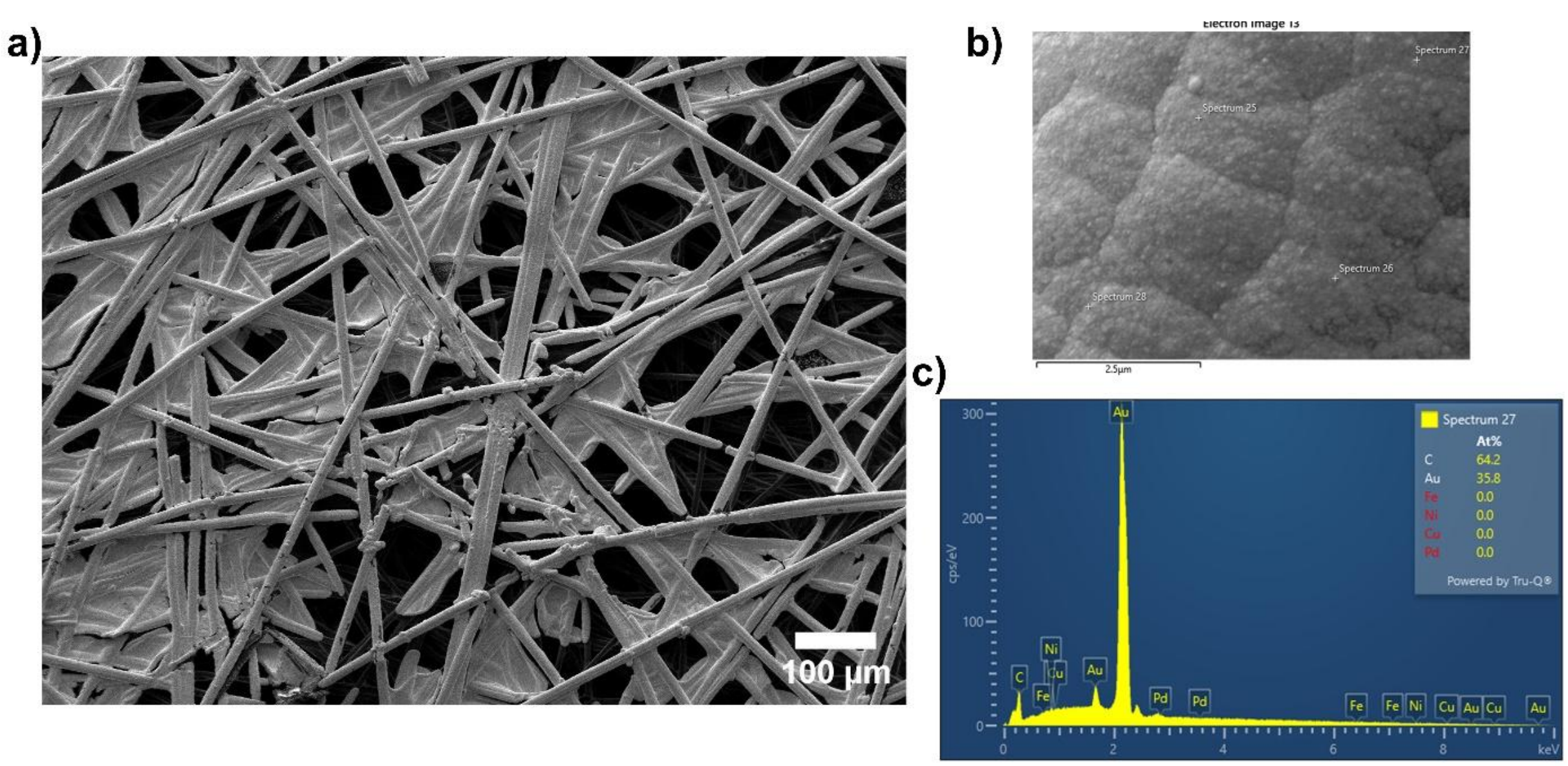

Figure S1. a) SEM image of the carbon paper with electrodeposited Au; b) + c) EDX data verifying the Au deposition.

Before the electrodeposition of nickel selenides, 20 CV cycles were monitored on the Au-modified carbon papers in 0.5 M H_2SO_4 in a potential window of −0.1 V to 1.45 V vs. Ag|AgCl|3 M KCl at a scan rate of 0.1 V s^{-1} with an initial and final potential of 0.2 V to 1) achieve a clean working electrode by multiple oxidation and reduction of the gold surface and 2) estimate the microscopic area by integrating the gold reduction peak of the last CV cycle and dividing the charge by the specific charge of 482 μC cm^{-2} for a chemisorbed gold oxide monolayer on a polycrystalline gold surface.[1] The experimental cleaning CV and the calculated microscopic area A_m of the Au-modified carbon paper used in the operando XAS study are depicted in Figure S2.

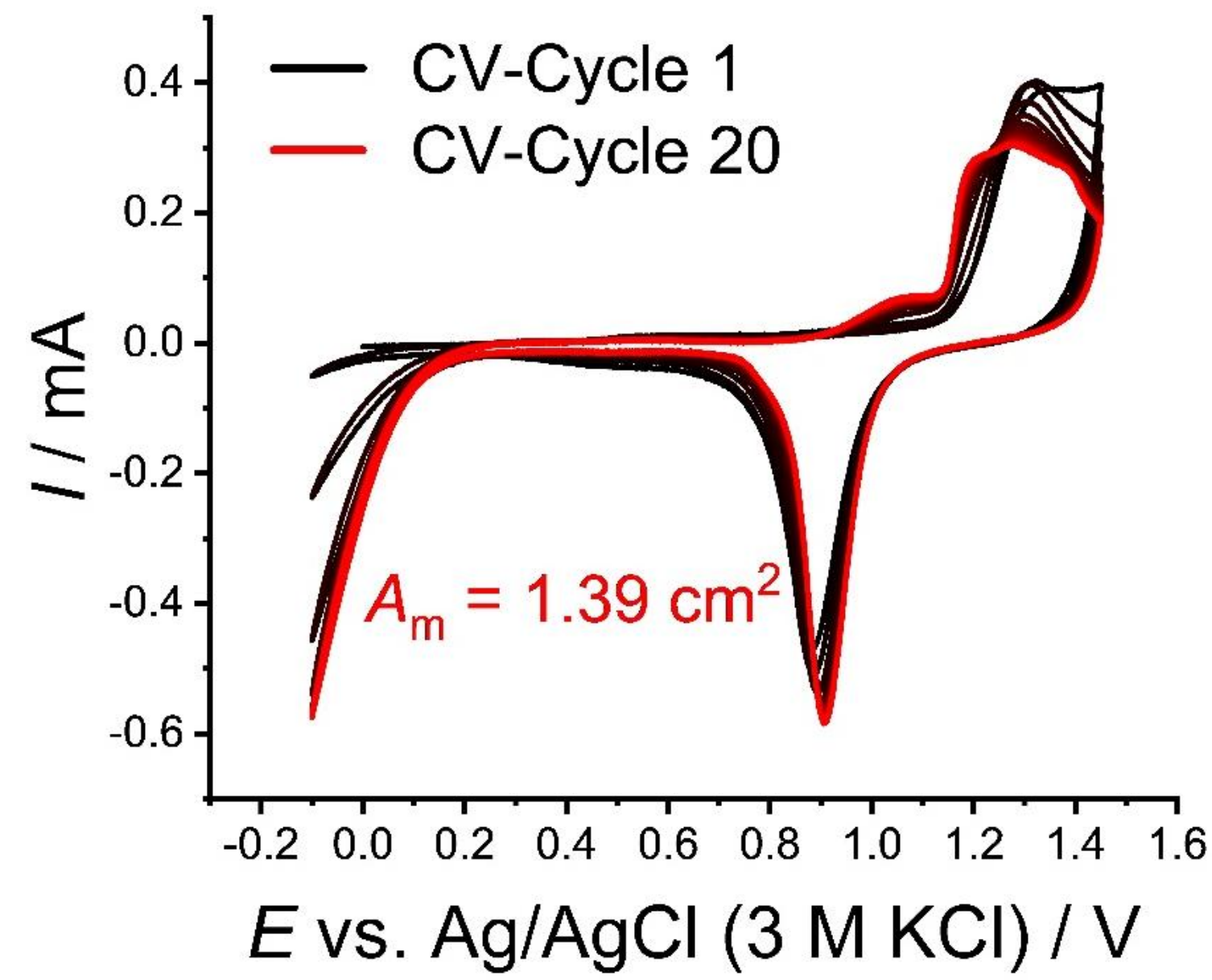

Figure S2. 20 CV cycles of the Au-modified carbon paper in aqueous 0.5 M H_2SO_4 in a potential window of −0.1 V to 1.45 V vs. Ag|AgCl|3 M KCl (initial/final potential 0.2 V) at a scan rate of 0.1 V s^{-1}. The microscopic area A_m of the Au deposit was calculated by obtaining the charge for the surface gold oxide reduction of the 20th potential cycle.

1.3 Electrodeposition of Nickel Selenide Films

The electrodeposition procedure of nickel selenides was based on the procedure of Cao et al.[2] The deposition solution contained 10 mM $NiSO_4 \cdot 6H_2O$ (Sigma Aldrich, ≥99.99 % trace metal basis), 10 mM SeO_2 (Sigma Aldrich, 99.9 % metal basis), and 25 mM K_2SO_4 (Supleco) in ultra-pure water (< 0.055 μS cm). The pH of the deposition solution was 2.5, and the solution was deoxygenated under Ar-flow for at least 20 min before each deposition.

The electrodepositions were performed in chronoamperometric experiments using three-electrode setups with an in-house made Ag|AgCl|3 M KCl RE. Additionally, a double junction containing 25 mM K_2SO_4 solution was linked to the RE, to prevent chloride contamination of the deposition solution. As working electrodes (WE), different substrates were used depending on the subsequent experiment (see following sections). All electrodeposition experiments were conducted at a constant potential of −0.79 V vs. Ag|AgCl|3 M KCl with coulometric termination control to achieve a charge loading of ca. 1.1 C cm^{-2}_{geo} based on the geometric area of the WE. Figure S3

shows the chronoamperometric deposition curve on Au-modified carbon paper, which was used for operando XAS characterization afterwards.

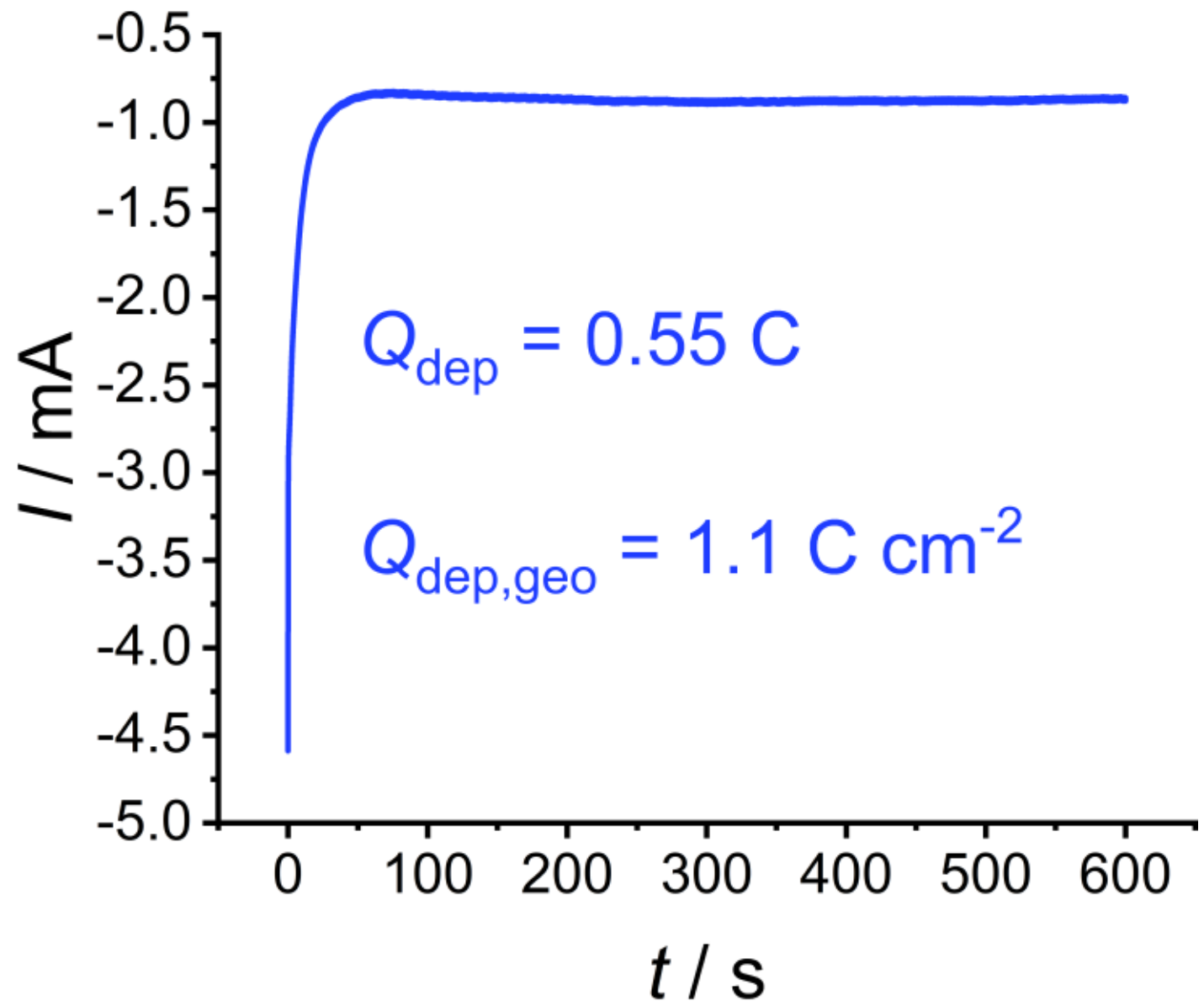

Figure S3. Chronoamperometric deposition curve at -0.79 V vs. Ag|AgCl|3 M KCl on Au-modified carbon paper.

1.4 *Operando* and *Ex situ* X-ray Absorption Spectroscopy Experiments

Operando X-ray absorption spectroscopy (XAS) experiments were conducted in a three-electrode Teflon cell with a volume of ca. 30 mL similar to the cell used and developed by Feng et al.[3] The geometric area of the working electrode exposed to the electrolyte is about 0.5 cm^2 and was used to normalize the current response in all electrochemical experiments.

First, the Au-modified carbon paper was electropolished in 0.5 M H_2SO_4 (see SI-1.2). Second, the electrodeposition of the pristine nickel selenide was performed according to SI-1.3, and third, the electrolyte was changed to 100 mM KOH (Sigma-Aldrich, semi-conductor grade) + 1 mM $Fe(NO_3)_3$ (Sigma-Aldrich). The cell was accurately positioned in the beamline by checking the fluorescence signal at the Ni K edge. We intentionally supplemented the electrolyte with a defined concentration of iron ions to suppress the influence of unintentional contamination by iron ions, which are known to affect the catalytic activity of nickel oxide/oxyhydroxide-based OER electrocatalysts.[4] The procedure for producing the alkaline working solution was described elsewhere.[5]

Electrochemical OER investigations during XAS measurements were conducted using a Gamry interface 1010 E potentiostat with a commercial Hg|HgO|0.1 M KOH RE (ALS, RE-61AP) and a graphite rod CE (d = 6 mm). The following script, containing open circuit potentiometry (OCP), electrochemical impedance spectroscopy (EIS), cyclic voltammetry (CV), linear sweep

voltammetry (LSV), and chronoamperometry (CA) was applied using Gamry Framework (Version 7.8.2):

1. OCP for 60 s
2. EIS at OCP; 10 kHz – 0.05 Hz; 5 mV (rms)
3. CV: 0.0 – 0.8 V vs. Hg|HgO|0.1 M KOH, v = 2 mV s^{-1}, 3 cycles
4. OCP for ca. 5200 s
5. EIS at OCP; 10 kHz – 0.05 Hz; 5 mV (rms)
6. LSV from OCP to 0.450 V vs. Hg|HgO|0.1 M KOH, v = 2 mV s^{-1}
7. EIS at 0.450 V vs. Hg|HgO|0.1 M KOH; 10 kHz – 0.05 Hz; 5 mV (rms)
8. CA at 0.450 V vs. Hg|HgO|0.1 M KOH for ca. 5200 s
9. EIS at 0.450 V vs. Hg|HgO|0.1 M KOH; 10 kHz – 0.05 Hz; 5 mV (rms)
10. LSV from 0.450 to 0.525 V vs. Hg|HgO|0.1 M KOH, v = 2 mV s^{-1}
11. EIS at 0.525 V vs. Hg|HgO|0.1 M KOH; 10 kHz – 0.05 Hz; 5 mV (rms)
12. CA at 0.525 V vs. Hg|HgO|0.1 M KOH for ca. 5200 s
13. EIS at 0.525 V vs. Hg|HgO|0.1 M KOH; 10 kHz – 0.05 Hz; 5 mV (rms)
14. LSV from 0.525 to 0.560 V vs. Hg|HgO|0.1 M KOH, v = 2 mV s^{-1}
15. EIS at 0.560 V vs. Hg|HgO|0.1 M KOH; 10 kHz – 0.05 Hz; 5 mV (rms)
16. CA at 0.560 V vs. Hg|HgO|0.1 M KOH for ca. 5200 s
17. EIS at 0.560 V vs. Hg|HgO|0.1 M KOH; 10 kHz – 0.05 Hz; 5 mV (rms)
18. LSV from 0.560 to 0.670 V vs. Hg|HgO|0.1 M KOH, v = 2 mV s^{-1}
19. EIS at 0.670 V vs. Hg|HgO|0.1 M KOH; 10 kHz – 0.05 Hz; 5 mV (rms)
20. CA at 0.670 V vs. Hg|HgO|0.1 M KOH for ca. 5200 s
21. EIS at 0.670 V vs. Hg|HgO|0.1 M KOH); 10 kHz – 0.05 Hz; 5 mV (rms)
22. LSV from 0.670 to 0.700 V vs. Hg|HgO|0.1 M KOH, v = 2 mV s^{-1}
23. EIS at 0.700 V vs. Hg|HgO|0.1 M KOH; 10 kHz – 0.05 Hz; 5 mV (rms)
24. CA at 0.700 V vs. Hg|HgO|0.1 M KOH for ca. 5200 s
25. EIS at 0.700 V vs. Hg|HgO|0.1 M KOH; 10 kHz – 0.05 Hz; 5 mV (rms)
26. LSV from 0.700 to 0.730 V vs. Hg|HgO|0.1 M KOH, v = 2 mV s^{-1}
27. EIS at 0.730 V vs. Hg|HgO|0.1 M KOH; 10 kHz – 0.05 Hz; 5 mV (rms)
28. CA at 0.730 V vs. Hg|HgO|0.1 M KOH for ca. 5200 s
29. EIS at 0.730 V vs. Hg|HgO|0.1 M KOH; 10 kHz – 0.05 Hz; 5 mV (rms)
30. LSV from 0.730 to 0.000 V vs. Hg|HgO|0.1 M KOH, v = 2 mV s^{-1}
31. CV: 0.0 – 0.8 V vs. Hg|HgO|0.1 M KOH, v = 2 mV s^{-1}, 1 cycle
32. OCP for ca. 5200 s
33. EIS at OCP; 10 kHz – 0.05 Hz; 5 mV (rms)

At each CA step, at least three XAS scans were recorded. The potentials applied in the CA steps (script experiments 4, 8, 12, 16, 20, 24, and 28) were selected according to the shape of the third CV cycle of the activation CV (experiment 3), which is depicted in Figure S4. The purpose

of the activation CV (experiment 3) is to initiate the transformation of the pre-catalytic nickel selenide to an oxide/oxyhydroxide species.

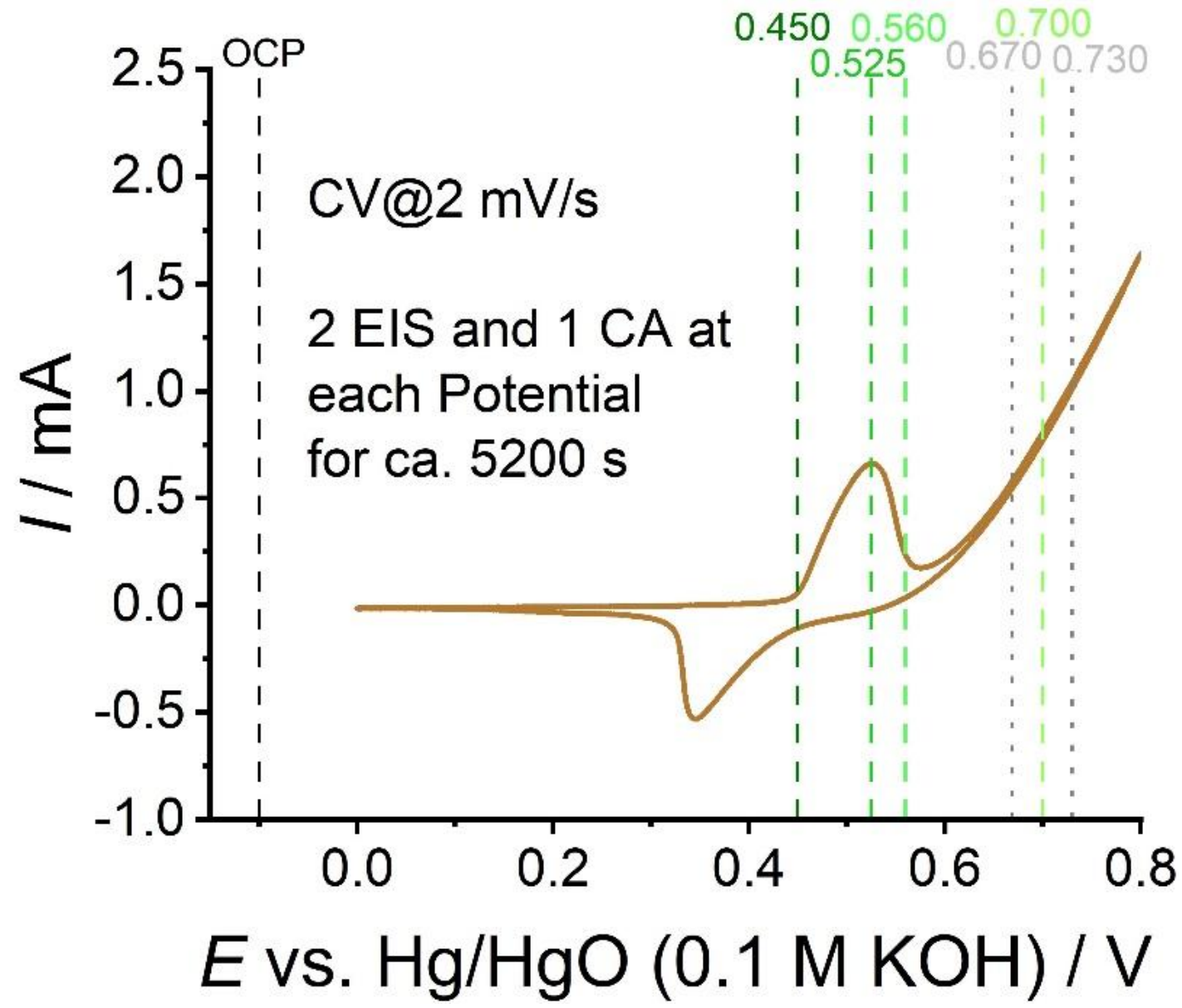

Figure S4. Third CV cycle of the activation CV (experiment 3 of the EC-script). The potentials at which the XAS scans were recorded are indicated by dashed lines.

The potential of 0.450 V vs. Hg|HgO|0.1 M KOH was selected since it is located at the foot of the nickel oxidation peak. The peak potential of the nickel oxidation peak occurs at 0.525 V vs. Hg|HgO|0.1 M KOH and 0.560 V vs. Hg|HgO|0.1 M KOH, the nickel oxidation is almost completed, but the OER has not commenced and, thus, cannot contribute to the current of the spectroscopic signals. Catalytic OER proceeds at the three potentials 0.670 V, 0.700 V, and 0.730 V vs. Hg|HgO|0.1 M KOH. We conducted EIS measurements before and after every CA step to extract information about the transformational and pseudo-capacitive processes of the catalyst. For simplicity, only the data at 0.700 V vs. Hg|HgO|0.1 M KOH are discussed for the OER potential range in the main manuscript since the data recorded at 0.670 V and 0.730 V vs. Hg|HgO|0.1 M KOH show no significant differences to the ones at 0.700 V.

For data presentation, the potential values recorded against Hg|HgO|0.1 M KOH were converted to the reversible hydrogen electrode (RHE) scale according to equation (S1):[6]

$$E_{\mathrm{RHE}} = E_{(\mathrm{Hg|HgO|0.1\ M\ KOH})} + (0.059 \cdot pH) + 0.164\ \mathrm{V} \quad \text{(S1)}$$

Operando X-ray absorption near edge structure (XANES) and extended X-ray absorption fine structure (EXAFS) experiments were conducted at beamline 5BM-D of DND-CAT, Advanced Photon Source (APS), Argonne National Laboratory (ANL) by the Feng group. All *operando* data were collected in fluorescence mode at the potentials applied during each chronoamperometric experiment described above. The X-ray beam was calibrated using a Ni metal foil. Data reduction and data analysis were performed with the Athena software packages (available on GitHub, version

0.9.26) and the included Artemis software package was used to perform model fitting for EXAFS spectra.[7] The pre-edge background was linearly fitted and subtracted. The post-edge background was determined using a cubic-spline-fit procedure and then subtracted and normalized. The k-range for the Fourier Transform was kept from 3 to 11.5 for all datasets and presented in k-weight of 2. The amplitude reduction factor (S_0^2) values were obtained from model-fitting EXAFS spectra of NiSe and NiOOH standards. Once S_0^2 was established, the EXAFS spectra of the catalyst materials were analyzed using these calibrated amplitudes. The EXAFS fitting was carried out using k-weights of 1, 2, and 3 to ensure robustness of the structural parameters, with the results for k-weight = 2 presented in the manuscript.

Ex-situ X-ray absorption near structure (XANES) and extended X-ray absorption fine structure (EXAFS) at Ni K-edge experiments were performed at beamline 20-BM-B of the Advanced Photon Source (APS) of Argonne National Laboratory (ANL). A Vertox ME4 silicon drift diode detector was used to collect the Ni K fluorescence signal while the Si (111) monochromator scanned the incident X-ray photon energy through the Ni K absorption edge. A Ni foil was used as a standard to calibrate the energy. Ni K-edge XANES and EXAFS data were measured in fluorescence mode. All XANES and EXAFS data analyses were performed with the Athena software package (available on GitHub in Athena software packages, version 0.9.26) following the same procedure as in *operando* XAS.

The β-NiOOH, which was used as a reference material for both *ex situ* and *operando* XAS data, was synthesized by our collaborator from a previous study,[8] with the phase confirmed.

1.5 *Ex situ* X-ray Photoelectron Spectroscopy

XPS measurements were performed using a Thermo Fisher ESCALAB 250 Xi instrument in UHV conditions ($2 \cdot 10^{-7}$ mbar) with a monochromatic Al Kα X-ray source (λ = 1486.6 eV; spotsize 650 or 900 µm, anode operating at 14 kV and 90 W). A flood gun was applied to mitigate the effects of charge accumulation. All samples were grounded to the sample holder, and the take-off angle was 90°. Survey spectra were collected at a pass energy of 100 eV, and high-resolution spectra of the different regions at a pass energy of 10 eV. Data analysis was conducted using the software CasaXPS version 2.3.25PR1.0.[9] The Se 3d spectra were analyzed using Avantage version 5.9931. The binding energy scale of samples deposited on Au-glass was charge-corrected by setting the C 1s of C-C, *C*-H of adventitious carbon to 284.8 eV. For samples deposited on Au-carbon paper, the C 1s peak of graphitic carbon was set to 284.5 eV.[10] A Shirley background was used for background correction. For the Se 3d spectra, the option 'Smart background' of the Avantage software was used which is a combination of a Shirley background and a second order polynom. The lineshapes of the peaks were described in CasaXPS by a Gaussian-Lorentzian GL(*m*) product formula, where *m* denotes the mixing ratio of Lorentzian and Gaussian, with *m* = 100 being a purely Lorentzian line shape. The lineshape of the graphitic C 1s peak in CasaXPS was defined using a Lorentzian asymmetric lineshape LA(α,β,*m*) where *m* is a value between 0 and 499 describing the width of the Gaussian convoluted with the Lorentzian curve. The spread of the Lorentzian tail is

defined by the parameters α (higher binding energy) and β (lower binding energy) with higher values meaning a lower spread. For adventitious carbon, the lineshape GL(30) was used, and for graphitic carbon LA(1.25,2,1). In Avantage, the lineshape of the Se 3d was described by symmetric Gaussian/Lorentzian lineshape (SymGL). The ratio between Gaussian and Lorentzian contributions was not constrained and was approximately 0.4 for the analyzed spectra.

To identify the chemical nature of nickel in the nickel selenide films, an approach based on the work of Biesinger et al. was utilized to fit the Ni $2p_{3/2}$.[11] The peak models for NiO, $Ni(OH)_2$, γ-NiOOH, and β-NiOOH of Biesinger et al. were used to fit the spectra by constraining the peak components in their relative area, relative peak position, and relative FWHM to the first component (see Table S5). Further, reference spectra from our previous work were used to construct empirical peak models for the Ni $2p_{3/2}$ of NiSe and Ni_3Se_2 (Figure S9).[5] To account for the variation in adventitious carbon referencing, the position of the spectral components was allowed to vary by ±0.2 eV.[12] The FWHM of the peak components was allowed to increase by up to 0.2 eV from the literature values. Monte Carlo simulations in CasaXPS were used to estimate the accuracy of the fits and the software's internal error calculation tool to obtain approximate standard deviations for the species quantification.

Due to the similarity of the Ni $2p_{3/2}$ reference spectra of NiSe and Ni_3Se_2 (Figure S9), the Se 3d region of the samples was used to identify the dominant nickel selenide species based on the analysis of the reference samples (Figure S10).

To investigate the incorporation of iron in the nickel selenide films during OER, XPS spectra of the Ni 2p and Fe 2p regions were recorded. The detection of small amounts of iron in a nickel-rich matrix is not feasible with an Al K_α X-ray source due to the overlap of the Fe 2p with the Ni LMM Auger signals.[13] Hence, a Mg K_α X-ray source (λ = 1253.6 eV, 400 W) was used to avoid the overlap (pass energy 10 eV). Furthermore, the sample was sputtered with Ar^+ (3 keV, etch area 1.5×1.5 mm^2, 15 cycles with 10 s sputter time each) to remove the topmost layer, and another set of spectra was acquired to assess the depth distribution of Fe. For samples deposited on the PTFE-treated carbon paper, spectral overlap of the F KL_1L_{23} signal has to be considered for the fit of the Ni $2p_{3/2}$ spectra. Spectra of the blank substrate were recorded to obtain the position, FWHM, and ratio of the F KLL signal (Figure S13, Table S8). Then, this information was used to estimate the contribution of the F KL_1L_{23} emission under the Ni $2p_{3/2}$ signal by integrating the F $KL_{23}L_{23}$ signal that does not overlap.

The Au-coated glass slides (A = 0.283 cm^2) were fabricated by thermal evaporation. Before the evaporation of a 200 nm thick gold layer, a titanium adhesion layer of about 8 nm thickness was evaporated on the glass slide. Electrodeposition of nickel selenide films was conducted as described in SI-1.3.

1.6 E*x situ* XRD Experiments

Ex situ XRD was conducted on the pristine nickel selenide deposit on Au-modified carbon paper. Crystallographic phase analysis was performed, conducting X-ray diffraction (XRD) using Bruker D8 Discover with a Vantec-500 2D-detector in Bragg–Brentano geometry and a Cu Kα X-ray source (generator settings 36 mA; 36 kV). To avoid Au-substrate peaks, measurements were

performed in θ–2θ mode, where the X-ray source is fixed in position, whereas the sample rotates with θ °/min and the 2D-detector with 2θ °/min.

Powder XRD (PXRD) characterization and the synthesis route of hexagonal NiSe (phase pure) and rhombohedral Ni_3Se_2 (main phase, hexagonal NiSe minor phase) reference samples used for comparison to electrodeposited films are described elsewhere.[5]

1.7 TEM Experiments

(S)TEM measurements were performed at 200kV on a Jeol JEM-ARM200F NEOARM instrument equipped with a Cs corrector for the condenser system. The EDX measurements were performed in STEM mode using a Jeol EDX ex-230**BU Ex-37001 system. The EDX data was treated using multivariate analysis.[14] The samples were prepared by scratching the material from the Au-modified carbon paper substrate wetted with a droplet of ca. 0.5 mL of MilliQ water and subsequently depositing the liquid with the material in dispersion into a Cu/C TEM grid.

1.8 DEMS Experiments

DEMS experiments with ^{18}O-labeled electrolytes were conducted in a dual thin-layer cell described elsewhere[15] on a in-house-built DEMS setup[16] that was constructed following the design principles outlined by Wolter and Heitbaum.[17]

The experimental sequence of our Isotope exchange experiments was as follows: first, 20 CVs were recorded in an electrolyte (1.0 M KOH) featuring 5% $H_2{}^{18}O$. If oxygen evolution proceeds via the lattice oxygen exchange mechanism, this leads to the accumulation of ^{18}O in the anion lattice of the NiO(OH)-top layer. Second, the potential is held at a value where no oxygen evolution proceeds (i.e., 0.4 V vs Ag|AgCl|3.5 M KCl). During the potential hold, the electrolyte is exchanged from the $H_2{}^{18}O$-rich electrolyte to an electrolyte that features $H_2{}^{18}O$ with natural abundance. Third, the potential sweep is resumed, and 10 CVs are collected in the $H_2{}^{18}O$ lean electrolyte.

In this experimental sequence, we avoid the potential window in which the transition between and $Ni(OH)_2$ and NiOOH takes place, and that is associated with the large pseudocapacitive processes shown in the black curve of Figure S5. Instead, the lower potential limit was kept at 0.4 V (vs Ag|AgCl|3.5 M KCl) as shown in the red curve of Figure S5. This was done to prevent any isotope exchange with the electrolyte due to a phase transition and to avoid the incorporation of lattice water into the catalyst. The latter may form a reservoir of $H_2{}^{18}O$ that is not removed during the electrolyte exchange but may act as a source of ^{18}O during the oxygen evolution reaction. This would lead to elevated levels of the mol fraction of $^{16}O^{18}O$ ($\chi(^{16}O^{18}O)$) in the evolved oxygen and would give a false positive indication for oxygen evolution via the lattice oxygen exchange mechanism (*vide supra*).

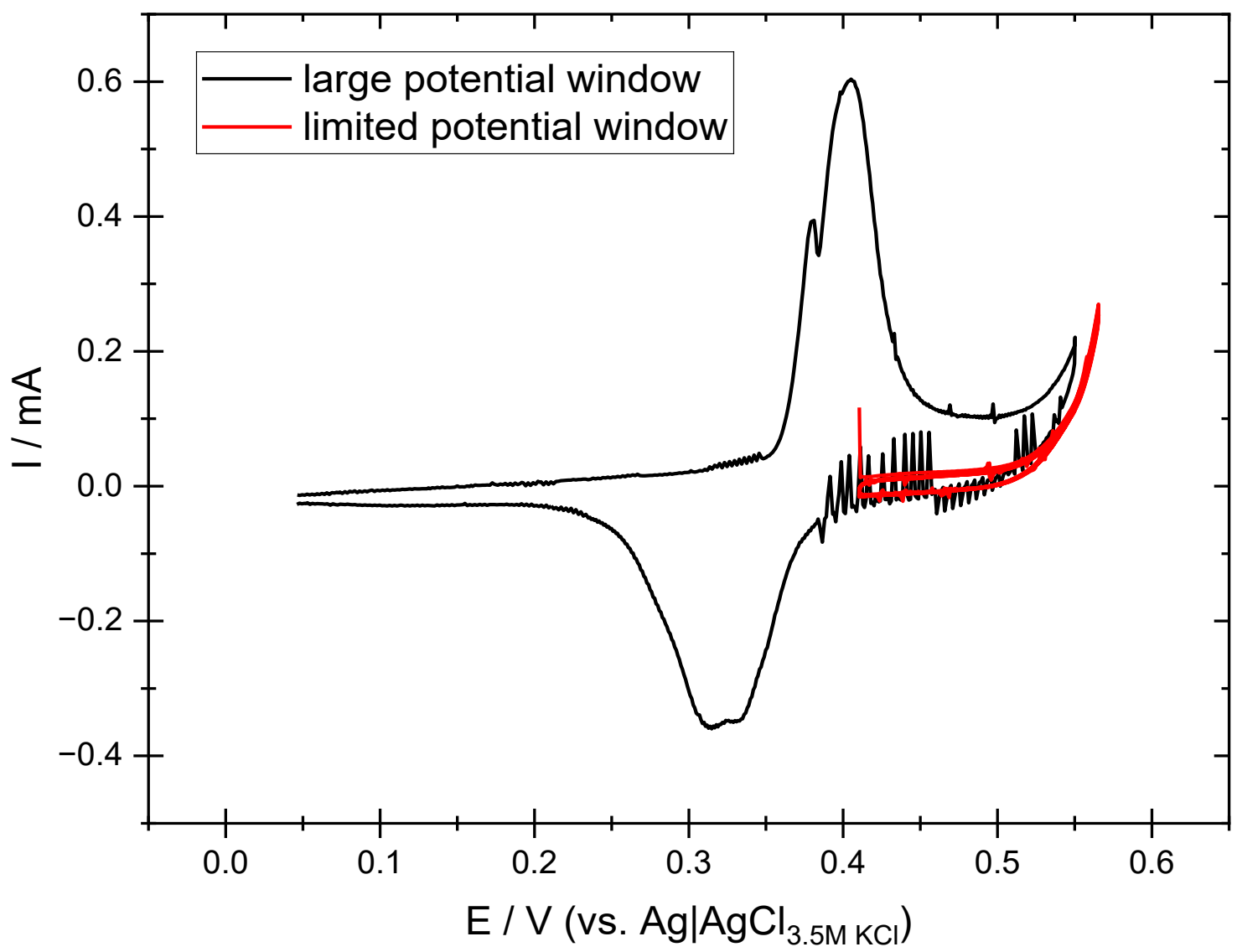

Figure S5. CV of activated NiSe-film deposited on Au (A_{geo} = 0.283 cm^2) in a dual thin-layer cell. Electrolyte: 1.0 M KOH in $H_2{}^{16}O$; Flow rate: 5 µL s^{-1}; sweep rate: 20 mV s^{-1} (black) and 5 mV s^{-1} (red).

1.9 RDE Experiments

Rotating disk electrode (RDE) experiments were conducted using an RRDE-3A setup by ALS with an Au-RDE WE (011171, ALS; *d* = 3 mm) and either a Gamry Reference 600 or a BioLogic VSP-3000 potentiostat. The used CE and RE are described in SI-1.1. The Au-RDE was electropolished in a series of chronoamperometric (CA) and cyclovoltammetric (CV) experiments in 0.5 M H_2SO_4. First, a 30 s CA at 3 V vs. Ag|AgCl|3 M KCl was conducted to fully oxidize the gold surface, which turned into an orange color (indicating full oxidation to gold oxide of the uppermost gold layers). The gold oxide was removed chemically by dipping the Au-RDE in concentrated HCl for a few seconds. This step was repeated once. Afterward, a CV experiment of 20 cycles at a scan rate of 0.1 V s^{-1} in a potential window of -0.1 V to 1.8 V vs. Ag|AgCl|3 M KCl (initial/final potential 0.2 V) was performed, followed by another CA at 3 V vs. Ag|AgCl|3 M KCl for 30 s and removal of the gold oxide by dipping in concentrated HCl.

Before the electrodeposition of nickel selenide, another 20 CV cycles in a potential window of −0.1 V to 1.45 V vs. Ag|AgCl|3 M KCl (initial/final potential 0.2 V) at a scan rate of 0.1 V s^{-1} in 0.5 M H_2SO_4 were applied, similar to the procedure on Au-modified carbon paper (see SI-1.2). Roughness factors, determined by dividing A_m by the geometric area A_{geo}, were between 1.1 and 1.5 in all conducted experiments.

Electrodeposition of pristine nickel selenide films was performed according to SI-1.3. For comparison of the EIS experiments with those conducted in the XAS cell, the same script of electrochemical measurements was applied (see SI-1.4). Since the potential of the nickel oxidation peak on the Au-RDE substrate was less intense and shifted cathodically compared to the experiment on Au-modified carbon paper (see Figure S6), the potentials in the experiments 10 to 18 of the script were adjusted to 0.500 V (in case of XAS cell 0.525 V for the peak potential of

nickel oxidation) and 0.540 V (in case of XAS cell 0.560 V), respectively. All mentioned potentials were measured against Hg|HgO|0.1 M KOH.

The Tafel slope of the active catalyst was determined from experiments 1 to 5 from the electrochemical script in SI-1.4, and a series of CA experiments starting at 0.56 V and finishing at 0.73 V vs. Hg|HgO|0.1 M KOH with a potential step separation of 10 mV and a duration of 5 min per CA experiment. The current recorded after 5 min was taken as steady-state current to estimate the Tafel slope. There was a relaxation period of 1 min at OCP between two potential steps. The Tafel slope was estimated by a linear fit of the *IR*-corrected currents at each potential of the CA experiments. Electrodeposits on Au-RDE as well as Au-modified carbon paper were analysed by the same procedure.

To remove electrodeposited catalyst films, a CA at 1 V vs. Ag|AgCl|3 M KCl was applied for 30 s in 0.5 M H_2SO_4 followed by the entire electrochemical cleaning procedure in renewed 0.5 M H_2SO_4 solution as described above.

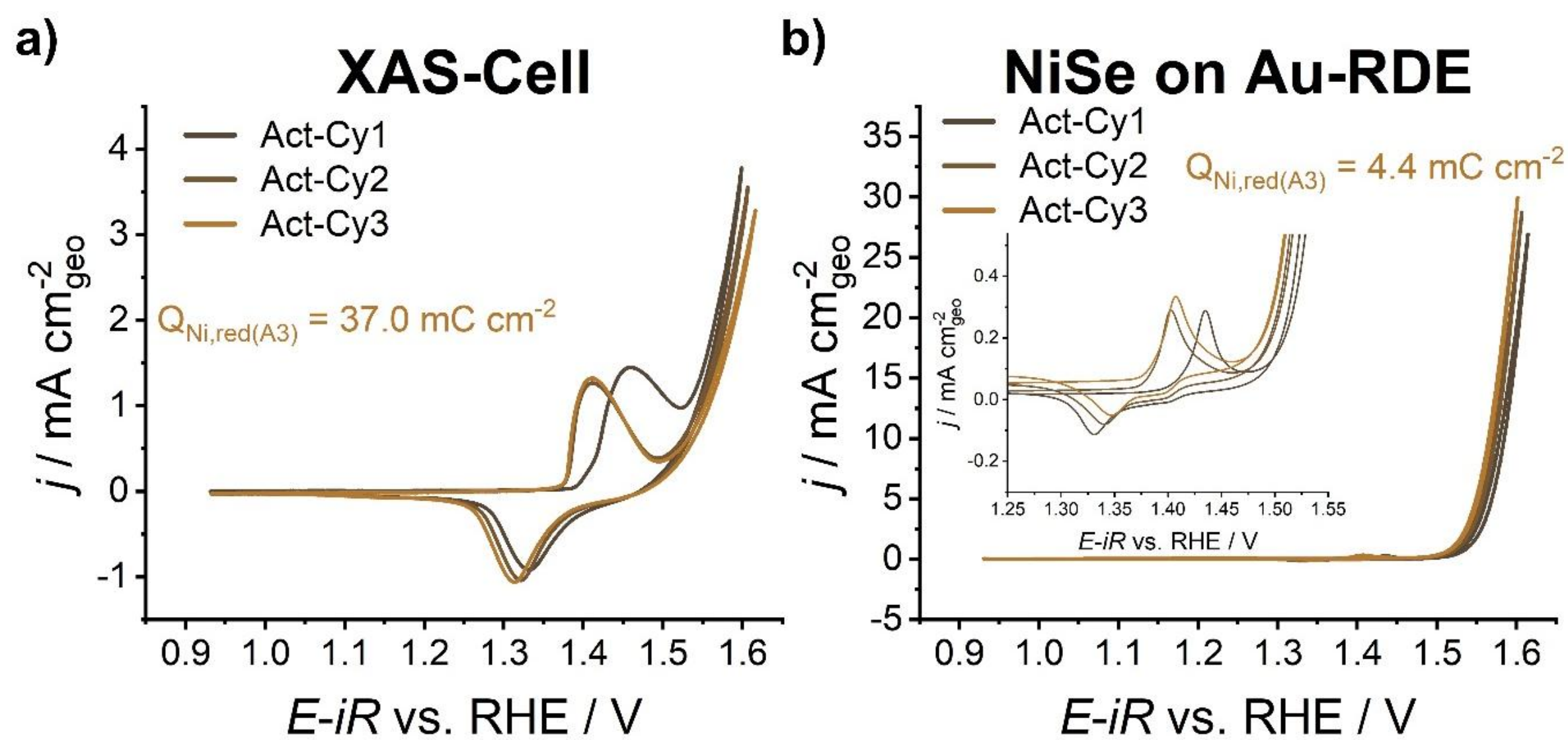

Figure S6. Three CV cycles of the activation CV (experiment 3 of EC-script) of the electrodeposited catalyst film a) in the operando XAS cell and b) on an Au-RDE.

1.10 *Operando* Raman Spectroscopy

Operando Raman spectroscopy was conducted using a Raman microscope (Alpha-300R confocal Raman microscope, WITec GmbH) using a 63× water immersion objective (Zeiss W Plan Apochromat 63×/1.0 VIS-IR), protected with a 25-µm-thick foil of FEP (fluorethylenepropylene), to prevent any damage or contamination during the different experiments. A laser source of 532 nm was used with a power of 1 mW, and an integration time of 1s was set for all the experiments. The Raman microscope was coupled to the Reference 600 Gamry potentiostat working in a three-electrode configuration. The experiments were performed in an in-house 3D-printed cell of polyvinylidene fluoride (PVDF). All Raman spectra were pre-processed following a standard

workflow: cosmic rays were removed, a Savitzky-Golay filter was applied, and the background was subtracted using the shape model implemented in the Project SIX 6.1.7.131 software package (WITec). In addition, for Figure S37 an extra background subtraction was performed to exclude the contribution from water.

Au-modified carbon paper with electrodeposited nickel selenide (see SI-1.2) was used as WE with an electrolyte-exposed geometric area of ca. 0.1963 cm^2 (d = 5 mm). A carbon rod and an in-house-made Ag|AgCl|3 M KCl were used as CE and RE, respectively. Raman spectra at OCP and at the foot of the nickel oxidation wave were recorded after measurements 1 to 5 of the electrochemical script described in SI-1.4in 0.1 M KOH + 1 mM $Fe(NO_3)_3$. Figure S7a depicts the three CV cycles of the activation period (see experiment 3 in the EC script described in SI-1.4). All potentials were converted to the RHE scale according to Equation (S2).[6]

$$E_{\mathrm{RHE}}=E_{(\mathrm{Ag|AgCl|3MKCl})}+(0.059 \cdot pH)+0.207\ \mathrm{V} \qquad \text{(S2)}$$

The charge of the nickel reduction peak in the experiments in the *operando* Raman cell is more than six times lower compared to the experiments in the *operando* XAS cell (Figure S6a). Furthermore, the second R-C element in the first EIS spectrum (recorded before the CA at the foot of the nickel oxidation wave in XAS experiments, see Figure 4b in main manuscript and FiguresFigure S30b andFigure S32b) is absent, and its shape is similar to the second EIS spectrum (recorded after the CA at the foot of the nickel oxidation wave, see Figure S7b). Hence, we conclude that the appearance of the second R-C element in the first EIS spectrum at 1.37 V vs. RHE in the *operando* XAS cell is only observable with a sufficiently high amount of electrochemically active nickel and with this intensity (charge extracted from) the nickel redox wave.

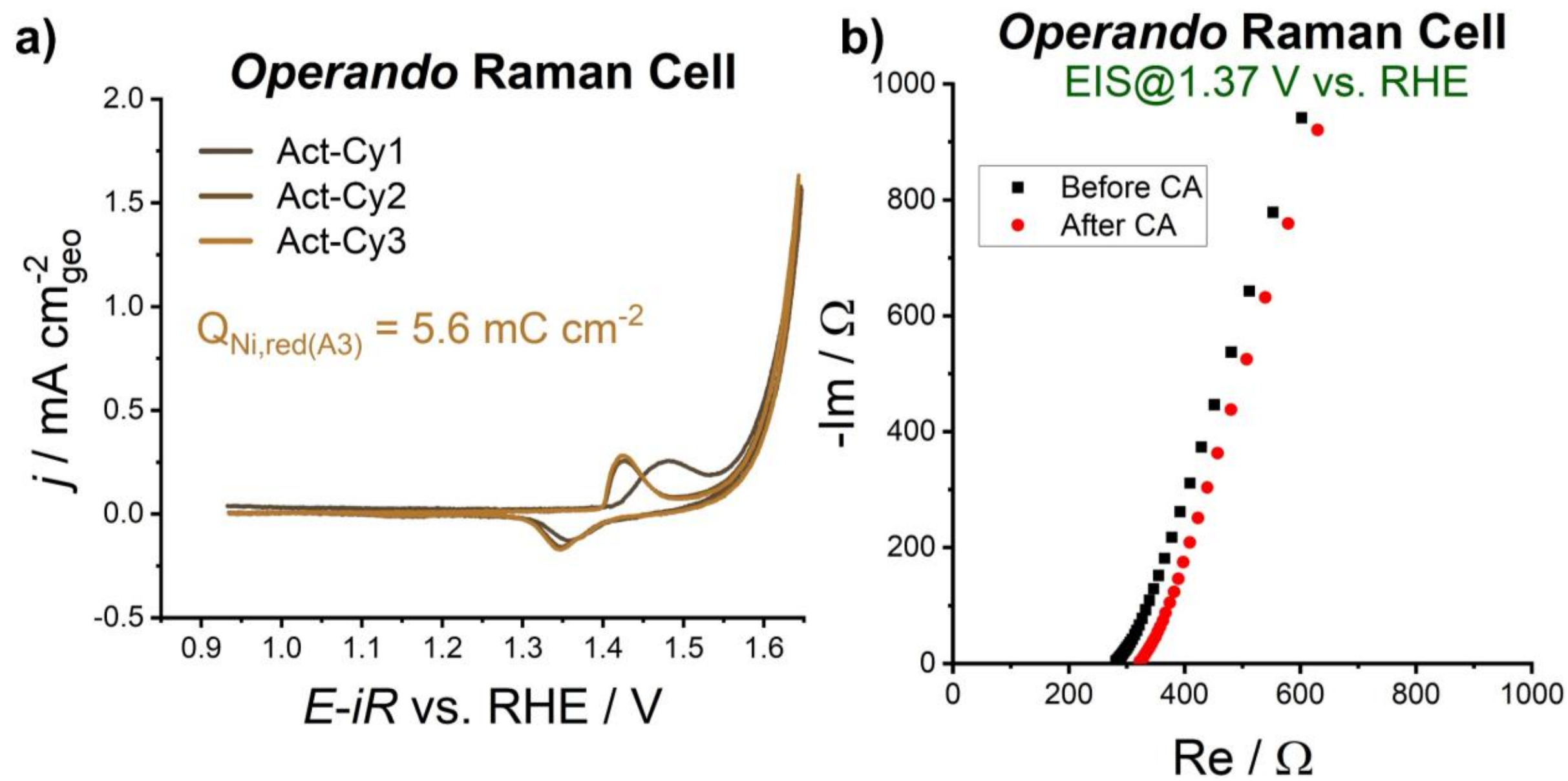

Figure S7. a) Three CV cycles of the activation CV (experiment 3 of EC-script) of the electrodeposited catalyst film in the *operando* Raman cell; b) EIS spectra at 1.37 V vs. RHE before and after the CA at 1.37 V vs. RHE.

1.11 *Operando* and *Ex situ* ICP-MS Investigations

The scanning flow cell (SFC) design used during the *operando* ICP-MS experiments was described before.[18–20] The cell was made of Makrolon and had channels with a diameter of 1 mm. For all *operando* ICP-MS experiments, a Gamry Reference 600 potentiostat was used. Au-modified carbon paper with electrodeposited nickel selenides (area exposed to electrolyte with ca. $d = 1$ mm) according to SI-1.3 was used as a WE together with a Pt wire (0.5 mm, 99.997 %, Alfa Aesar) as CE and a commercial Ag|AgCl|3 M KCl as RE (Metrohm). All potentials were converted to the RHE scale using Equation (S2) (see SI-1.8).

The electrolyte was pumped with a flow rate of approximately 200 µL min^{-1} through the cell and subsequently introduced into an inductively coupled plasma-mass spectrometer (ICP-MS, NexION 300X, Perkin Elmer) for time-resolved analysis of the concentration of dissolved ions (^{56}Fe, ^{58}Ni, ^{60}Ni, ^{78}Se, ^{80}Se). Methane was used as a reaction gas to remove interference on the masses of Fe and Se at a flow rate of 0.7 mL min^{-1}. The detected intensities were analyzed with respect to an internal standard for compensation of physical interferences. For this purpose, ^{89}Y was used in 1% HNO_3 (prepared from suprapur HNO_3, 65%, Merck) with a 10 µg L^{-1} concentration. The acid was used to acidify the electrolyte before entering the ICP-MS. The internal standard solution was added to the electrolyte via a Y-connector behind the SFC. ICP-MS calibration was performed every day before the measurements and was used to convert the detected intensities to the concentration of the dissolved ions in the electrolyte. *Operando* ICP-MS measurements were conducted in 0.01 M KOH (Merck, suprapur, 99,995%, KOH*H_2O).

For the *ex situ* ICP-MS characterization, the electrodeposited films on Au-modified carbon paper in pristine and after OER conditions were dissolved in 1% HNO_3 (suprapur HNO_3, 65%,

Merck). The analysis of the solutions was also carried out with an ICP-MS (NexION 300X, Perkin Elmer) using ^{89}Y as the internal standard for all elements with a concentration of 5 µg L^{-1}. The quantification limits were < 0.5 µg L^{-1} for ^{58}Ni and ^{56}Fe and < 0.4 µg L^{-1} for ^{80}Se.

1.12 SEM/EDX Investigations

Scanning electron microscopy (SEM) investigations were performed using a high-resolution scanning electron microscope JSM-IT800 (JEOL) at 10 kV acceleration voltage. An Ultim Max Silicon Drift Detector (SDD) by Oxford Instruments at 10 kV was used as a primary detector to monitor energy dispersive X-ray (EDX) spectra. Data processing and analyses of the EDX spectra were performed using AZtecLive (Oxford Instruments).

1.13 Data Processing

Analysis of impedance data was performed using RelaxIS 3 (rhd instruments). A complementary analysis was done with an open-source code with a graphical user interface developed by Jean-Luc Dellis.[21] For data processing, a Z-HIT algorithm was used. For further data analysis and construction of figures, OriginPro 2021b was used. All electrochemical measurements were manually iR-corrected with 85% of the R_s value extracted from EIS measurements at OCP.

1.14 Visualisation of Crystal Structures

The crystal structures of NiSe, β-$Ni(OH)_2$, β-NiOOH, and NiO_2 were generated using VESTA from JP-minerals. The cif-data-files corresponding to each species were downloaded from the open-source database, The Materials Project, powered by the Berkeley Lab (Creative Commons Attribution 4.0 International License).

2. Supplementary Experimental Data

2.1 XPS Data

2.1.1 Iron Content in NiSe Material after OER in Fe-Supplemented Electrolyte Solution

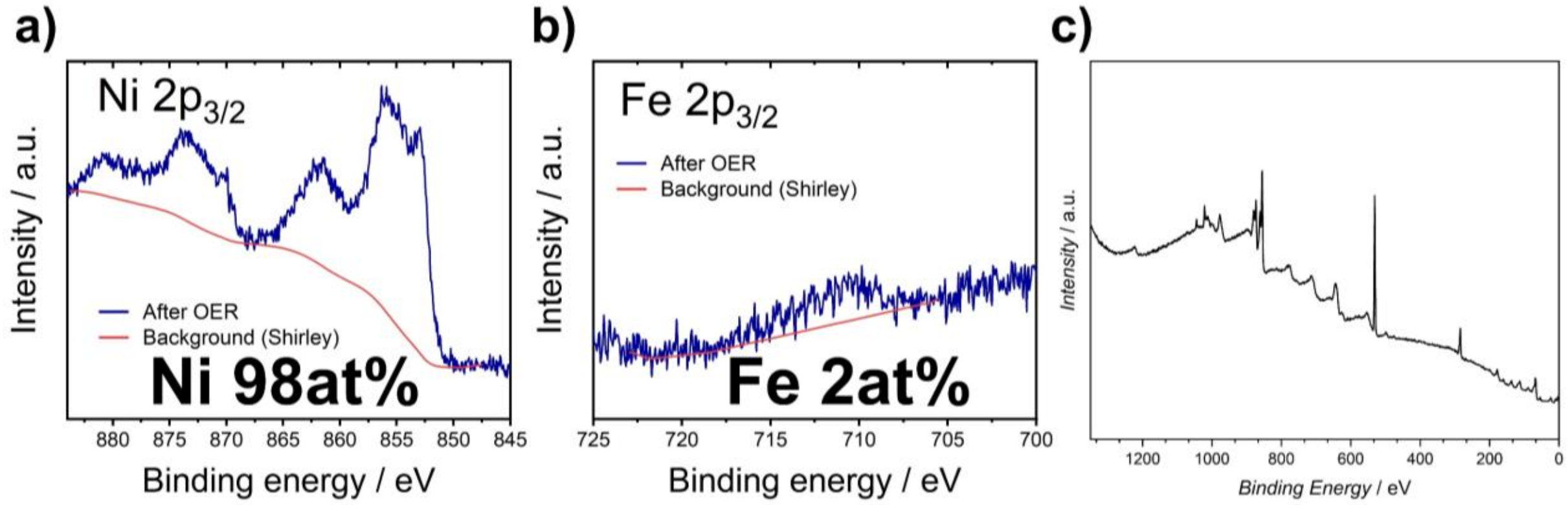

Figure S8. a) Ni $2p_{3/2}$ and b) Fe $2p_{3/2}$ *ex situ* XPS spectra of activated NiSe after OER in 100 mM KOH + 1 mM $Fe(NO_3)_3$ and Ar^+-sputtering recorded using a Mg K_α source. The sample was NiSe deposited on Au-coated glass slides, activated and used for OER followed by Ar-sputtering as described in SI-1.5, similar to an earlier study of Trotochaud et al.[13] The atomic ratio extracted from the integration of the Ni $2p_{3/2}$ and Fe $2p_{3/2}$ signals is 98at%:2at%; c) Survey spectrum of the sample after OER recorded using Al K_α.

2.1.2 Reference spectra of NiSe and Ni_3Se_2 Samples and Peak Models

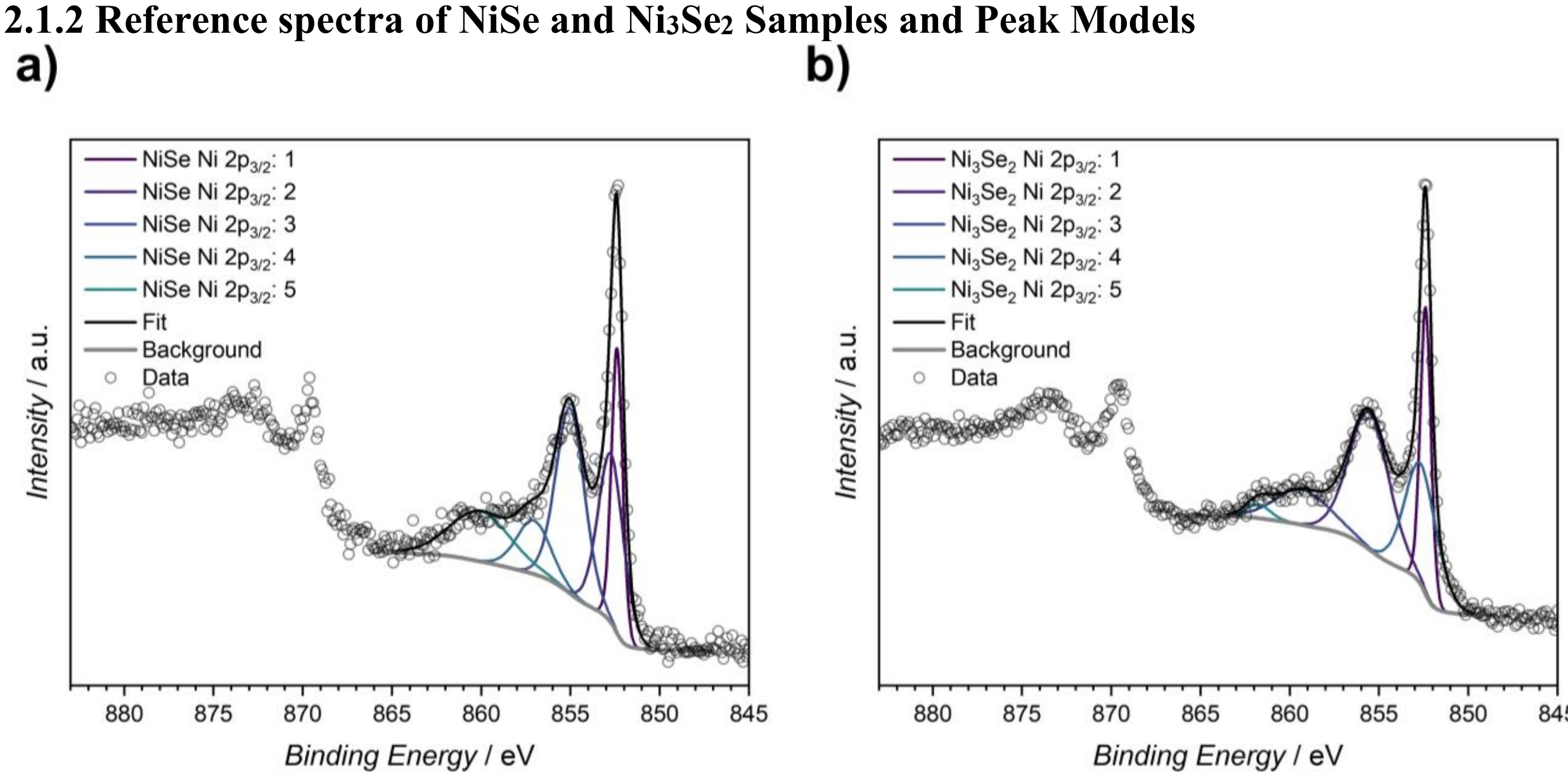

Figure S9. Ni 2p spectra of the (a) NiSe and (b) Ni_3Se_2 reference samples with peak models for the Ni $2p_{3/2}$.

For the fitting of the nickel selenide modified electrodes, empirical peak models were constructed to model the shape of the Ni $2p_{3/2}$ spectra. The models were then employed for fitting of the samples as described above.

Table S1. Peak model for the Ni $2p_{3/2}$ of NiSe constructed from the spectrum of reference powder material with peak positions, FWHM, and area of the peaks.

Species	Peak	Line Shape	Position / eV	FWHM / eV	Area / a.u.
	1	GL(30)	852.4 *	0.73 *	None
	2	GL(30)	NiSe-(1) + 0.34	NiSe-(1) + 0.96	NiSe-(1) · 1.36024
NiSe	3	GL(30)	NiSe-(1) + 2.63	NiSe-(1) + 1.17	NiSe-(1) · 1.68040
	4	GL(30)	NiSe-(1) + 4.67	NiSe-(1) + 1.50	NiSe-(1) · 0.57238
	5	GL(30)	NiSe-(1) + 6.94	NiSe-(1) + 3.27	NiSe-(1) · 0.95434

**For the fitting, NiSe-(1) was constrained to 852.2 – 852.6 eV in position and 0.73 – 0.93 eV in FWHM.*

Table S2. Peak model for the Ni $2p_{3/2}$ of Ni_3Se_2 constructed from the spectrum of the reference material powder with positions, FWHM, and area of the peaks.

Species	Peak	Line Shape	Position / eV	FWHM / eV	Area / a.u.
	1	GL(30)	852.4 *	0.70 *	None
	2	GL(30)	Ni_3Se_2-(1) + 0.24	Ni_3Se_2-(1) + 1.43	Ni_3Se_2-(1) · 1.27558
Ni_3Se_2	3	GL(30)	Ni_3Se_2-(1) + 3.16	Ni_3Se_2-(1) + 1.91	Ni_3Se_2-(1) · 1.78035
	4	GL(30)	Ni_3Se_2-(1) + 6.88	Ni_3Se_2-(1) + 2.87	Ni_3Se_2-(1) · 0.62528
	5	GL(30)	Ni_3Se_2-(1) + 9.37	Ni_3Se_2-(1) + 0.89	Ni_3Se_2-(1) · 0.10869

**For the fitting, Ni_3Se_2-(1) was constrained to 852.2 – 852.6 eV in position and 0.70 – 0.90 eV in FWHM.*

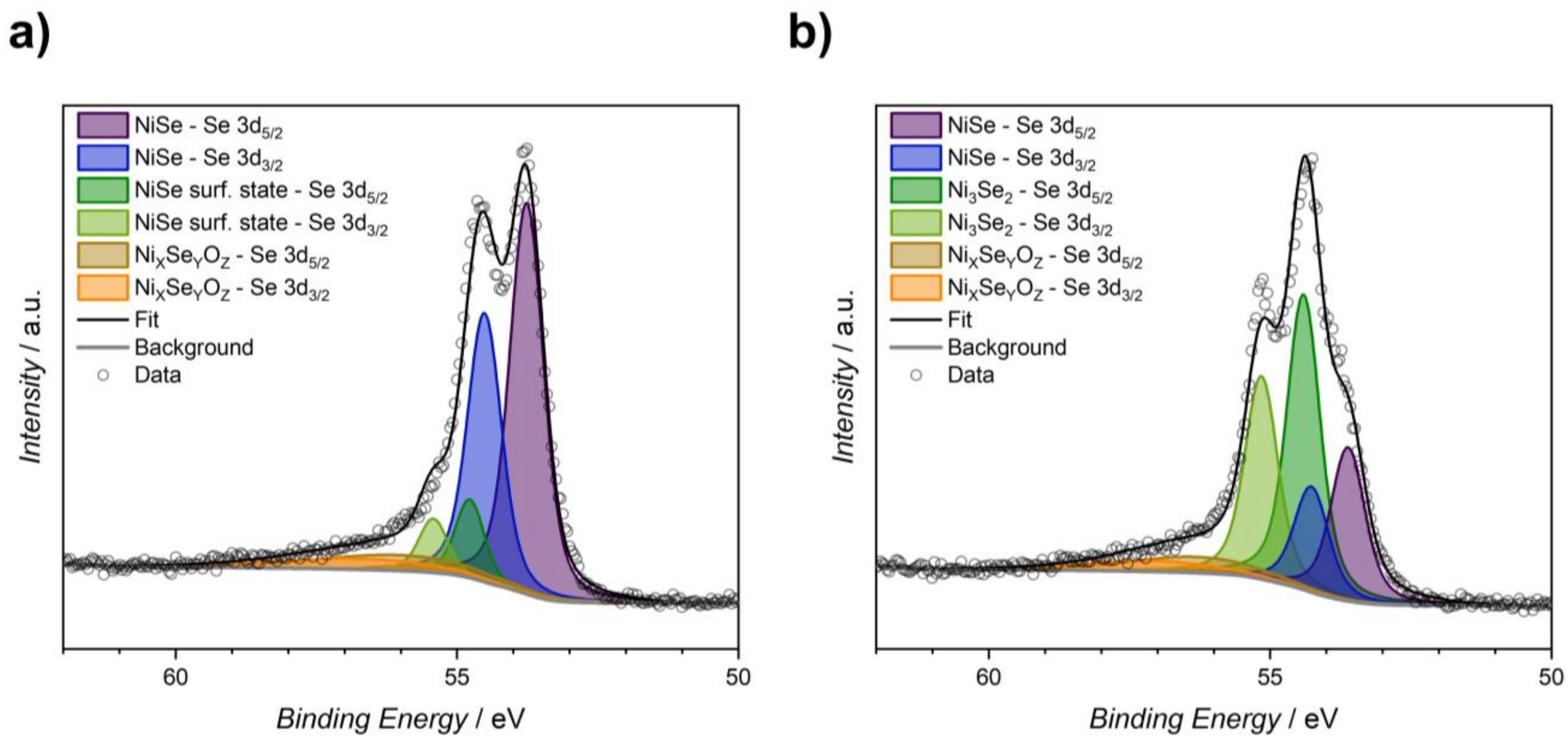

Figure S10. Se 3d spectra of the (a) NiSe and (b) Ni_3Se_2 reference samples.

The Se 3d reference spectra were fit by constraining the doublet splitting to 0.70 ± 0.04 eV and the Se 3d 3/2:5/2 height ratio to 0.688 while keeping constant the FWHM and ratio between Gaussian and Lorentzian for the lineshape. An analogous approach was used for the spectra of the electrodeposited nickel selenide films.

In the Se 3d spectrum of the NiSe reference sample, three Se 3d doublets were fit (Figure S10). The doublet with the Se $3d_{5/2}$ peak at 53.8 eV was assigned to NiSe. The doublet with the Se $3d_{5/2}$ peak at 54.8 eV can likely be attributed to surface states of nickel selenide, as previously reported for other metal chalcogenides.[22] The Se $3d_{5/2}$ peak at 56.0 eV exhibits a significantly increased FWHM and most likely originates from partially oxygenated nickel selenide-based species ($Ni_xSe_yO_z$). It should be mentioned that this species is observed at significantly lower binding energies than SeO_2 (59.4 eV) (Figure S19).

The most intense doublet in the Se 3d spectrum of the Ni_3Se_2 reference sample with Se $3d_{5/2}$ at 55.2 eV was assigned to the Ni_3Se_2 species. A second doublet at lower binding energy likely originates from a contamination of NiSe. As Ni_3Se_2 is formed from NiSe, phase-pure Ni_3Se_2 is often difficult to synthesize, and a surface contamination with NiSe seems plausible.[23,24] Similar to the NiSe reference sample, a doublet at high binding energy is observed that likely originates from oxygenated nickel selenide species.

Based on these reference measurements, we conclude that the presence of a dominant Se 3d doublet with Se $3d_{5/2}$ at ~53.8 eV is indicative of NiSe. For a sample with a significant NiSe contribution, a second doublet shifted to higher binding energy (Se $3d_{5/2}$: ~54.8 eV) is then either related to surface states of NiSe or possibly a contribution of Ni_3Se_2 (Se $3d_{5/2}$: ~54.4 eV). As the contribution of the doublet associated with NiSe surface states may change due to changed surface roughness and can thus not be constrained, a small Ni_3Se_2 contribution cannot be determined accurately. Within this study, the electrodeposited samples exhibited the dominant doublet

associated with NiSe and no drastically increased contribution of the Ni_3Se_2 / NiSe surface state doublet. Hence, we conclude that the sample surface is dominated by a NiSe species.

Table S3. Details of the fit of the Se 3d region of the NiSe reference sample. The peak positions, FWHM, and area of the peak components in % are listed together with concentrations of the species in %.

Species	Peak	Line Shape	Position / eV	FWHM / eV	Area / %	Species Conc. / %
NiSe	$3d_{5/2}$	SymGL	53.8	0.71	43.6	74
	$3d_{3/2}$	SymGL	54.5	0.71	30.0	
NiSe surface state	$3d_{5/2}$	SymGL	54.8	0.61	7.4	13
	$3d_{3/2}$	SymGL	55.4	0.61	5.1	
$Ni_XSe_YO_Z$	$3d_{5/2}$	SymGL	56.0	3.50	8.2	14
	$3d_{3/2}$	SymGL	56.7	3.50	5.6	

Table S4. Details of the fit of the Se 3d region of the Ni_3Se_2 reference sample. The peak positions, FWHM, and area of the peak components in % are listed together with concentrations of the species in %.

Species	Peak	Line Shape	Position / eV	FWHM / eV	Area / %	Species Conc. / %
NiSe	$3d_{5/2}$	SymGL	53.6	0.73	18.3	31
	$3d_{3/2}$	SymGL	54.2	0.73	12.6	
Ni_3Se_2	$3d_{5/2}$	SymGL	54.4	0.70	33.9	57
	$3d_{3/2}$	SymGL	55.2	0.70	23.3	
$Ni_XSe_YO_Z$	$3d_{5/2}$	SymGL	56.3	2.78	7.0	12
	$3d_{3/2}$	SymGL	57.1	2.78	4.8	

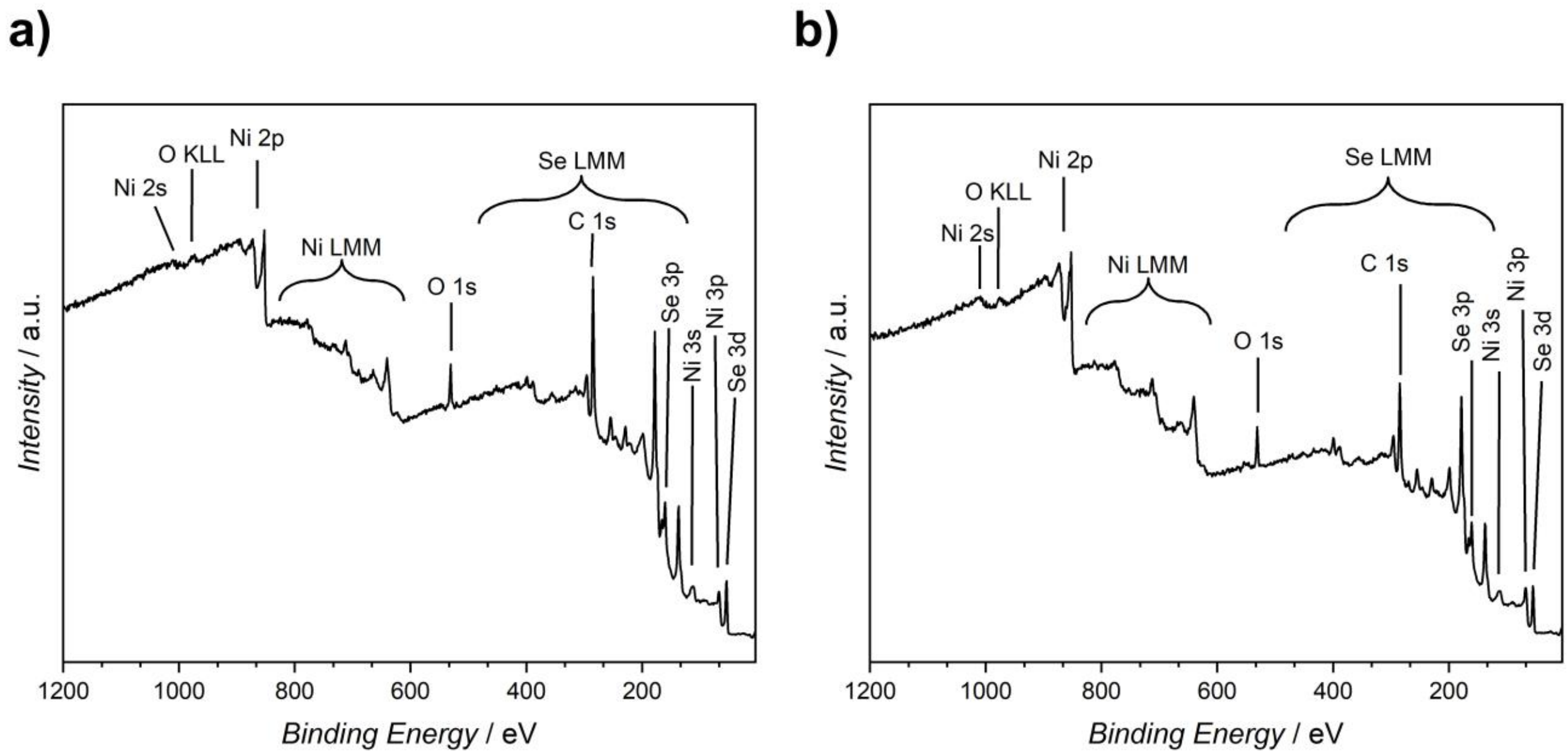

Figure S11. XP survey spectra recorded at a pass energy of 100 eV of (a) the NiSe reference sample and (b) the Ni_3Se_2 reference sample.

2.1.3 Nickel Selenide Films Deposited on Au Modified Carbon Paper

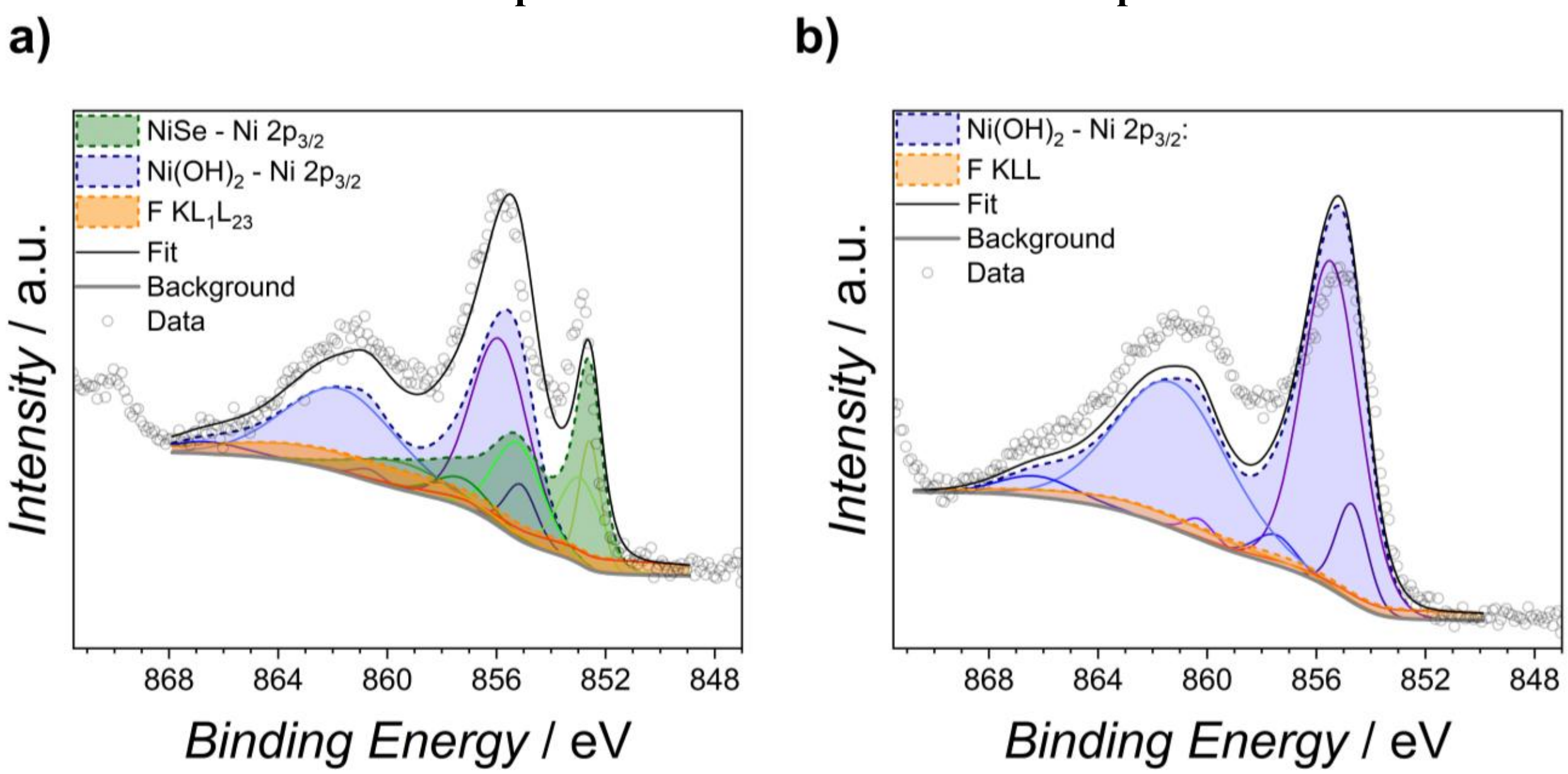

Figure S12. Ni $2p_{3/2}$ XP-spectra of the a) pristine sample and b) the sample after OER. Samples were prepared on Au-modified carbon paper.

The fitting model used for the Ni $2p_{3/2}$ spectrum in b) does not ideally represent the experimental data (Figure S12). No combinations of the peak models of different Ni-species that were considered resulted in a better fit than shown here for the nickel selenide film after OER on Au-modified carbon paper. However, due to the similarities of the O 1s signal and Ni LMM signal (Figure S14 and Table S10) with the film deposited on Au-coated glass after OER, we can conclude that the formed species is likely $Ni(OH)_2$.

Table S5. Details of the fit of the Ni $2p_{3/2}$ region of pristine nickel selenide film on Au-modified carbon paper based on reference models (SI-2.1.2). The peak positions, FWHM, and area of the peak components in % are listed together with concentrations of the species with standard deviation in %.

Species	Peak	Line Shape	Position / eV	FWHM / eV	Area / %	σ(Area) / %	Species Conc. Conc. / %	σ(Conc.) / %
NiSe	1	GL(30)	852.6	0.93	7.1	0.6	39	5
	2	GL(30)	852.9	1.89	9.6	0.9		
	3	GL(30)	855.2	2.10	11.9	1.0		
	4	GL(30)	857.3	2.44	4.0	0.4		
	5	GL(30)	859.5	4.20	6.7	0.6		
$Ni(OH)_2$	1	GL(30)	855.1	1.36	4.5	0.4	61	5
	2	GL(30)	855.9	2.49	27.6	1.6		
	3	GL(30)	857.9	1.79	1.8	0.1		
	4	GL(30)	860.7	1.26	0.9	0.1		
	5	GL(30)	861.7	4.85	23.8	1.4		
	6	GL(30)	866.6	3.21	2.2	0.1		
F KL_1L_{23}	1	GL(30)	852.1	10.82	N.A.	N.A.	N.A.	N.A.
	2	GL(30)	861.8	9.82	N.A.	N.A.		

Table S6. Details of the fit of the Ni $2p_{3/2}$ region of the nickel selenide film on Au-modified carbon paper after OER sample based on reference models (SI-2.1.2). The peak positions, FWHM, and area of the peak components in % are listed together with concentrations of the species with standard deviation in %.

Species	Peak	Line Shape	Position / eV	FWHM / eV	Area / %	σ(Area) / %	Species Conc.	
							Conc. / %	σ(Conc.) / %
$Ni(OH)_2$	1	GL(30)	854.7	1.36	7.4	N.A.	100	N.A.
	2	GL(30)	855.5	2.49	44.2	N.A.		
	3	GL(30)	857.5	1.79	3.1	N.A.		
	4	GL(30)	860.3	1.26	1.4	N.A.		
	5	GL(30)	861.3	4.85	39.5	N.A.		
	6	GL(30)	866.2	3.21	4.4	N.A.		
F KL_1L_{23}	1	GL(30)	852.5	10.82	N.A.	N.A.	N.A.	N.A.
	2	GL(30)	862.2	9.82	N.A.	N.A.		

Table S7. Details of the fit of the Se 3d region of the pristine nickel selenide film on Aumodified carbon paper. The peak positions, FWHM, and area of the peaks in % are listed together with concentration of the species in %.

Species	Peak	Line Shape	Position / eV	FWHM / eV	Area / %	Species Conc. / %
NiSe	$3d_{5/2}$	SymGL	53.9	0.78	36.7	62
	$3d_{3/2}$	SymGL	54.7	0.78	25.2	
NiSe surface states or Ni_3Se_2	$3d_{5/2}$	SymGL	55.0	0.87	9.5	22
	$3d_{3/2}$	SymGL	55.8	0.87	6.5	
SeO_2	$3d_{5/2}$	SymGL	58.5	1.87	13.1	16
	$3d_{3/2}$	SymGL	59.2	1.87	8.9	

Table S8. Details of the peaks used to model the F KLL peaks obtained from the XPS measurement of the PTFE-treated carbon paper substrate.

Signal	Peak	Line Shape	Position / eV	FWHM / eV	Area / %
F $KL_{23}L_{23}$	1	GL(30)	833.1	11.02	61
F KL_1L_{23}	1	GL(30)	852.3	11.02	13
	2	GL(30)	862.0	10.02	16
F KL_1L_1	1	GL(30)	879.1	13.32	9

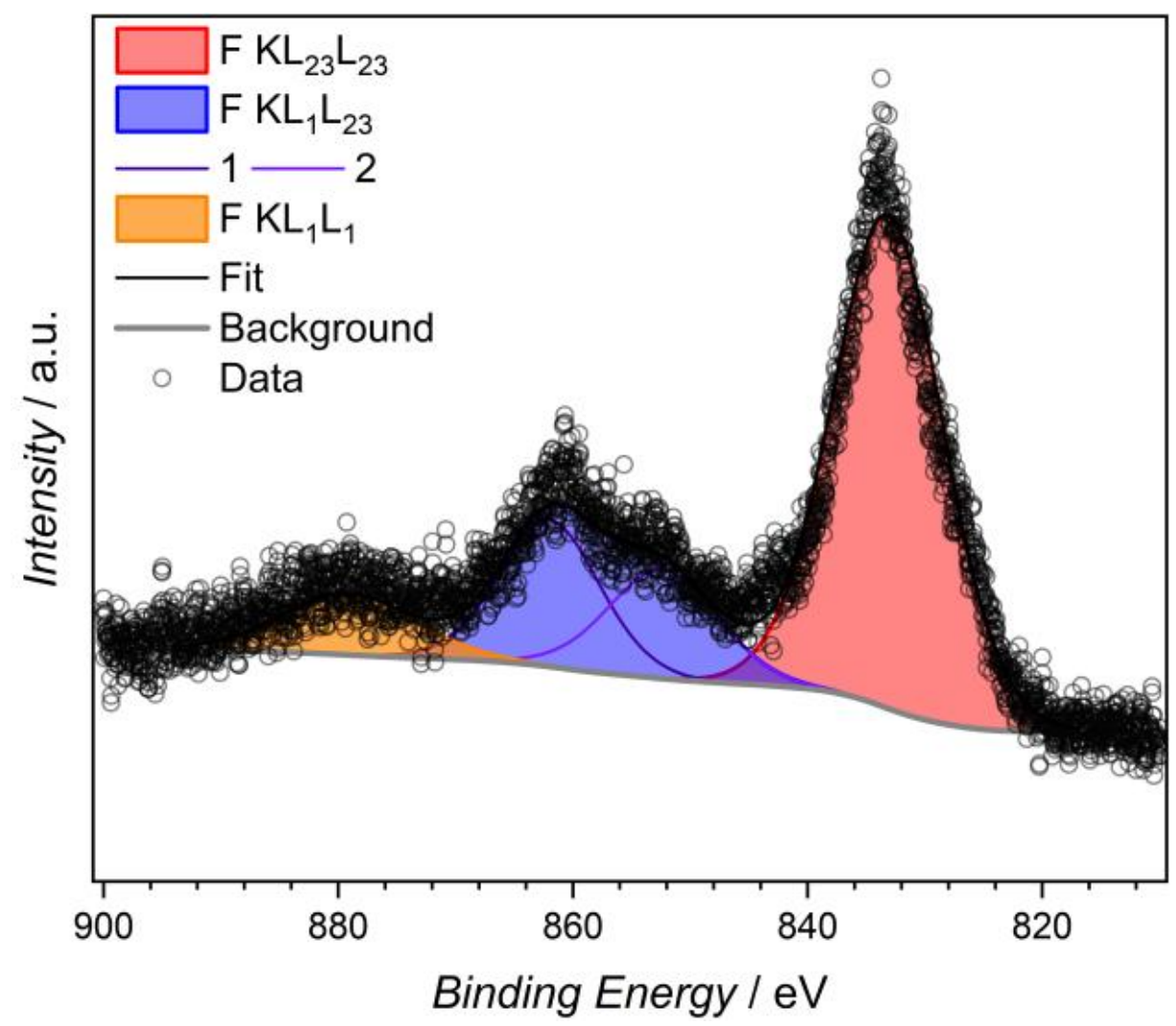

Figure S13. F KLL structure of the X-ray induced Auger emission recorded in the XP spectrum of the of the PTFE-treated carbon paper.

The spectral region with the F KLL signal of the X-ray induced Auger emission is shown against a binding energy scale (instead of the kinetic energy scale) to allow a straightforward comparison with the Ni 2p spectra where spectral overlap would occur.

2.1.4 Nickel Selenide Films Deposited on Au-Coated Glass Slides

Table S9. Details of the fit of the Ni $2p_{3/2}$ region of pristine nickel selenide film on Au-coated glass slide based on reference spectra (see SI-2.1.2). The peak positions, FWHM, and area of the peak components in % are listed together with concentrations of the species with standard deviation in %.

Species	Peak	Line Shape	Position / eV	FWHM / eV	Area / %	σ(Area) / %	Species Conc.	
							Conc. / %	σ(Conc.) / %
NiSe	1	GL(30)	852.6	0.93	3.2	0.3	18	2
	2	GL(30)	852.9	1.89	4.4	0.5		
	3	GL(30)	855.2	2.10	5.4	0.6		
	4	GL(30)	857.3	2.44	1.8	0.2		
	5	GL(30)	859.5	4.20	3.1	0.3		
$Ni(OH)_2$	1	GL(30)	855.1	1.36	6.1	0.1	82	2
	2	GL(30)	855.9	2.49	37.3	0.8		
	3	GL(30)	857.9	1.79	2.5	0.1		
	4	GL(30)	860.7	1.26	1.2	0.1		
	5	GL(30)	861.7	4.85	32.1	0.7		
	6	GL(30)	866.6	3.21	3.0	0.8		

Table S10. Details of the fit of the Ni $2p_{3/2}$ region of the after OER sample of nickel selenide film on Au-coated glass slide based on reference spectra (SI-2.1.2). The peak positions, FWHM, and area of the peak components in % are listed together with concentrations of the species with standard deviation in %.

Species	Peak	Line Shape	Position / eV	FWHM / eV	Area / %	σ(Area) / %	Species Conc.	
							Conc. / %	σ(Conc.) / %
$Ni(OH)_2$	1	GL(30)	855.0	1.27	7.4	N.A.	100	N.A.
	2	GL(30)	855.8	2.40	45.4	N.A.		
	3	GL(30)	857.8	1.70	3.0	N.A.		
	4	GL(30)	860.6	1.17	1.4	N.A.		
	5	GL(30)	861.6	4.76	39.1	N.A.		
	6	GL(30)	866.5	3.12	3.7	N.A.		

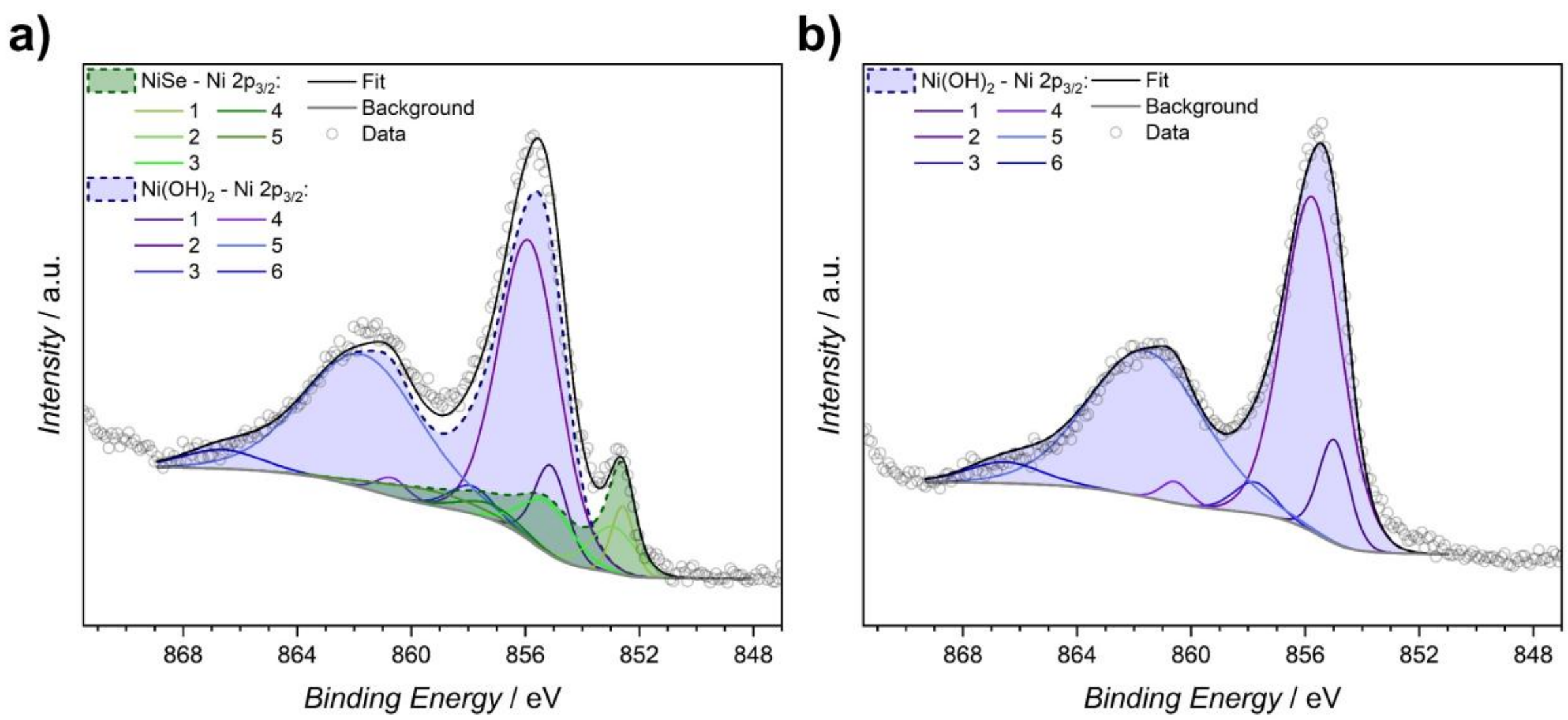

Figure S14. Ni $2p_{3/2}$ XP spectra of a) pristine nickel selenide sample and b) nickel selenide after OER. Both samples were electrodeposited on Au-coated glass slides.

Due to the low selenium content after OER determined from the Ni 3p and Se 3d peaks in the survey spectrum, no NiSe species was considered for the fit of the Ni $2p_{3/2}$ spectrum, as a species with this low content cannot be reasonably deconvoluted in the complex Ni $2p_{3/2}$ spectrum.

Table S11. Details of the fit of the Se 3d region of the pristine nickel selenide film on Au-coated glass slide. The peak positions, FWHM, and area of the peaks in % are listed together with concentration of the species in %.

Species	Peak	Line Shape	Position / eV	FWHM / eV	Area / %	Species Conc. / %

NiSe	$3d_{5/2}$<br>$3d_{3/2}$	SymGL<br>SymGL	54.0<br>54.7	0.76<br>0.76	29.6<br>20.4	50
NiSe surface states or Ni_3Se_2	$3d_{5/2}$<br>$3d_{3/2}$	SymGL<br>SymGL	55.1<br>55.8	0.75<br>0.75	8.9<br>6.1	15
SeO_2	$3d_{5/2}$<br>$3d_{3/2}$	SymGL<br>SymGL	58.6<br>59.2	1.63<br>1.63	20.7<br>14.2	35

Table S12. Details of the fit of the Se 3d region of the nickel selenide film on Au-coated glass slide after OER. The peak positions, FWHM, and area of the peaks in % are listed together with concentration of the species in %.

Species	Peak	Line Shape	Position / eV	FWHM / eV	Area / %	Species Conc. / %
NiSe	$3d_{5/2}$<br>$3d_{3/2}$	SymGL<br>SymGL	53.9<br>54.6	0.82<br>0.82	37.1<br>25.6	63
NiSe surface states or Ni_3Se_2	$3d_{5/2}$<br>$3d_{3/2}$	SymGL<br>SymGL	55.0<br>55.7	0.82<br>0.82	11.0<br>7.6	19
SeO_2	$3d_{5/2}$<br>$3d_{3/2}$	SymGL<br>SymGL	57.9<br>58.6	2.61<br>2.61	11.1<br>7.6	19

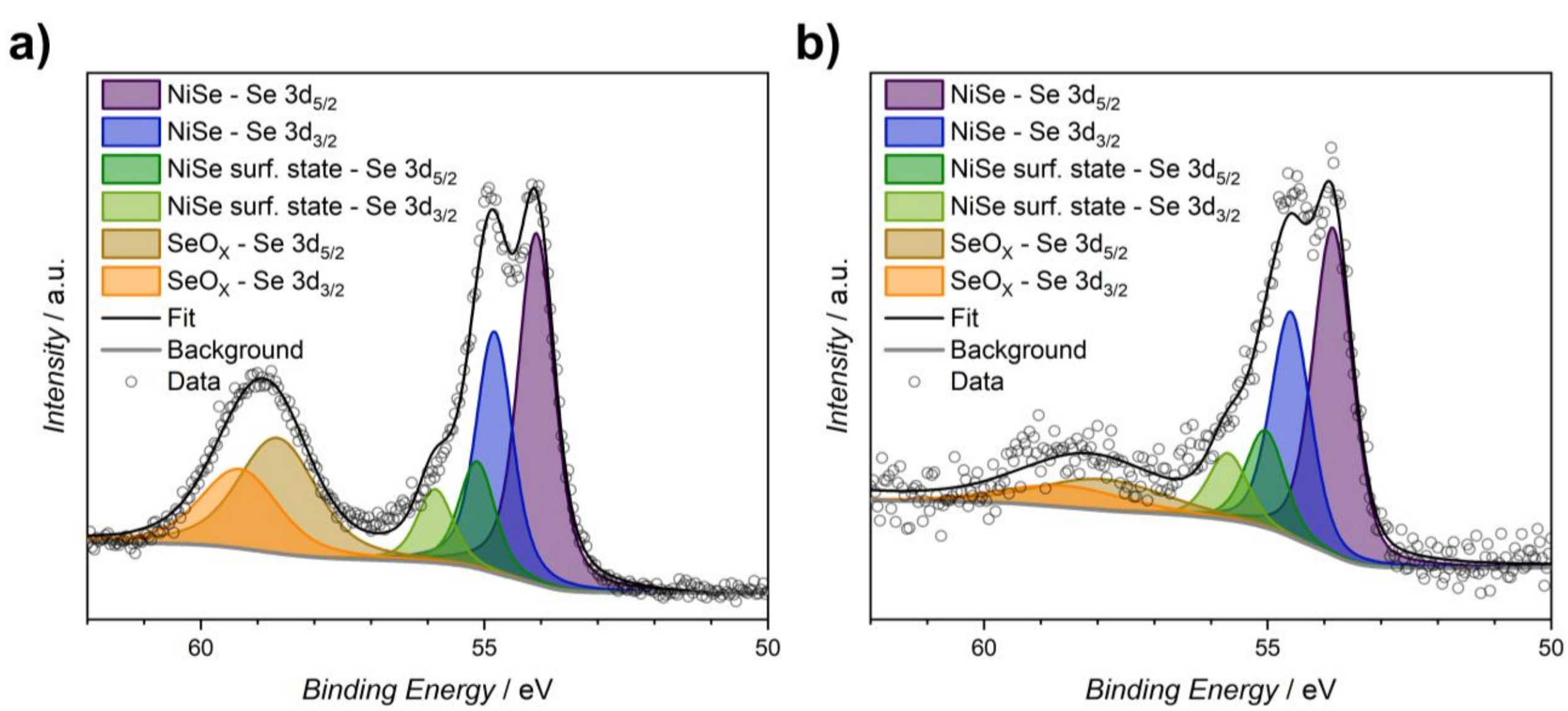

Figure S15. XP-spectra of the Se 3d region of a) pristine nickel selenide sample and b) nickel selenide sample after OER. Both nickel selenide samples were electrodeposited on Au-coated glass slides.

2.1.5 Details of O 1s Peak Fitting Deposited on Au-Coated Glass Slides and Au-Modified Carbon Paper

Table S13. Details of the fit of the O 1s region of the nickel selenide samples on Au-coated glass slides and Au-modified carbon paper. The peak positions, FWHM, the constraints on the FWHM, and area of the peak components in % are listed together.

Pristine Nickel selenide film – Au-coated glass slides							
Species	Peak	Line Shape	Position / eV	FWHM / eV	FWHM Constr. / eV	Area / %	σ(Area) / %
Hydroxides	A	GL(30)	531.1	2.00	0.4 , 2	96	2
Ads. Water, Organics	B	GL(30)	533.0	2.00	A · 1	4	2
**After OER Nickel selenide film – Au-coated glass slides**							
Species	Peak	Line Shape	Position / eV	FWHM / eV	FWHM Constr. / eV	Area / %	σ(Area) / %
Hydroxides	A	GL(30)	531.1	1.84	0.4 , 2	93	2
Ads. Water, Organics	B	GL(30)	533.0	1.84	A · 1	3	1
Lattice Oxide	C	GL(30)	528.4	1.19	0.4 , 1.5	3	1
**Pristine Nickel selenide film – Au-modified carbon paper**							
Species	Peak	Line Shape	Position / eV	FWHM / eV	FWHM Constr. / eV	Area / %	σ(Area) / %
Hydroxides	A	GL(30)	531.3	1.97	0.4 , 2	95	5
Ads. Water, Organics	B	GL(30)	533.5	1.97	A · 1	5	5
**BorAfter OER Nickel selenide film – Au-modified carbon paper**							
Species	Peak	Line Shape	Position / eV	FWHM / eV	FWHM Constr. / eV	Area / %	σ(Area) / %
Hydroxides	A	GL(30)	530.8	1.83	0.4 , 2	79	1
Ads. Water, Organics	B	GL(30)	532.5	1.83	A · 1	21	1

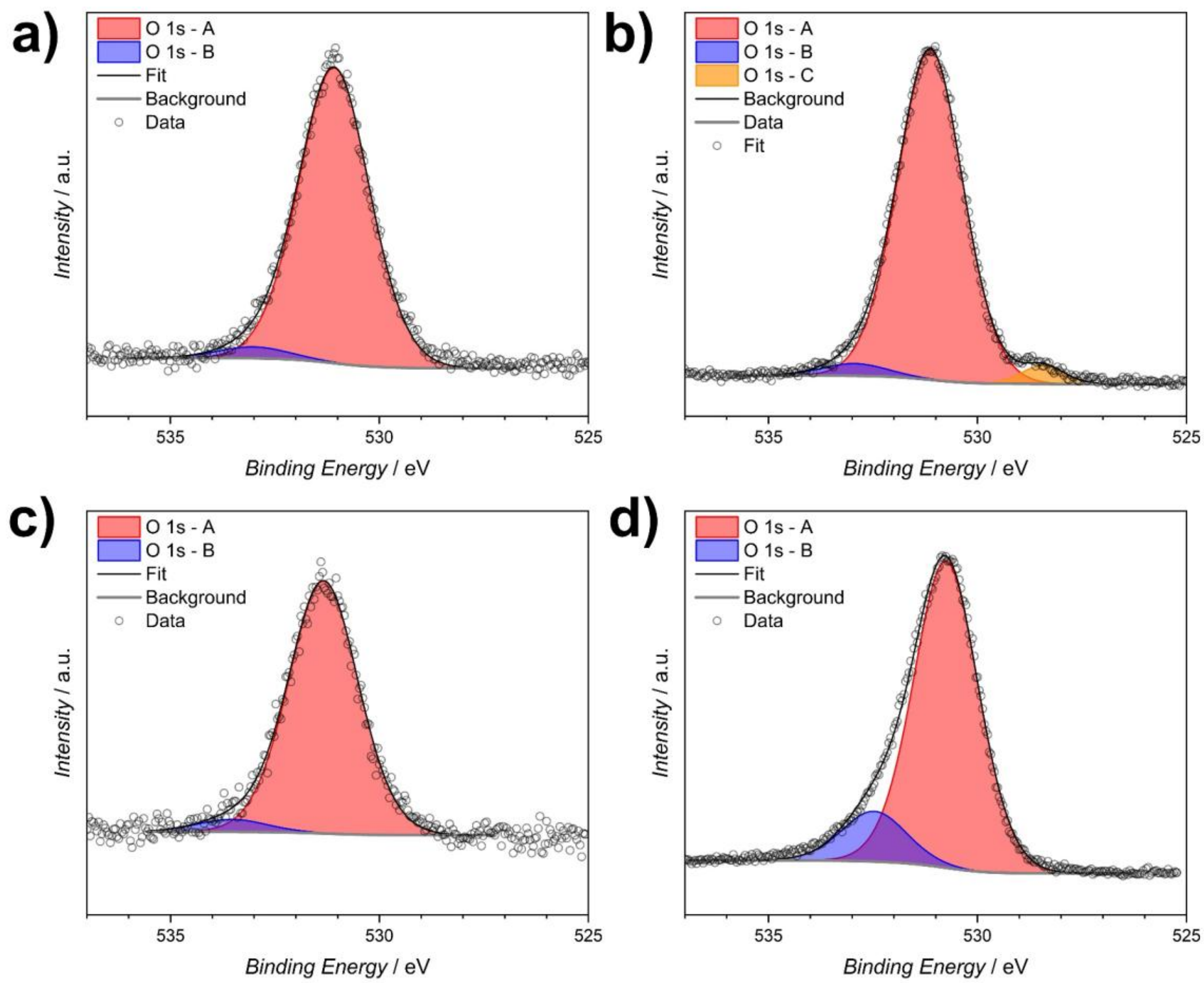

Figure S16. XP-spectra of the O 1s region of (a) the pristine nickel selenide and (b) after OER of the films deposited on Au-coated glass slides and (c) the pristine nickel selenide and (d) after OER of the films deposited on Au-modified carbon paper.

The peak labeled A in the O 1s of the as-deposited nickel selenide film and the nickel selenide film after OER could be related to the hydroxide species in nickel hydroxides.[25] For the as-deposited sample, a contribution of the oxygenated selenium species (SeO_x) that were identified in the Se 3d region is expected. The O 1s peak of SeO_2 is observed at 531.5 eV (Figure S19b), and SeO_x species, hence likely contribute to the O 1s-A peak. The peak labeled B in the O 1s spectra could be attributed to adsorbed water, hydrated $Ni(OH)_2$, or oxygenated organic contaminations.[25,26] The peak labeled C that was identified in the nickel selenide film on Au-coated glass after OER is likely related to lattice oxygen of the minor Zn contamination (Figure S16b) found on this sample, probably resulting from contaminations of the used KOH and Zn hydroxide deposition under electrochemical conditions.[27]

2.1.6 Comparison of the Ni LMM Auger Peak of the Samples after OER

To further investigate why a better fit could not be achieved for the Ni $2p_{3/2}$ of the nickel selenide film electrodeposited on Au-Carbon paper after OER, the Ni LMM Auger peaks of the samples electrodeposited on Au-carbon paper and Au-glass were compared. The shape of the Ni LMM emission from Ni materials can often be a descriptor of different surface species.[28] As the shapes of the Ni LMM signal of the samples electrodeposited on the different substrates are very similar, we conclude that the complications with the fitting of the Ni $2p_{2/3}$ of the sample on Au-carbon paper are likely not related to a different nickel species.

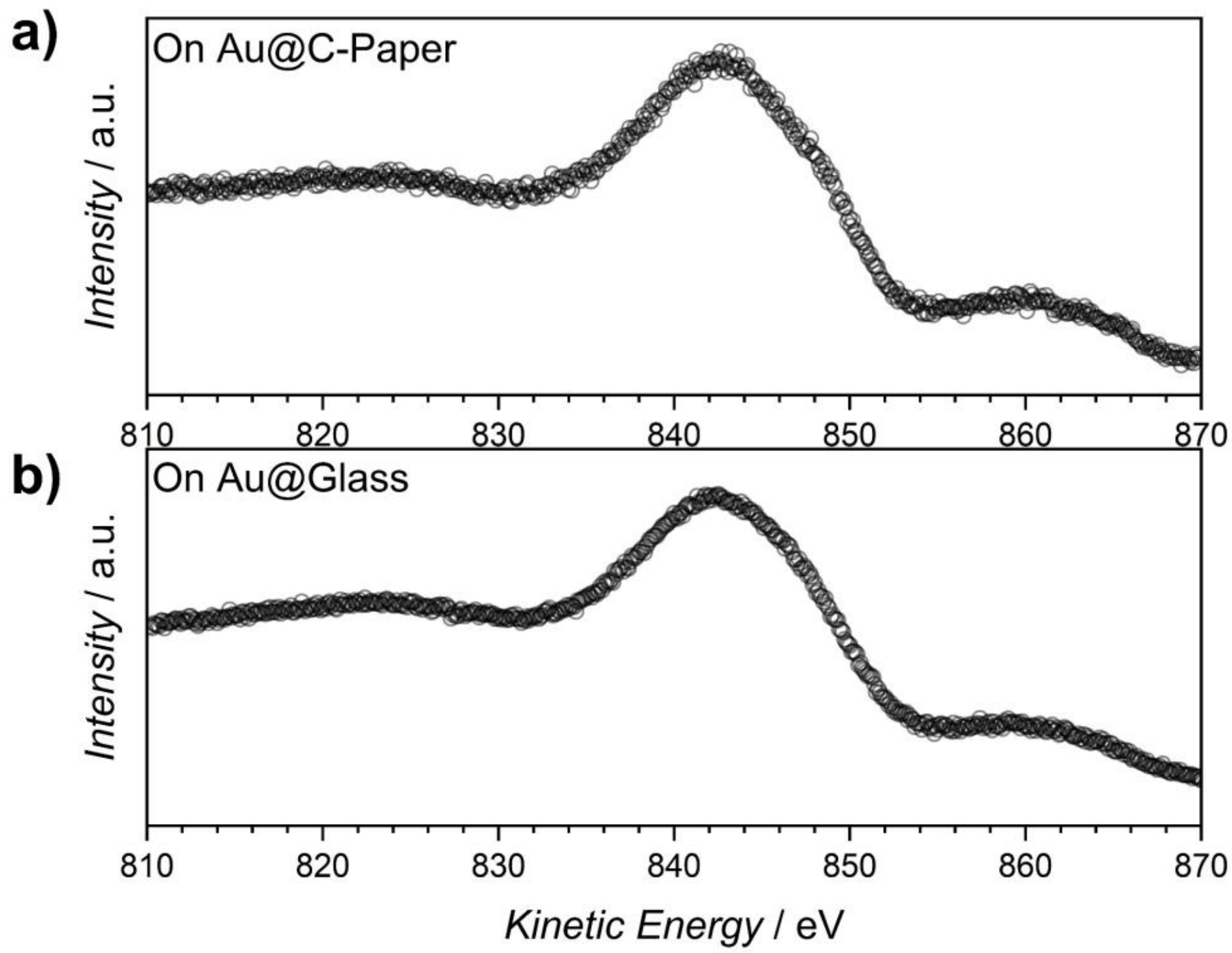

Figure S17. XP-spectra of the Ni LMM of the nickel selenide films that were electrodeposited on a) Au-modified carbon paper and b) Au-coated glass slide after OER.

2.1.7 Survey Scans of the Nickel Selenide Films

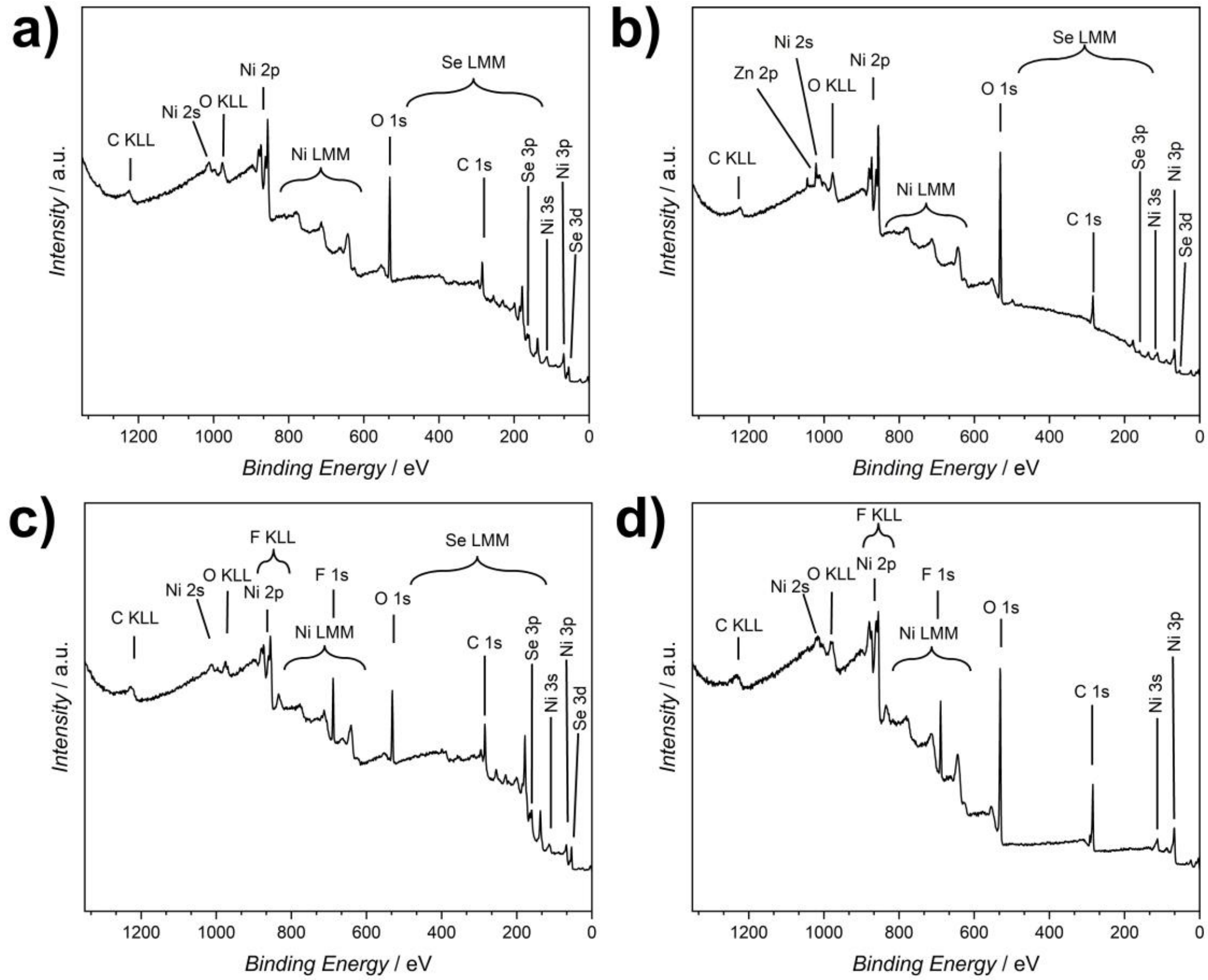

Figure S18. XP survey spectra recorded at a pass energy of 100 eV of (a) the as-deposited nickel selenide film and (b) after OER deposited on Au-coated glass slide and (c) the as-deposited nickel selenide film, and (d) after OER deposited on Au-modified carbon paper.

The survey spectra of the samples show peaks related to Ni, Se, O, and C. For the samples deposited on PTFE-treated carbon paper, signals related to F are also observed. For the nickel selenide film on Au-coated glass after OER, a minor contamination of Zn is also observed, probably resulting from contaminations of the used KOH and Zn hydroxide deposition under electrochemical conditions[27] as discussed above (Figure S16b). To estimate the composition of the catalyst film in the near-surface region, the Ni 3p, Se 3d, and O 1s peaks in the survey scans were integrated and normalized by RSF (Scofield RSFs library in CasaXPS) and transmission function of the device. The results of this are shown in Table S14.

Table S14. Surface composition calculated by integrating the Ni 3p (RSF 2.217), O 1s (RSF 2.930), and Se 3d (RSF 2.294) region from the survey spectra of the as-deposited sample and samples after OER that were electrodeposited on either Au-coated glass slide or Au-modified carbon paper.

Sample		At. % nickel	At. % selenium	At. % oxygen
Pristine	Au-coated glass	29	15	56
After OER	Au-coated glass	27	3	70
Pristine	Au-modified carbon paper	27	23	50
After OER	Au-modified carbon paper	30	N.A.	70

2.1.8 XP-Spectra of Selenium Dioxide

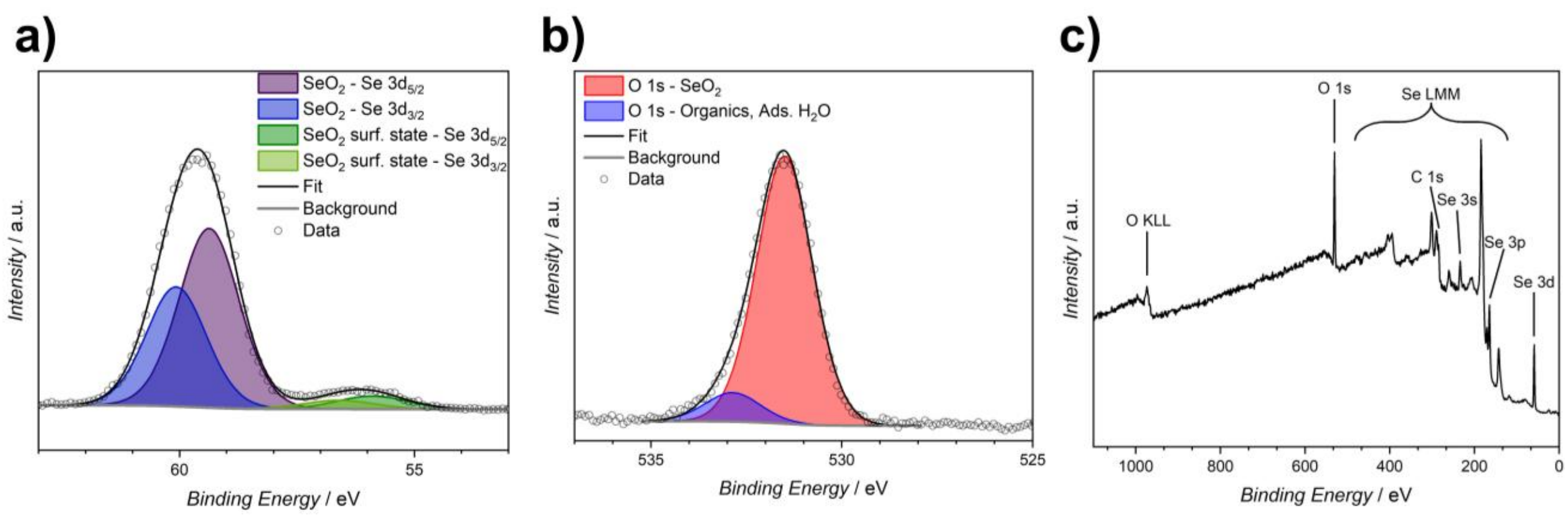

Figure S19. XP-spectra of a SeO_2 reference sample with (a) the Se 3d region, (b) the O 1s region, and (c) the survey spectrum.

Table S15. Details of the fit of the Se 3d region of a SeO_2 reference sample. The peak positions, FWHM, and area of the peaks in % are listed together with concentration of the species in %. The second species at lower binding energy may be related to surface states of SeO_2 or reduced SeO_X (SeO_2 reduced by thermal electrons from charge compensation).

Species	Peak	Line Shape	Position / eV	FWHM / eV	Area / %	Species Conc. / %
SeO_2	$3d_{5/2}$	GL(30)	59.6	1.51	54.6	92
	$3d_{3/2}$	GL(30)	50.6	1.51	37.5	
SeO_2 – surface states	$3d_{5/2}$	GL(30)	55.9	1.76	4.7	8
	$3d_{3/2}$	GL(30)	56.6	1.76	3.2	

Table S16. Details of the fit of the O 1s region of a SeO_2 reference sample. The peak positions, FWHM, the constraints on the FWHM, and area of the peak components in % are listed together.

Species	Line shape	Position / eV	FWHM / eV	FWHM constr. / eV	Area / %	σ(area) / %
SeO_2	GL(30)	531.5	1.72	0.4 , 2	90	2
Ads. water, organics	GL(30)	532.9	1.72	A · 1	10	2

2.2 Au-RDE vs. XAS-Cell

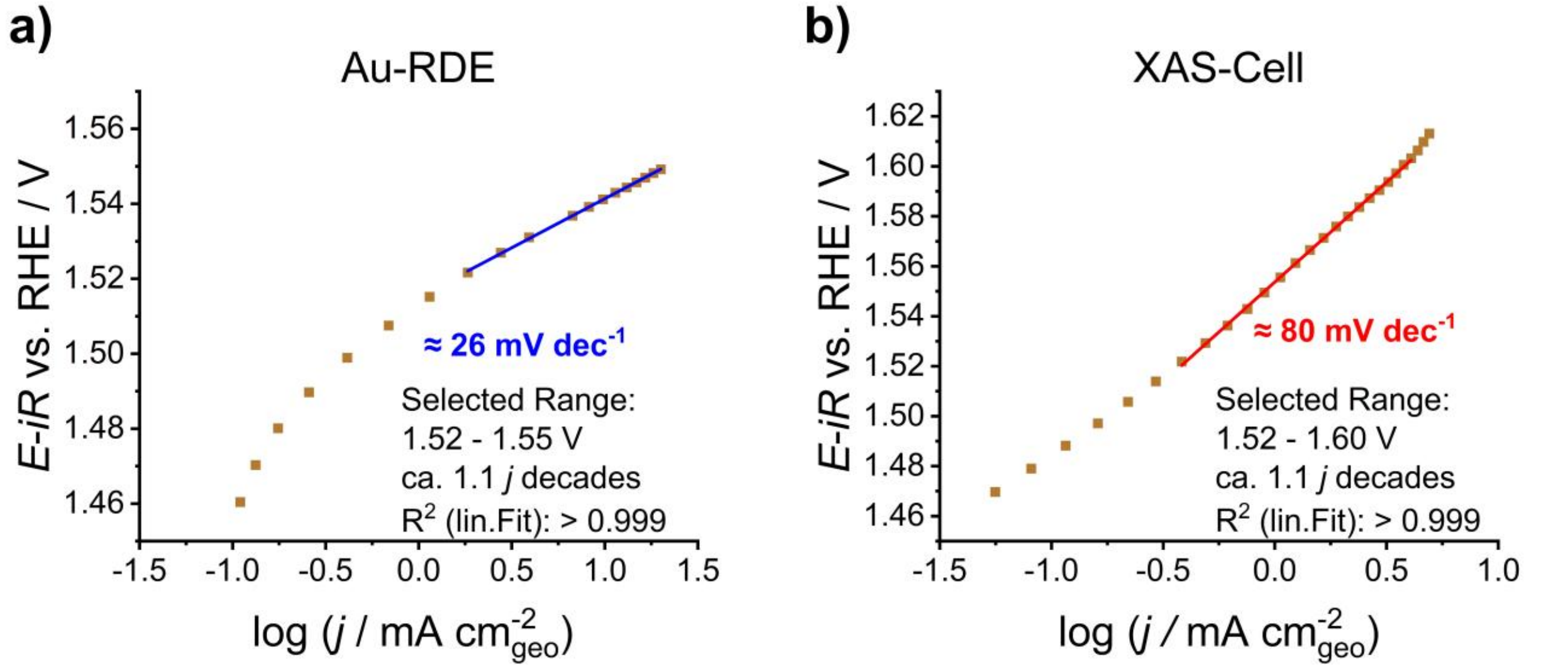

Figure S20. Tafel plots of electrodeposited nickel selenide with a similar amount of deposited charge (Q_{dep} = 1.1 C cm^{-2}) on a) an Au-RDE at a rotation rate of 4000 rpm and b) in the *operando* XAS cell on Au-modified carbon paper. Both Tafel plots were generated using the script described in SI-1.4. Tafel slopes were extracted in the same potential range (1.52 V – 1.55 V vs. RHE). The significant differences in Tafel slope values could be explained by a significant impact of bubble formation and mass transport limitation on the extraction of kinetic parameters, as reported in previous literature studies.[29–31] The high rotation rate of the Au-RDE suppresses bubble formation and/or supports their removal. The Tafel slope of 26 mV dec^{-1} is in good agreement with literature reports[4,29] on Ni(Fe) (oxy)hydroxide-based OER electrocatalysts, reflecting their high kinetic activity.

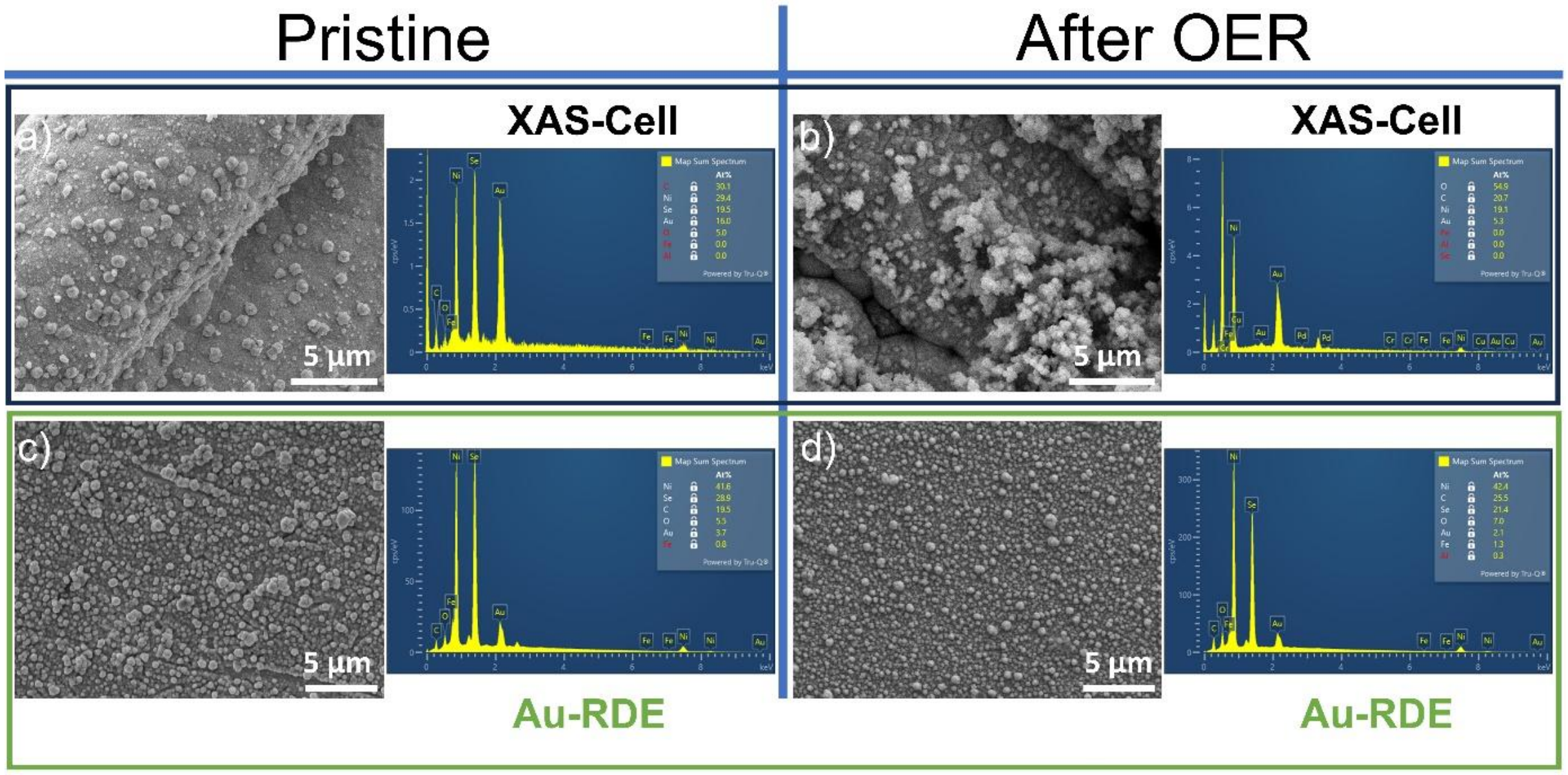

Figure S21. SEM images of a) pristine nickel selenide and b) nickel selenide after OER in the operando XAS cell. Samples were prepared on Au-modified carbon paper. The morphology is significantly roughened after the electrochemical treatment under OER conditions compared to the pristine sample. After OER, the diminished Se content in EDX is due to further transformation of the pristine catalyst material under electrolysis conditions.

Differences between Films on Au-RDE Compared to XAS-cell:

The Tafel slope is significantly higher in the XAS cell on Au-modified carbon paper than on Au-RDE, being influenced by mass transport limitations (≈80 mV dec^{-1}, Figure S20b).[29,32,33] Moreover, the amount and degree of transformation of the pristine electrodeposit seems to be significantly higher on the Au-modified carbon paper than Au-RDE, as indicated by SEM images of pristine and after OER samples (Figure S21).[31] While the character of the nanoparticulate films with grain sizes of 10 – 90 nm remains relatively unchanged on the Au-RDE (Figure S21c+d), the sample after OER on Au-modified carbon paper shows a significantly roughened morphology (Figure S21a+b). This might have originated from the higher and more irregular surface area of the Au-modified carbon paper compared to the Au-RDE. However, ICP-MS data of samples after OER (Table S17) show that ≈45±2% of the total selenium content remains in the electrode material even though it could not have been detected in EDX here. Most likely, the high degree of transformation might be influenced by the rough character and high surface area of the Au-modified carbon paper substrate. On the other hand, SEM images of c) pristine NiSe and d) NiSe-derived material after OER on Au-RDE showed lower differences in both, morphology and composition, between the two sample states compared to the samples on Au-modified carbon paper.

Table S17. *Ex situ* ICP-MS data of Au-modified carbon paper substrates before electrodeposition (blank), after electrodeposition (pristine) and after running the OER EC-script described above (after OER). Ni and Se contents on blank substrates are negligible. Ni content of pristine and after OER samples are similar, while the Se content of the sample after OER decreased to only ≈45±2% of the value in the pristine sample. Since the Fe content in the pristine sample is higher compared to the sample after OER, these data cannot be used to conclude the Ni:Fe ratio in the catalytically active species. Hence, *ex situ* XPS measurements were carried out to evaluate the Ni:Fe ratio.

	β(Fe) / µg L^{-1}	StD β(Fe)	β(Ni) / µg L^{-1}	StD β(Ni)	β(Se) / µg L^{-1}	StD β(Se)	m(Fe) / µg	StD m(Fe)	m(Ni) / µg	StD m(Ni)	m(Se) / µg	StD *m*(Se)
Blank 1	3.98		1.68		< 0.4		0.20		0.08		< 0.02	
Blank 2	3.06		1.32		< 0.4		0.15		0.07		< 0.02	
Blank 3	2.87		1.33		< 0.4		0.14		0.07		< 0.02	
Average Blank	3.30	±0.49	1.44	±0.17			0.17	±0.02	0.07	±0.01		
Pristine 1	4.36		431		346		0.22		21.6		17.3	
Pristine 2	4.55		292		305		0.23		14.6		15.2	
Pristine 3	4.30		616		496		0.22		30.8		24.8	
Average Pristine	4.41	±0.11	446	±132	382	±82	0.22	±0.01	22.3	±6.6	19.1	±4.1
After OER	3.77		547		163		0.19		27.3		8.15	
After OER	3.11		423		175		0.16		21.1		8.74	
Average After OER	3.44	±0.33	485	±62	169	±6	0.17	±0.02	24.2	±3.1	8.45	±0.29

2.3 XRD Data

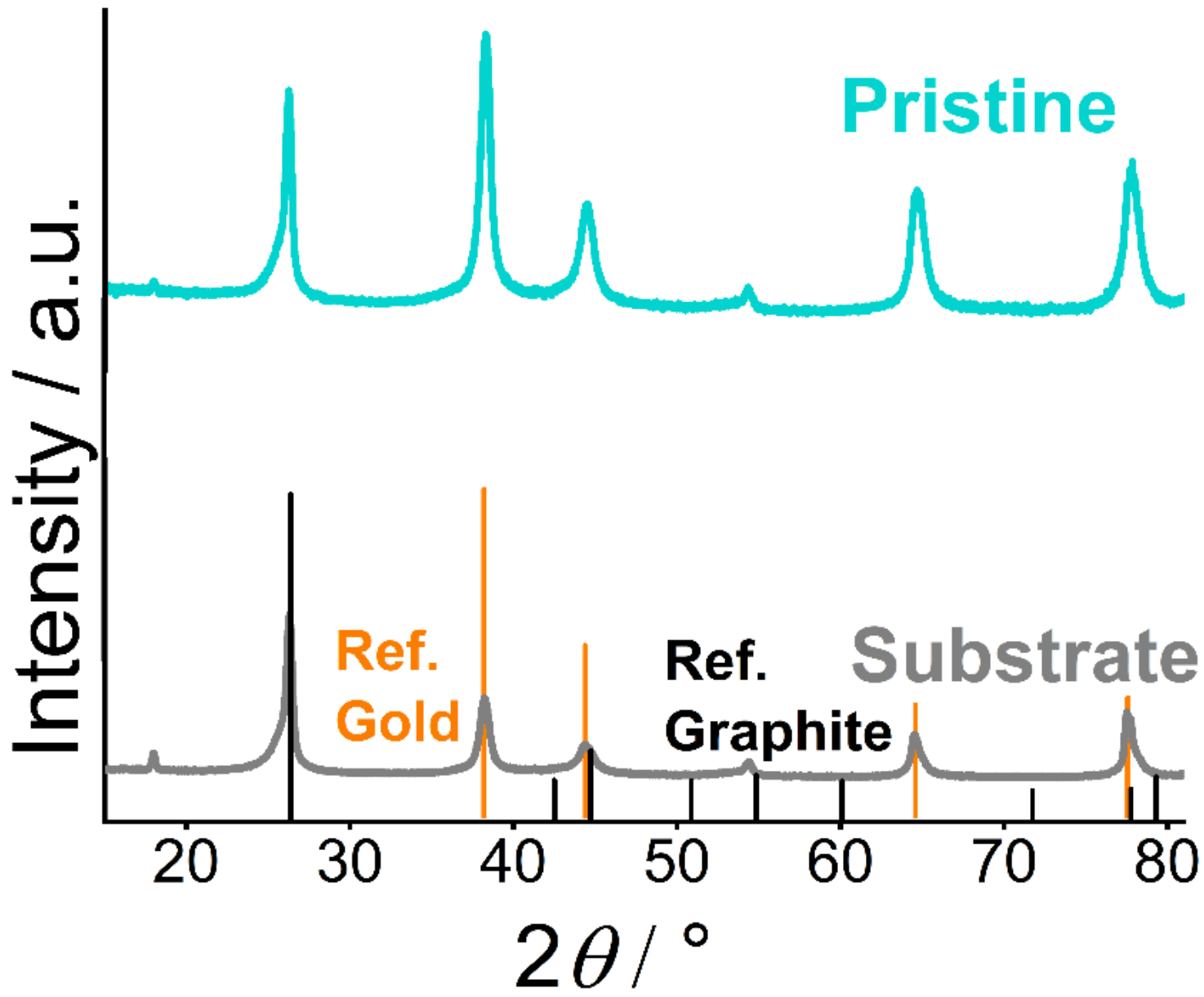

Figure S22. XRD pattern of pristine NiSe shows only signals related to graphite and gold substrate, but not to crystalline NiSe (substrate consisting of electrodeposited Au on carbon paper in grey).

2.4 TEM Data

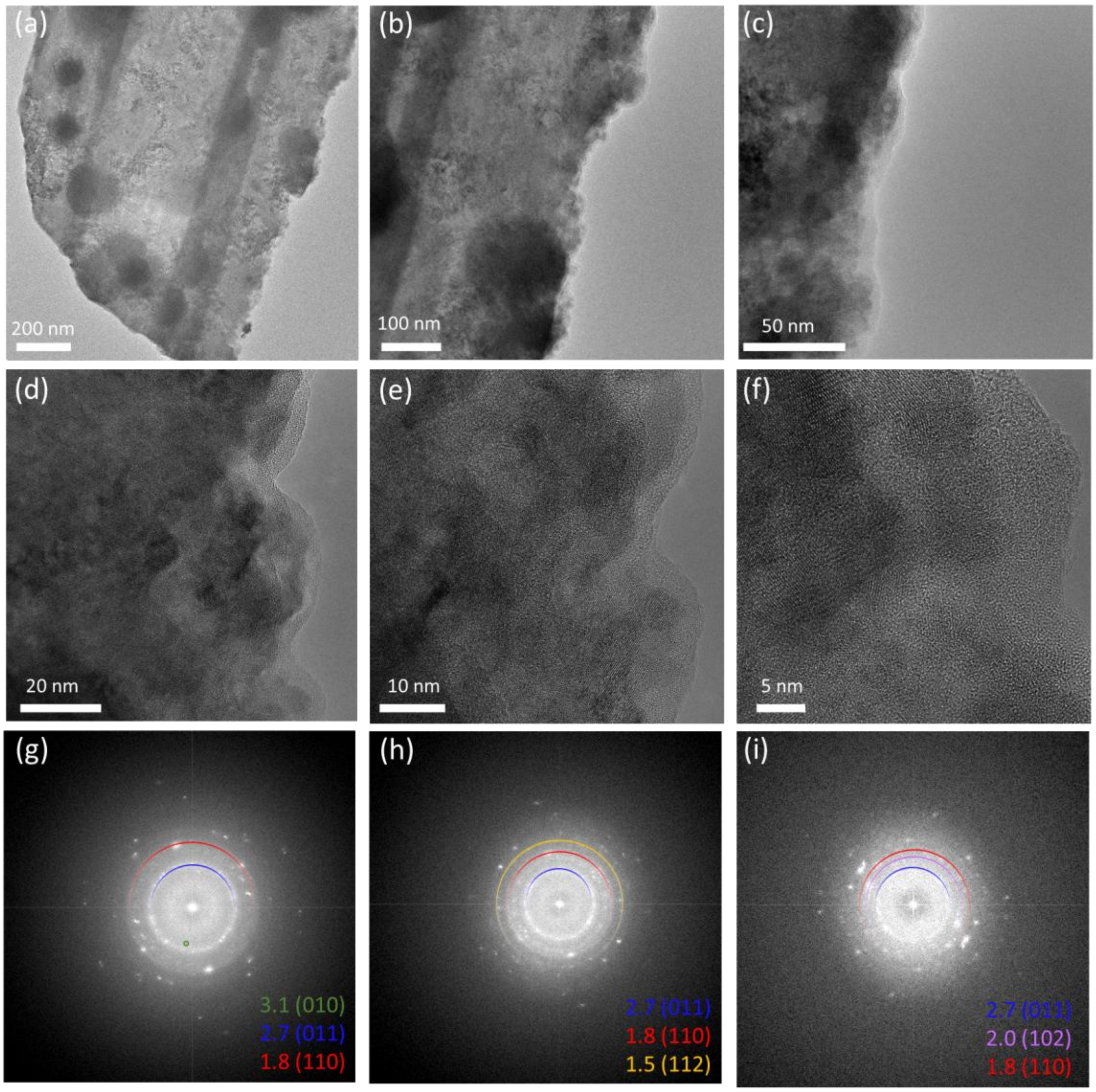

Figure S23. a)-c) TEM, high-resolution TEM images and g)-i) corresponding FFT patterns of the pristine electrodeposited NiSe on Au-modified carbon paper substrate. The FFT patterns demonstrate the $P6_3/mmc$ NiSe nanocrystalline structure of the material, with visible rings and reflections at 3.1, 2.7, 2.0, 1.8, and 1.5 Å among others, corresponding to (010), (011), (102), (110) and (112) planes. Note that in d)-f) images, a surface layer is observed, which can be attributed to the surface oxidation of the material at ambient conditions.

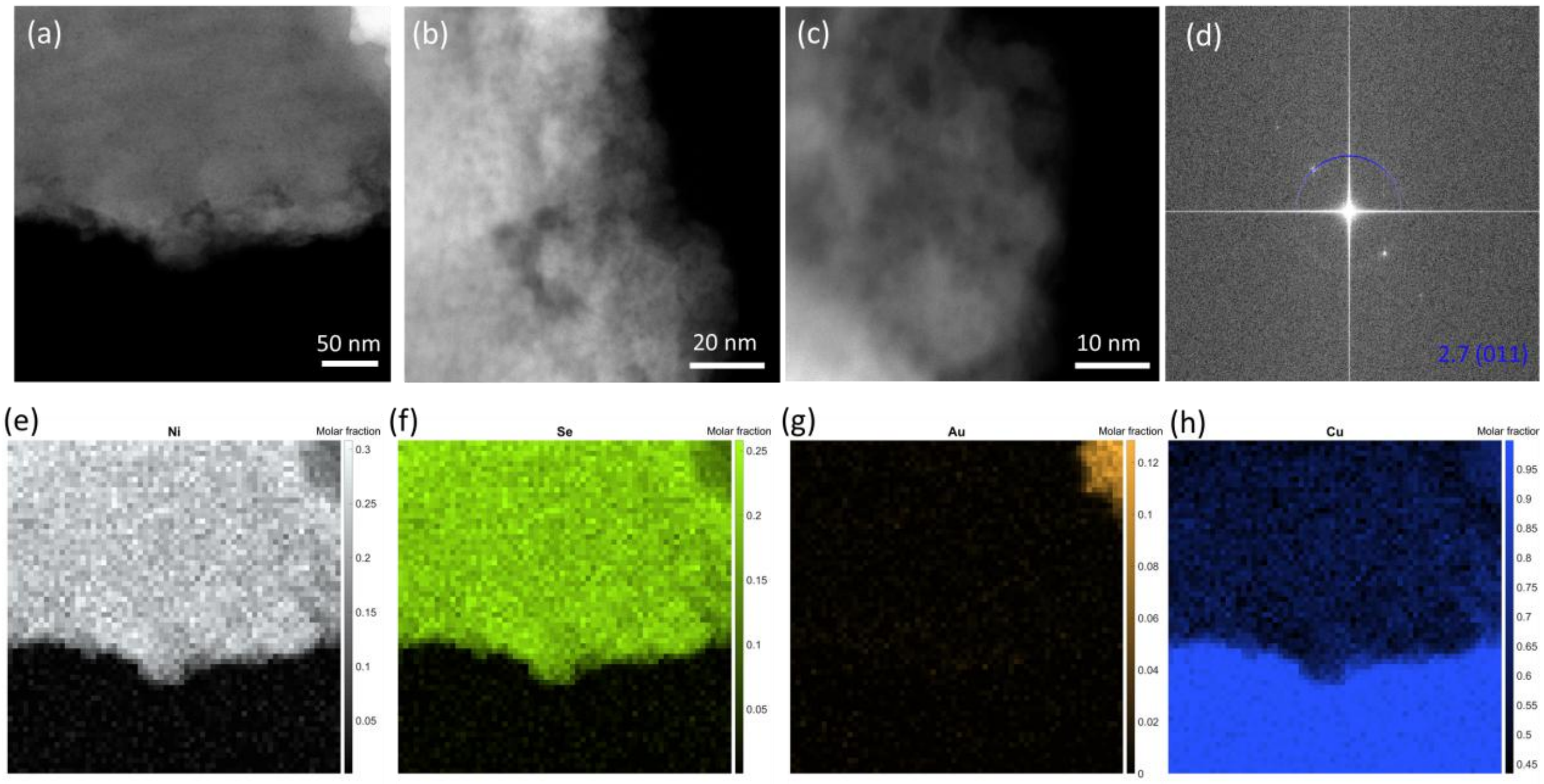

Figure S24. a)-d) STEM images and corresponding FFT pattern with a ring and reflection at 2.7 Å (NiSe (011) plane) of the pristine electrodeposited sample Au-modified carbon paper substrate; e)-h) Quantitative EDX elemental distribution maps for Ni, Se, Au and Cu. Note that the Au signal comes from the Au-modified carbon paper substrate, while Cu originates from the TEM grid.

2.5 XAS Data

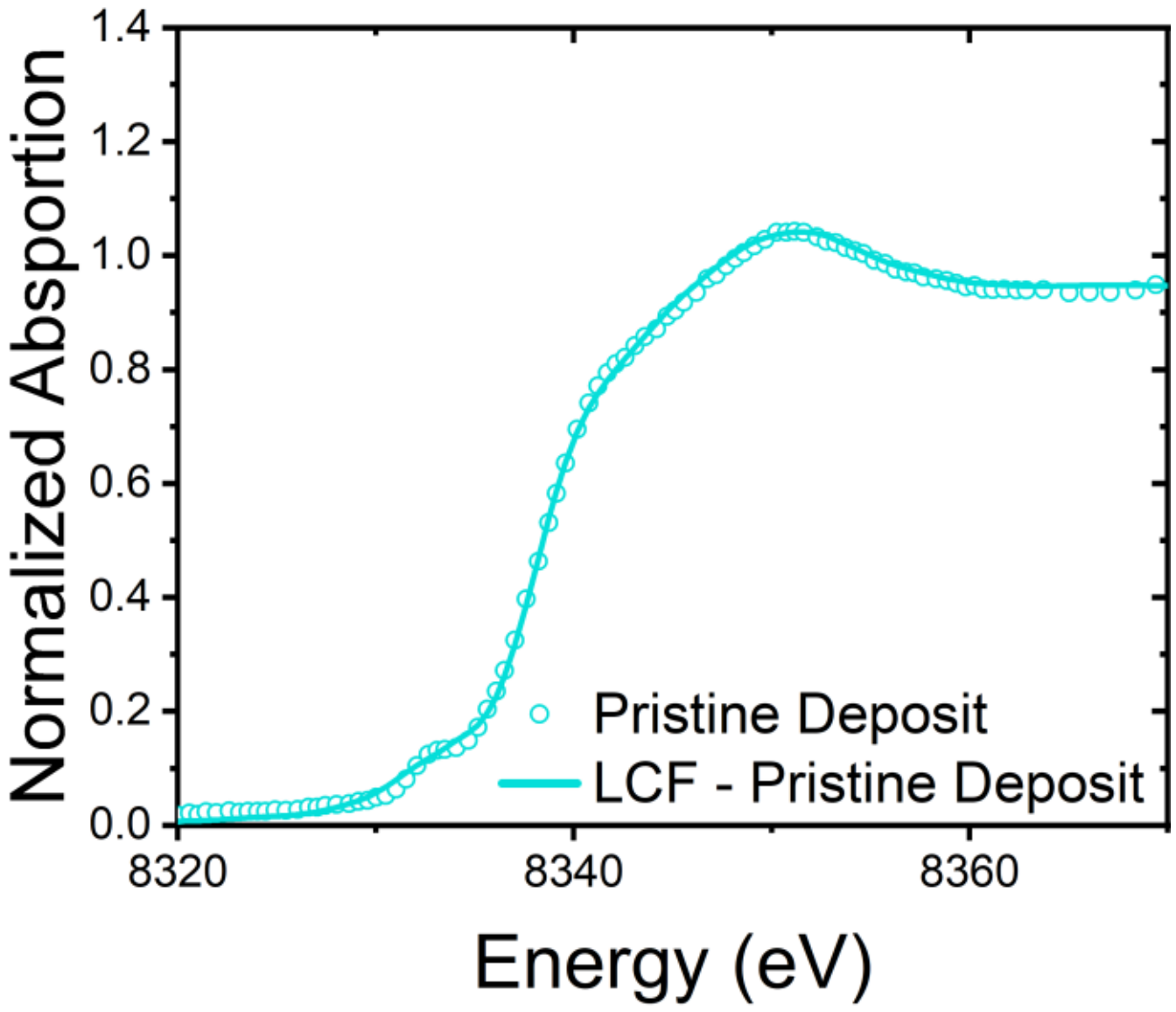

Figure S25. Linear combination fitting (LCF) plots of *ex situ* XAS measurements on Ni K-edge of the pristine deposit on Au-modified carbon paper.

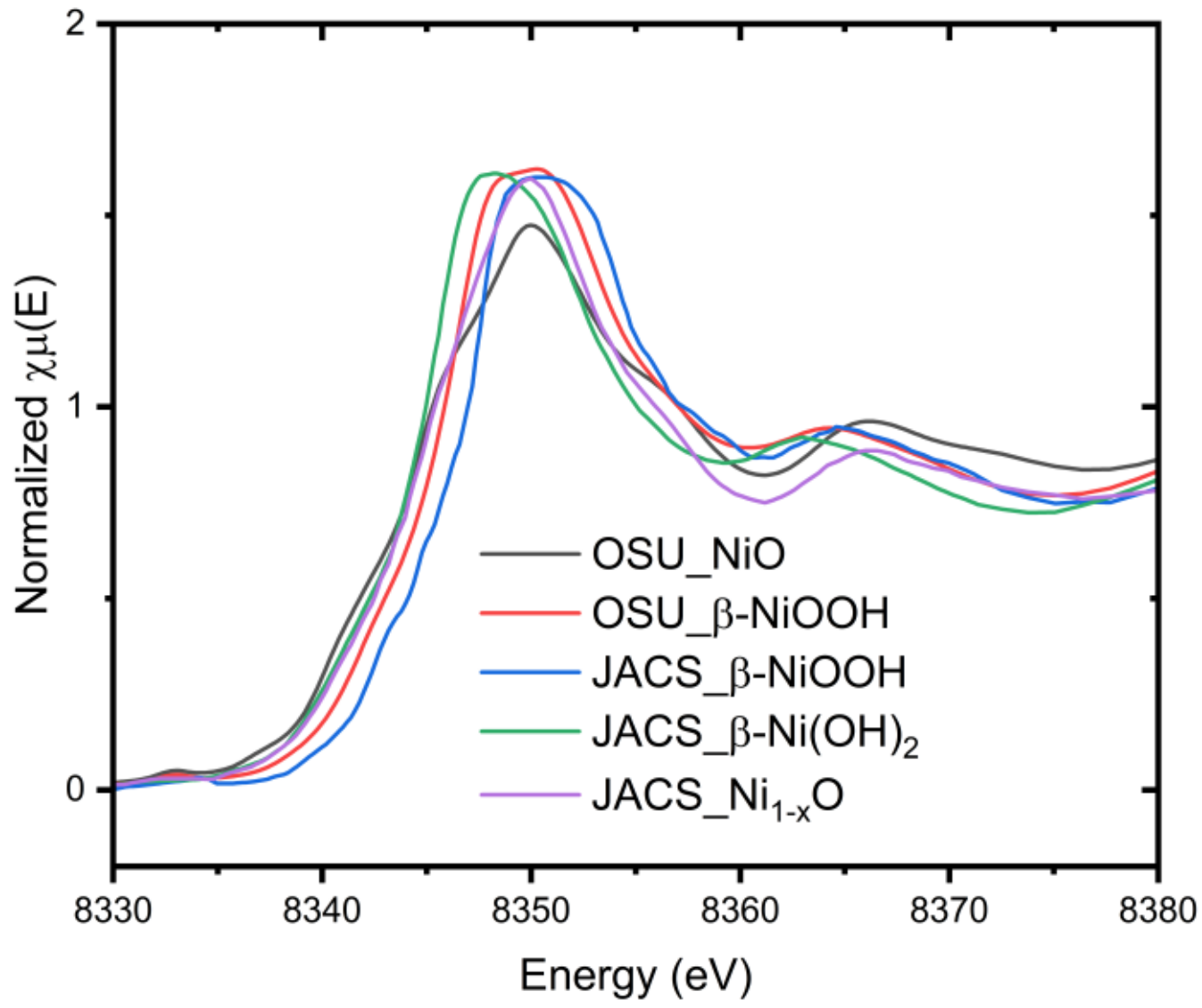

Figure S26. XAS spectra on the Ni K edge showing XANES (8330 – ca. 8380 eV) region of the reference materials used in this work (OSU_NiO and OSU_β-NiOOH) and the spectra of β-NiOOH (JACS_β-NiOOH), β-$Ni(OH)_2$ (JACS_β-$Ni(OH)_2$), and $Ni_{1-x}O$ (JACS_$Ni_{1-x}O$) recorded and published in the work of Yao et al.[34] The comparison of our reference material shows that it is, in fact, not phase-pure β-NiOOH, but "defective, mixed-valence β-NiOOH reference material" with a refined, accurately calculated baseline oxidation state of ~2.5+ to ~2.7+. This can be explained by recent findings that phase-pure β-NiOOH undergoes rapid partial self-discharge and reduction when removed from an applied potential and stored under vacuum conditions[35,36] — a necessary environmental condition for many *ex situ* measurements and beamline preparations. Consequently, we named the used reference sample in Figures 3 and 4 of the main manuscript, as well as Figures S27 and S28 in the SI, as "β-NiOOH (*ex situ*)".

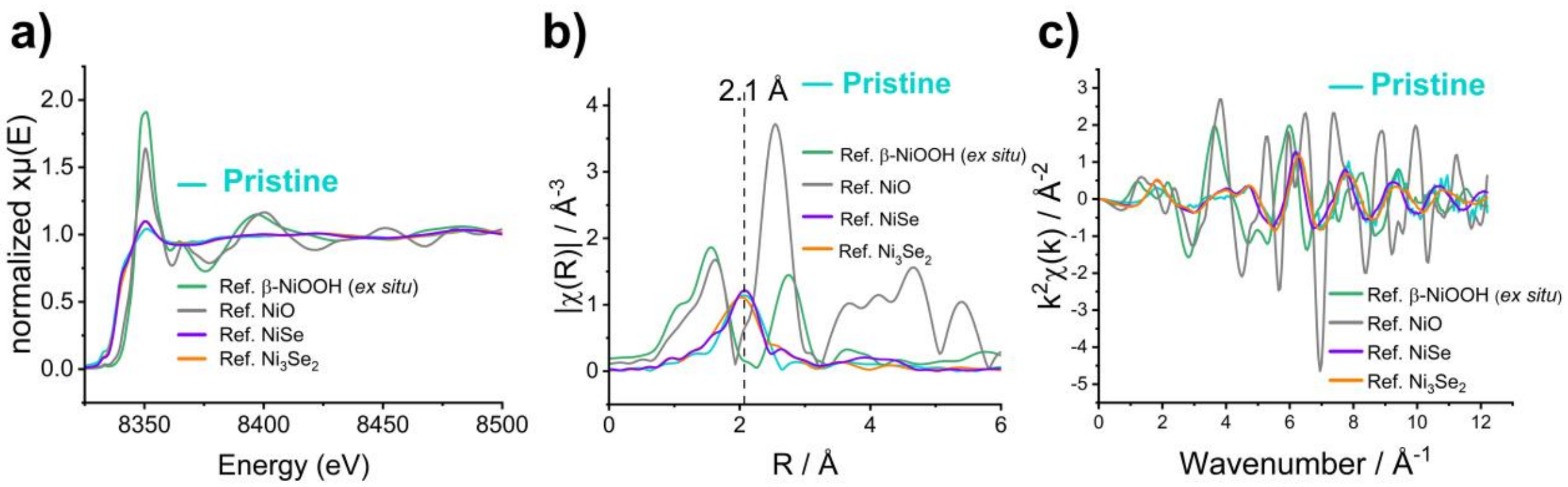

Figure S27. a) *Ex situ* XAS spectra on the Ni K edge showing XANES (8325 – ca. 8375 eV) and EXAFS regions (ca. 8375 – 8500 eV) of pristine NiSe compared to reference materials; b) Fourier-transforms in R-space extracted from EXAFS region in a); c) Fourier-transforms in k-space extracted from EXAFS region in a).

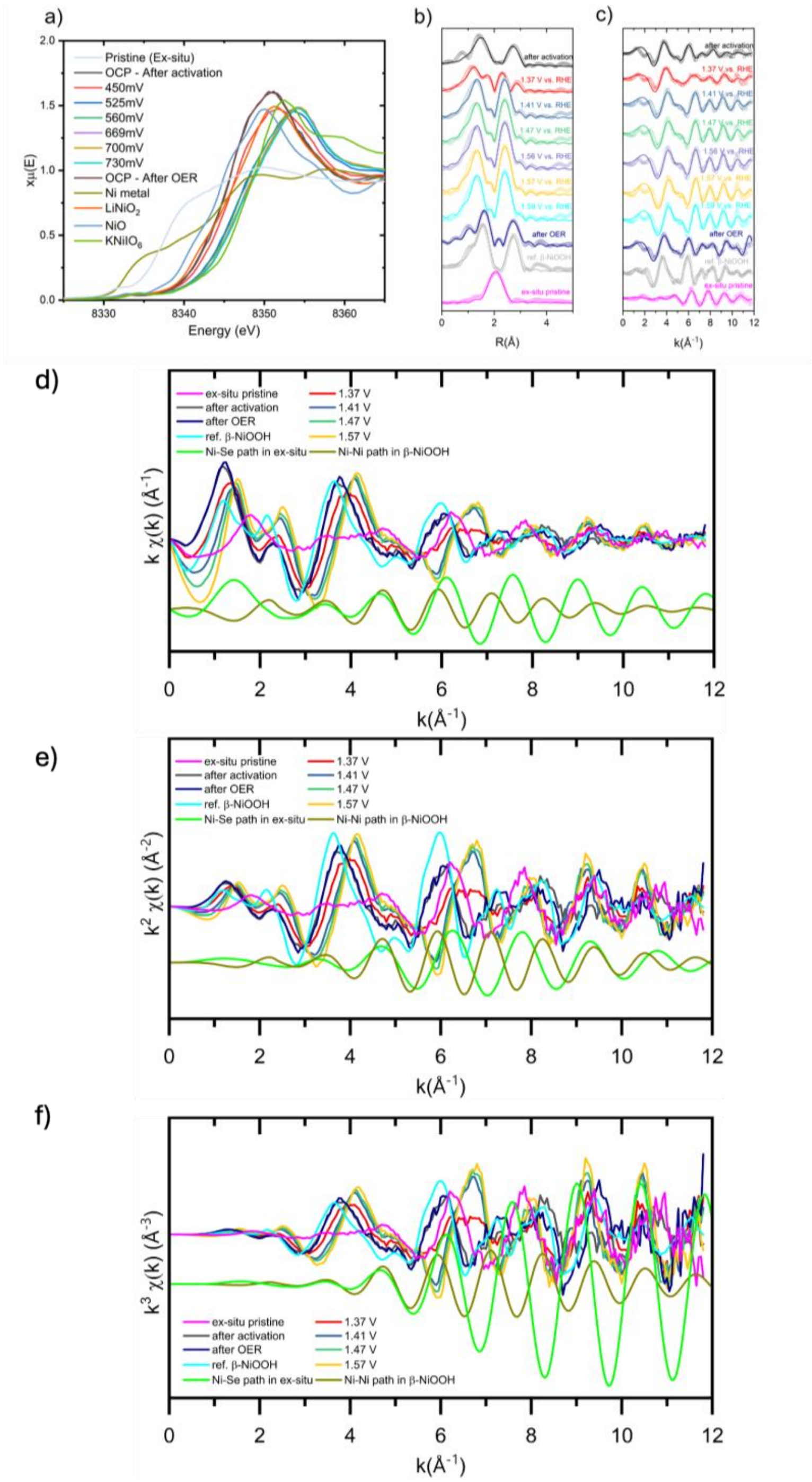

Figure S28. *Operando* XAS characterization at the Ni K-edge: a) XANES spectra, b) Fourier-transformed EXAFS spectra in R-space, and c) fitted k-space spectra displayed with vertical offset. Corresponding EXAFS k-space spectra without vertical offset are shown with k-weightings of d) k^1, e) k^2, and f) k^3 across all investigated potentials and the pristine *ex situ* sample (see Figure S26). The isolated scattering paths labeled "Ni–Se path in ex-situ" and "Ni–Ni path in β-NiOOH" represent individual single-scattering paths extracted from the independent FEFF shell fits of the pristine NiSe and β-NiOOH standards, respectively. The reference spectra of $LiNiO_2$ and $KNiIO_6$ were extracted from previous results of Mansour et al.[37] These isolated paths are included only as qualitative references to illustrate characteristic oscillation frequencies. Therefore, their relative amplitudes should not be interpreted quantitatively.

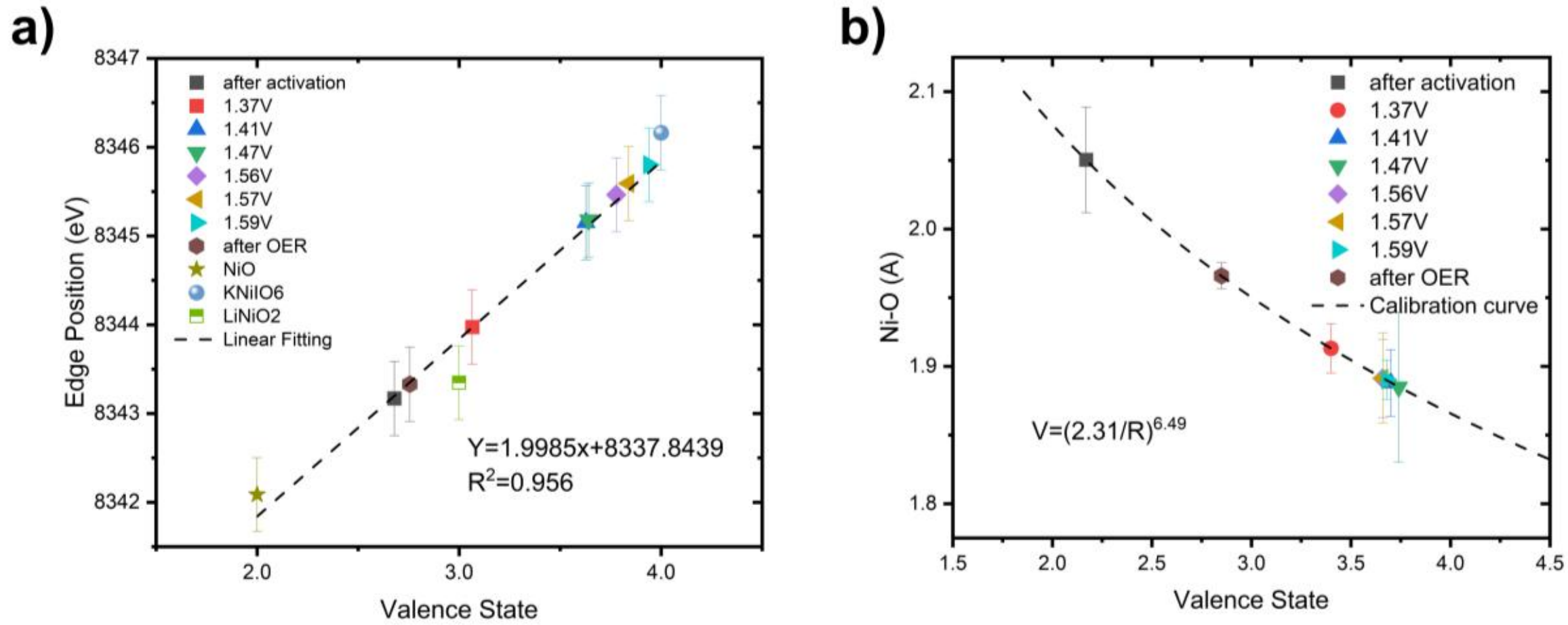

Figure S29. *Operando* XAS fitting to extract the Ni oxidation state via a) integral method from the Ni K-edge position in the XANES region and b) Ni-O calibration curve extracted from the EXAFS region. To obtain the Ni-O calibration curve, the first shell of Ni-O distance was used to identify the Ni oxidation states according to Farley et al.[38] Afterward, the Ni-O calibration curve was obtained following the procedure of Risch et al, and a calibration curve of $V=(2.31/R)^{6.49}$ was obtained.[39] where V is the valence state and R is the Ni-O radial distance.

Table S18. Fitting results of the integral edge position method to extract the Ni oxidation state from the Ni K-edge in XANES region. NiO, $LiNiO_2$, and $KNiIO_6$ were used as standards for Ni in oxidation states 2+, 3+, and 4+, respectively. Error bars of edge position energy were obtained from the beamline energy resolution.

	**Edge Position (eV)**	**Oxidation State**
**OCP- after activation**	8343.17± 0.42	2.7± 0.2
**450mV / 1.37 V vs. RHE**	8343.98± 0.42	3.1± 0.2
**525 mV / 1.41 V vs. RHE**	8345.15± 0.42	3.6± 0.2
**560 mV / 1.47 V vs. RHE**	8345.18± 0.42	3.6± 0.2
**669 mV / 1.56 V vs. RHE**	8345.46± 0.42	3.8± 0.2
**700 mV / 1.57 V vs. RHE**	8345.59± 0.42	3.8± 0.2
**730 mV / 1.59 V vs. RHE**	8345.80± 0.42	3.9± 0.2
**OCP- after OER**	8343.33± 0.42	2.8± 0.2
**NiO**	8342.09	2.0
**$LiNiO_2$**	8343.35	3.0
**$KNiIO_6$**	8346.16	4.0

Table S19. Fitting results of the Ni-O calibration curve to extract Ni-O bond distances and Ni oxidation states from EXAFS regions following the procedures of Farley et al.[38] and Risch et al.[39] A calibration curve of $V=(2.31/R)^{6.49}$ was used, where V is the valence state and R is the Ni-O radial distance, as suggested by Risch et al.[39]

	**Ni-O (Å)**	**Oxidation State**
**OCP- after activation**	2.05± 0.04	2.3± 0.2
**450mV / 1.37 V vs. RHE**	1.91± 0.02	3.0± 0.4
**525 mV / 1.41 V vs. RHE**	1.89± 0.02	3.6± 0.2
**560 mV / 1.47 V vs. RHE**	1.88± 0.05	3.6± 0.2
**669 mV / 1.56 V vs. RHE**	1.89± 0.03	3.7± 0.2
**700 mV / 1.57 V vs. RHE**	1.89± 0.03	3.7± 0.2
**730 mV / 1.59 V vs. RHE**	1.89± 0.01	3.7± 0.2
**OCP- after OER**	1.97± 0.01	2.8± 0.3

Table S20. Structural parameters extracted from the co-refinement of Ni K-edge EXAFS spectra at different applied potentials. Fitting was performed in R-space over a window of R = 1.0 - 3.2 Å (ΔR = 2.2 Å) following a Fourier transform of the k-weighted data from k = 3.0 - 11.5 Å^{-1} (Δk = 8.5 Å^{-1}). Based on the Nyquist criterion, the number of independent data points (N_{idp}) is 11.9. To ensure mathematical determinacy, the model was rigorously restricted to 9 floated parameters via algebraic constraints: the amplitude reduction factor (s_0^2) was fixed, fractional coordination numbers (CN) of the split Ni-O shells were coupled, and energy shifts (ΔE_0) were constrained well within the physically acceptable limit of 10 eV. To further limit free variables, all mean-square disorder/Debye-Waller factors (σ^2) were algebraically coupled to a single floated baseline parameter. Importantly, the radial distance (R) for the primary Ni-Ni1 path was fixed to theoretical values to conserve variables and maintain mathematical determinacy beneath the Nyquist limit, allowing the model to rigorously probe the floated Ni-Se1 parameters. The single-digit numbers in parentheses for radial distance (R) and σ^2 represent the error in the last digit and the numbers in parentheses for CN and E_0 represent the full absolute errors. Values without error parentheses were mathematically fixed or coupled during the fit.

	First Shell										
	Ni-O1 from Ni (2+)			Ni-O2 from Ni (3+)			Ni-O3 from Ni (4+)				
	CN	R(Å)	σ^2(Å^2)	CN	R(Å)	σ^2(Å^2)	CN	R(Å)	σ^2(Å^2)	E_0(eV)	R-factor
After Act.	1.6 (0.9)	2.16 (4)	0.003 (2)	3.3 (1.1)	2.00 (4)	0.005 (4)	-	-	-	-1.9 (0.2)	0.034
1.37V	-	-	-	2.5 (0.3)	2.02 (2)	0.003 (1)	3.3 (0.3)	1.82 (1)	0.005 (1)	-9.4 (2.5)	0.020
1.41V	-	-	-	1.4 (0.8)	2.00 (1)	0.005 (4)	3.6 (0.8)	1.84 (2)	0.005 (4)	-6.7 (1.5)	0.011
1.47V	-	-	-	1.6 (1.4)	2.00 (9)	0.005 (2)	3.6 (1.4)	1.84 (5)	0.005 (2)	-5.4 (1.7)	0.014
1.57V	-	-	-	1.7 (0.9)	2.00 (4)	0.003 (3)	3.3 (0.9)	1.83 (3)	0.003 (3)	-2.9 (2.7)	0.012
After OER	4.0 (0.1)	2.03 (1)	0.009 (1)	1.5 (0.1)	1.80 (2)	0.002 (2)	-	-	-	-7.5 (0.9)	0.004
	Second Shell										
	Ni-Se1			Ni-Ni1			Ni-O5-O6				
	CN	R(Å)	σ^2(Å^2)	CN	R(Å)	σ^2(Å^2)	CN	R(Å)	σ^2(Å^2)	E_0(eV)	R-factor
After Act.	1.2 (0.1)	2.36 (8)	0.014 (7)	5.25 (3.3)	3.10 (2)	0.014 (7)	-	-	-	-1.9 (0.2)	0.034
1.37V	1.8 (0.5)	2.48 (8)	0.014 (6)	1.5 (0.6)	2.78	0.005 (1)	-	3.39 (4)	0.005 (1)	-9.4 (2.5)	0.020
1.41V	1.2 (0.3)	2.49 (7)	0.010 (8)	4.2 (2.0)	2.81	0.007 (4)	-	-	-	-6.7 (1.5)	0.011
1.47V	1.2 (0.3)	2.47 (9)	0.010 (6)	4.3 (1.9)	2.81	0.006 (4)	-	-	-	-5.4 (1.7)	0.014
1.57V	1.2 (0.6)	2.45 (9)	0.011 (3)	4.7 (4.0)	2.82	0.007 (7)	-	-	-	-2.9 (2.7)	0.012
	Ni-Se1			Ni-Ni1			Ni-Ni2				
After OER	1.1 (0.3)	2.57 (5)	0.002 (2)	4.8 (3.1)	2.88	0.01 (1)	3.0 (0.8)	3.08	0.002 (2)	-7.5 (0.9)	0.004

Table S21. Shell Parameter Inter-Correlations, where_delr3o1is the shift in radial distance of Ni-O2 (3+), delr4o1 is the shift in radial distance of Ni-O3 (4+), n3o1 is the coordination number (CN) of Ni-O2 (3+), nni1 is the CN of Ni-Ni1, and ss2 is the mean square disorder of Ni-Ni1. (Note: All other parameter correlations fall below 0.40, indicating excellent stability and a mathematically non-overparameterized fit.)

Shell Parameters	Values
delr4o1 & n3o1	-0.9266
delr3o1 & delr4o1	0.9248
delr3o1 & n3o1	-0.9111
ss2 & nni1	0.8836
delr4o1 & dele	0.4608
delr3o1 & dele	0.4502
delrse1 & delr3o1	-0.4203

Table S22. Structural parameters extracted from the refinement of Ni K-edge EXAFS spectra at different applied potentials when excluding Ni-Se path.

First Shell											
	Ni-O1 from Ni (2+)			Ni-O2 from Ni (3+)			Ni-O3 from Ni (4+)				
	CN	R(Å)	σ^2(Å^2)	CN	R(Å)	σ^2(Å^2)	CN	R(Å)	σ^2(Å^2)	E0(eV)	R-factor
After Act.	1.7 (0.3)	2.12 (1)	0.001 (4)	2.3 (0.6)	1.95 (1)	0.001 (4)	-	-	-	-4.6 (1.4)	0.016
1.37V	-	-	-	5.4 (3.5)	2.00 (4)	0.011 (9)	1.6 (1.6)	1.79 (3)	0.002 (8)	-6.8 (2.7)	0.020
1.41V	-	-	-	1.8 (0.7)	2.01 (6)	0.003 (4)	3.1 (1.1)	1.84 (4)	0.003 (4)	-5.8 (2.7)	0.010
1.47V	-	-	-	2.0 (0.5)	2.01 (3)	0.003 (3)	3.4 (0.3)	1.83 (2)	0.003 (3)	-4.6 (1.8)	0.015
1.57V	-	-	-	1.4 (0.6)	2.03 (3)	0.004 (3)	4.0 (0.8)	1.85 (2)	0.004 (3)	-2.7 (1.9)	0.013
After OER	2.0 (0.3)	2.06 (7)	0.001 (1)	3.5 (0.8)	1.97 (4)	0.02 (1)	-	-	-	-4.2 (1.1)	0.009
**Second Shell**											
	Ni-Ni1			Ni-O4			Ni-O5-O6				
	CN	R(Å)	σ^2(Å^2)	CN	R(Å)	σ^2(Å^2)	CN	R(Å)	σ^2(Å^2)	E0(eV)	R-factor
After Act.	3.4 (0.6)	3.09 (2)	0.013 (3)	2.3 (0.6)	3.44 (4)	0.0001	-	-	-	-4.6 (1.4)	0.016
1.37V	5.4 (3.5)	3.01 (5)	0.018	-	-	-	-	3.07 (0.4)	0.008	-6.8 (2.7)	0.020
1.41V	3.1 (1.1)	2.82 (2)	0.005 (3)	-	-	-	-	-	-	-5.8 (2.7)	0.010
1.47V	3.4 (0.3)	2.83 (1)	0.005 (4)	-	-	-	-	-	-	-4.6 (1.8)	0.015
1.57V	4.0 (0.8)	2.83 (1)	0.005 (2)	-	-	-	-	-	-	-2.7 (1.9)	0.013
	Ni-Ni1			Ni-Ni2							
After OER	4.0 (0.6)	3.09 (1)	0.008 (1)	3.5 (0.8)	2.91 (1)	0.02 (1)				-4.2 (1.1)	0.009

Table S23. In the following table, a direct comparison of structural parameters and fit quality for models with and without Ni-Se is provided. Note the error of the sum of the Ni-O CN is obtained by the square root of the sum of the each error square. Together with the results from *operando* Raman measurements (see Section 3.4) and ICP-MS quantification showing retention of ≈45±2% of the original selenium content after OER (Table S17), we conclude our model including the Ni-Se contribution ("with Ni-Se" in this table) to be more reasonable.

**Potential**	**Model**	**Total Ni-O CN**	**Ni-Se CN**	**R-factor**
OCP after activitation	With Ni-Se	4.9 (1.4)	1.2 (0.1)	0.034
OCP after activitation	Without Ni-Se	4.0 (0.7)	--	0.016
1.37 V	With Ni-Se	5.8 (0.4)	1.8 (0.5)	0.020
1.37 V	Without Ni-Se	7.0 (3.8)	--	0.020
1.41 V	With Ni-Se	5.0 (1.1)	1.2 (0.3)	0.011
1.41 V	Without Ni-Se	4.9 (1.3)	--	0.010
1.47 V	With Ni-Se	5.2 (1.9)	1.2 (0.3)	0.014
1.47 V	Without Ni-Se	5.4 (0.6)	--	0.015
1.57 V	With Ni-Se	5.0 (1.3)	1.2 (0.6)	0.012
1.57 V	Without Ni-Se	5.4 (1.0)	--	0.013
After OER	With Ni-Se	5.5 (0.1)	1.1 (0.3)	0.004
After OER	Without Ni-Se	5.5 (0.9)	--	0.009

2.6 EIS Data

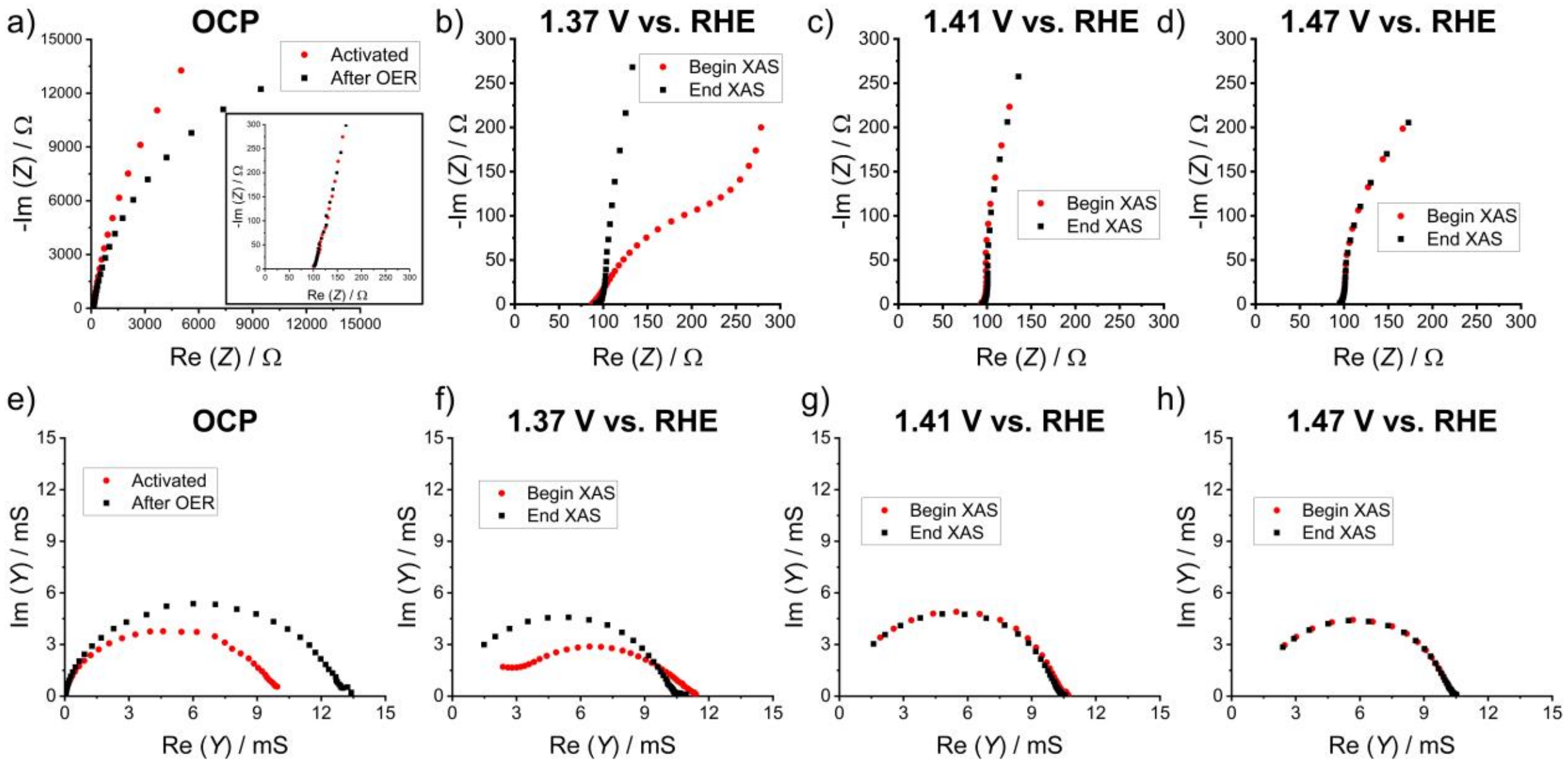

Figure S30. EIS spectra were recorded simultaneously with *operando* XAS investigations and are presented as Nyquist plots in a) – d) for impedance Z and in e) – f) for admittance Y. Potentials are a) and e) OCP (≈0.82 V vs. RHE); b) and f) 1.37 V; c) and g) 1.41 V; d) and h) 1.47 V vs. RHE. For EIS data fitting, the admittance plots were used (see Figure S33).

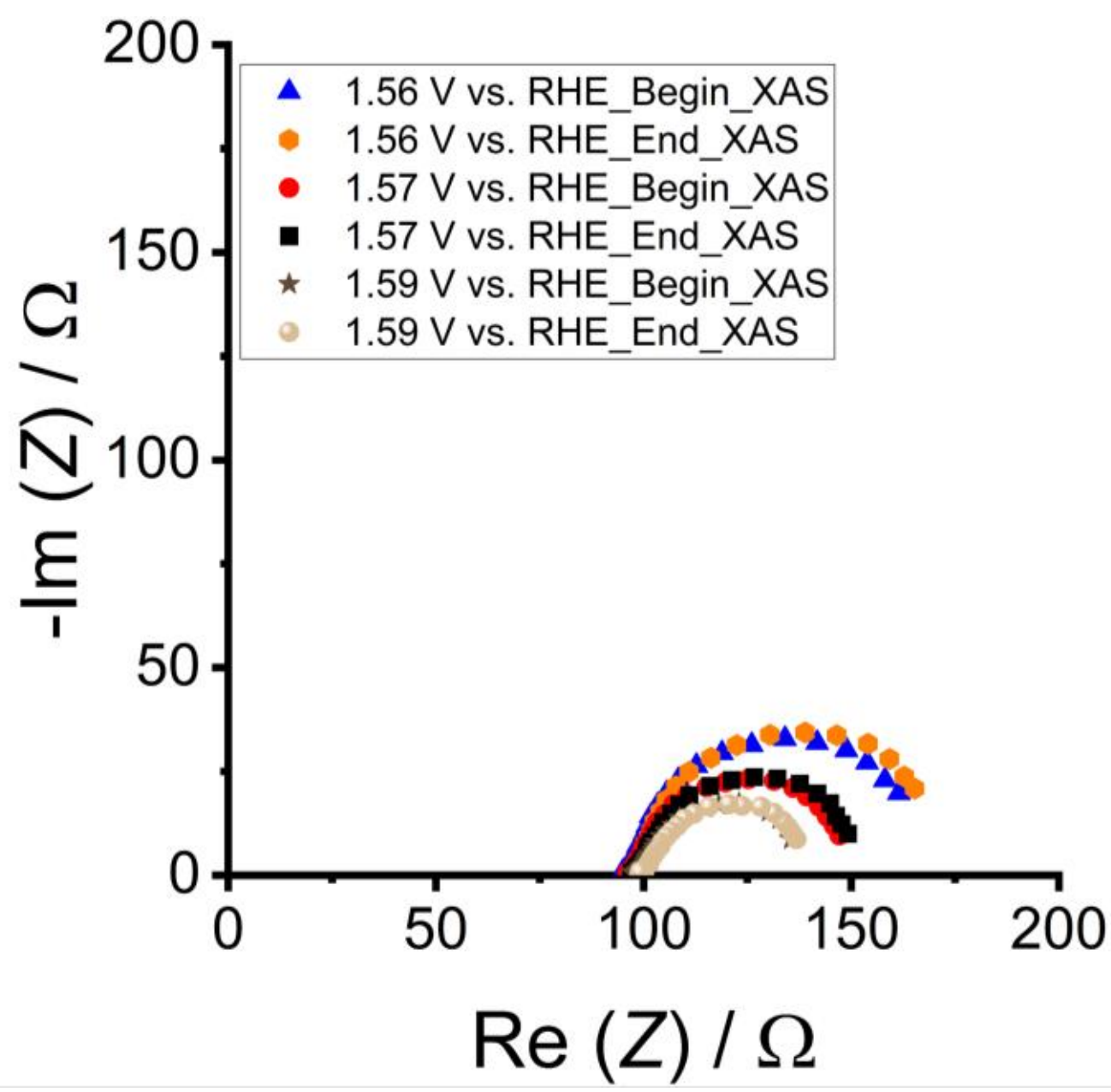

Figure S31. EIS spectra recorded simultaneously with *operando* XAS investigations at OER potentials of 1.56 V, 1.57 V, and 1.59 V vs. RHE. At each potential, one EIS spectrum before and one after the chronoamperometric experiment was conducted as described in SI-1.4.

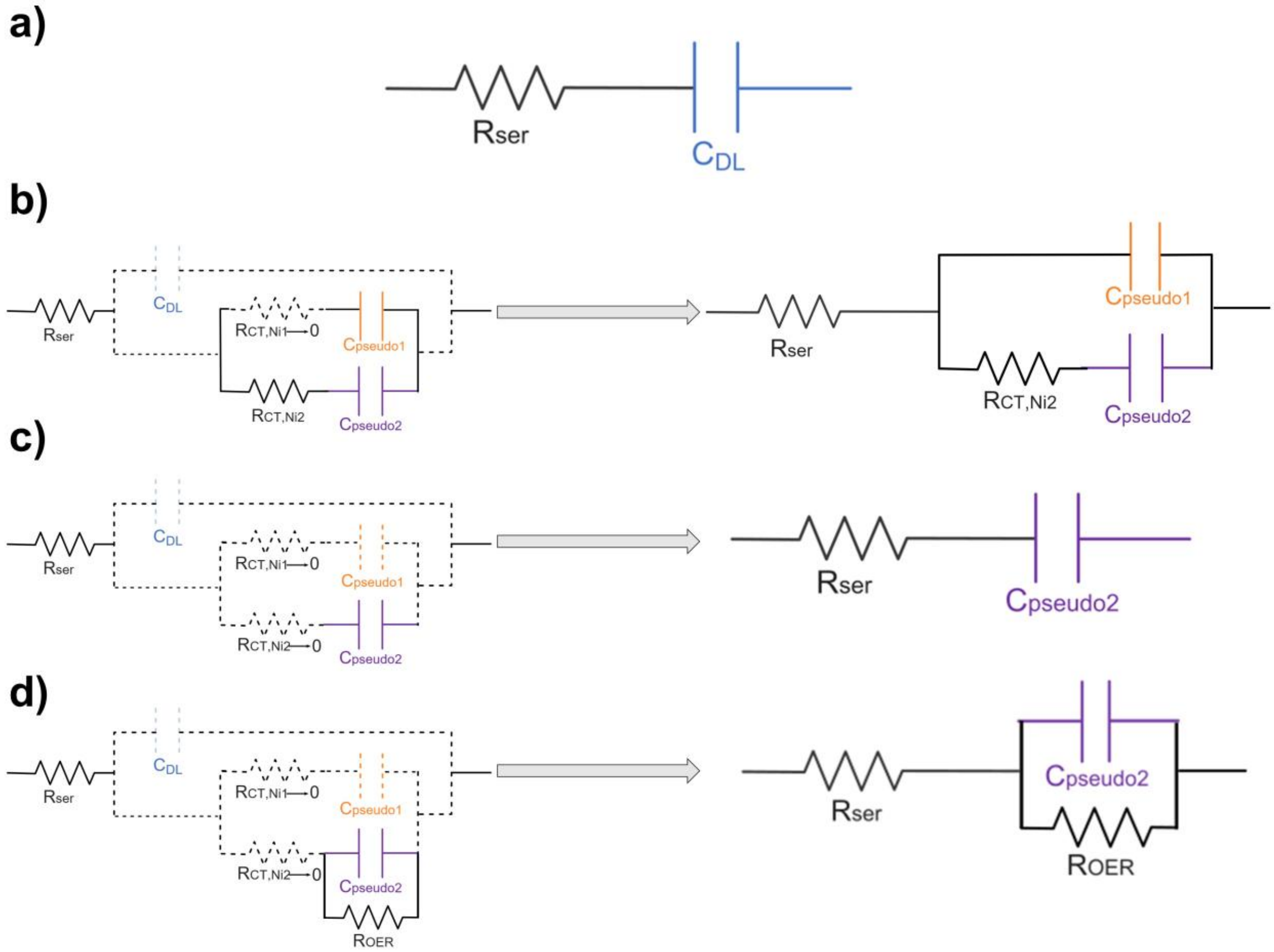

Figure S32. Equivalent circuits used to fit the experimental EIS spectra presented in Figure 4 of the main article at a) OCP (≈0.82 V vs. RHE), b) 1.37 V vs. RHE at the beginning of the XAS scan, c) 1.37 V at the end of the XAS scan as well as 1.41 V and 1.47 V vs. RHE, and d) under OER conditions (1.57 V vs. RHE).

2.6.1 Explanations Regarding EIS Analysis

The EIS spectra at OCP (≈0.82 V vs. RHE) are expected to exhibit only the serial resistance R_{ser} and the double layer capacitance C_{DL}. Thus, the R-C circuit presented in Figure S32a was applied to fit the experimental data.

As described in the main manuscript (Section 3.3), we assume three possible oxidation states of the nickel centers: Ni^{2+} (as would be expected in pristine NiSe), Ni^{3+}, and Ni^{4+}. Facile transitions between oxidation states, herein Ni^{2+}/Ni^{3+} and Ni^{3+}/Ni^{4+}, are expected to result in pseudo-capacitances and charge transfer resistances.[40] Thus, we predict in our fitting model the existence of $R_{CT,Ni1}$ and $C_{pseudo1}$ related to the Ni^{2+} to Ni^{3+} oxidation, and $R_{CT,Ni2}$ and $C_{pseudo2}$ related to the oxidation of Ni^{3+} to Ni^{4+}. As a significant fraction of Ni centers is already in oxidation state +3 at OCP after activation (Table 1 in main manuscript and Table S18 and Table S19), it seems reasonable that $R_{CT,Ni1}$ is negligibly small. At the foot of the Ni oxidation wave (1.37 V vs. RHE) the EIS responses at the beginning and the end of the XAS scan differ as shown in Figure S30 and discussed in the main article (Section 3.3; Figure 4b+c). The EIS spectrum at the beginning of the XAS scan at 1.37 V vs. RHE (red curve in Figure S30b) can be fitted to a circuit containing two R-C elements. Since the C_{DL} can be expected to be negligible in the presence of pseudo-capacitances, and we assume $R_{CT,Ni1}$ to be insignificant as

well, we a constructed a simplified fitting model containing R_{ser}, $C_{pseudo1}$, $C_{pseudo2}$, and $R_{CT,Ni2}$ (Figure S32b). At the end of the XAS scan, the EIS response at 1.37 V vs. RHE (similar to the following pre-OER potentials, 1.41 V and 1.47 V vs. RHE) can be described by only one R-C element. Considering the average Ni oxidation state > +3 determined using XAS (Table 1 in the main manuscript and Table S18 and Table S19), it seems plausible that the remaining elements in the circuit are R_{ser} and $C_{pseudo2}$, leading to the fitting model shown in Figure S32c. Thus, we observe a transient charge transfer resistance for the oxidation of Ni^{3+} to Ni^{4+} at the foot of the Ni oxidation wave (1.37 V vs. RHE).

Under OER conditions, another charge transfer resistance, R_{OER}, related to the rate-limiting step of the OER process, can be expected, leading to the typical Randles circuit in Figure S32d in our fitting model for the catalyst at OER potentials.

The EIS at OER potentials were fitted in impedance plots. However, admittance plots were chosen for data representation of the four pre-OER potentials in Figure S30. Since the admittance is the inverse of impedance, the fitting parameters themselves remain unchanged.[41]

In a complementary fitting approach, the capacitors in the equivalent circuits in Figure S32 were replaced by constant phase elements (CPEs). The results of the fitting models containing CPEs (Table S25, Table S26, andTable S27) are in good agreement with those from the model containing capacitors (Table S24)

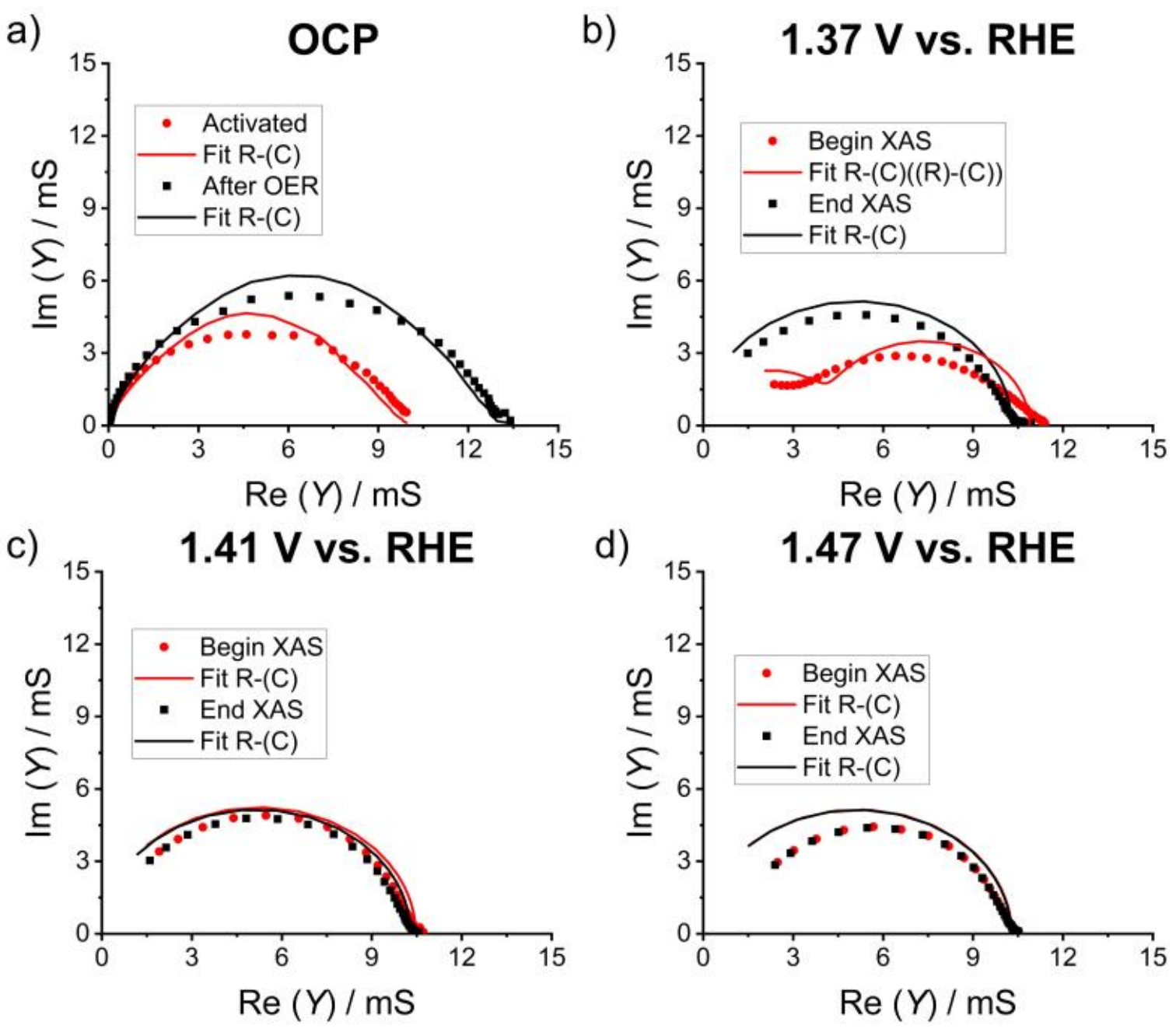

Figure S33. Experimental (symbols) and fitted (lines) EIS spectra recorded simultaneously with *operando* XAS investigations at a) OCP, b) 1.37 V, c) 1.41 V, and d) 1.47 V vs. RHE. All data are presented in admittance plots (see Figure S30). Except for the spectrum before XAS at 1.37 V vs. RHE (b), all spectra could be fitted to an R-C circuit fitting model. At 1.47 V vs. RHE, the fitting becomes less accurate as OER intermediates (e.g., $*OO^-$ from NiOOH deprotonation) might already form on the surface in a significant amount, potentially showing a slight charge transfer resistance. However, for the spectrum before XAS at 1.37 V vs. RHE (b), a fitting model with two R-C elements improved the representation of the experimental EIS data significantly and was selected accordingly.

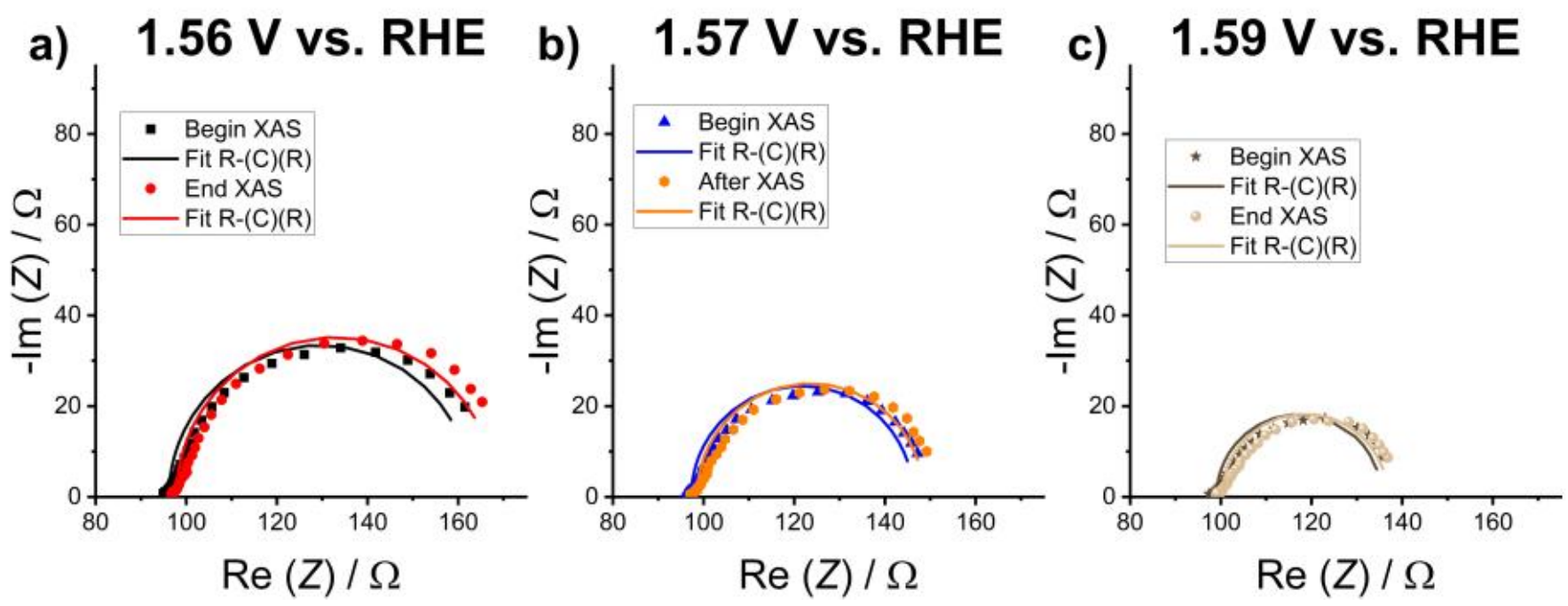

Figure S34. EIS spectra recorded simultaneously with *operando* XAS investigations at a) 1.56 V, b) 1.57 V, and c) 1.59 V vs. RHE, respectively. Fitting to a Randles R-(C)(R) circuit achieved good agreement with the measured EIS data.[42]

Table S24. Summary of the fitting parameters for the EIS data recorded simultaneously with operando XAS investigations and presented in Figure S33 and Figure S34, respectively.

	R_{series} / Ω			C_{DL} / µF cm^{-2}_{geo}			$R_{CT,Ni1}$ / Ω			$C_{pseudo,1}$ / mF cm^{-2}_{geo}			$R_{CT,Ni2}$ / Ω			$C_{pseudo,2}$ / mF cm^{-2}_{geo}			$R_{CT,OER}$ / Ω		
Potential	abs. Value	abs. Error	rel. Error	abs. Value	abs. Error	rel. Error	abs. Value	abs. Error	rel. Error	abs. Value	abs. Error	rel. Error	abs. Value	abs. Error	rel. Error	abs. Value	abs. Error	rel. Error	abs. Value	abs. Error	rel. Error
OCP_after Activation	107.4	1.0	1.0%	75.6	2.0	2.7%															
OCP_after Full Set	108.1	1.0	0.9%	55.0	1.4	2.5%															
1.37 V_1 - Begin XAS	91.8	0.6	0.6%							4.0	0.2	4.1%	164.1	8.5	5.1%	30.6	2.9	9.6%			
1.37 V_2 - End XAS	97.2	0.4	0.5%													27.1	0.5	2.0%			
1.41 V_1 - Begin XAS	95.6	0.2	0.3%													34.3	0.4	1.3%			
1.41 V_2 - End XAS	97.2	0.3	0.3%													29.7	0.4	1.3%			
1.47 V_1 - Begin XAS	97.2	0.4	0.4%													35.7	0.7	2.0%			
1.47 V_2 - End XAS	97.3	0.4	0.4%													34.2	0.7	2.0%			
1.56 V_1 - Begin XAS	96.2	0.2	0.2%													32.6	0.6	1.8%	66.9	1.2	1.7%
1.56 V_2 - End XAS	97.8	0.2	0.2%													30.1	0.5	1.7%	70.4	1.2	1.6%
1.57 V_1 - Begin XAS	97.3	0.2	0.2%													27.0	0.6	2.1%	49.0	0.8	1.6%
1.57 V_2 - End XAS	98.5	0.2	0.2%													27.0	0.6	2.3%	50.1	0.9	1.7%
1.59 V_1 - Begin XAS	99.0	0.2	0.2%													23.8	0.7	2.9%	36.4	0.8	2.2%
1.59 V_2 - End XAS	100.3	0.2	0.2%													24.1	0.7	2.8%	36.6	0.7	1.9%

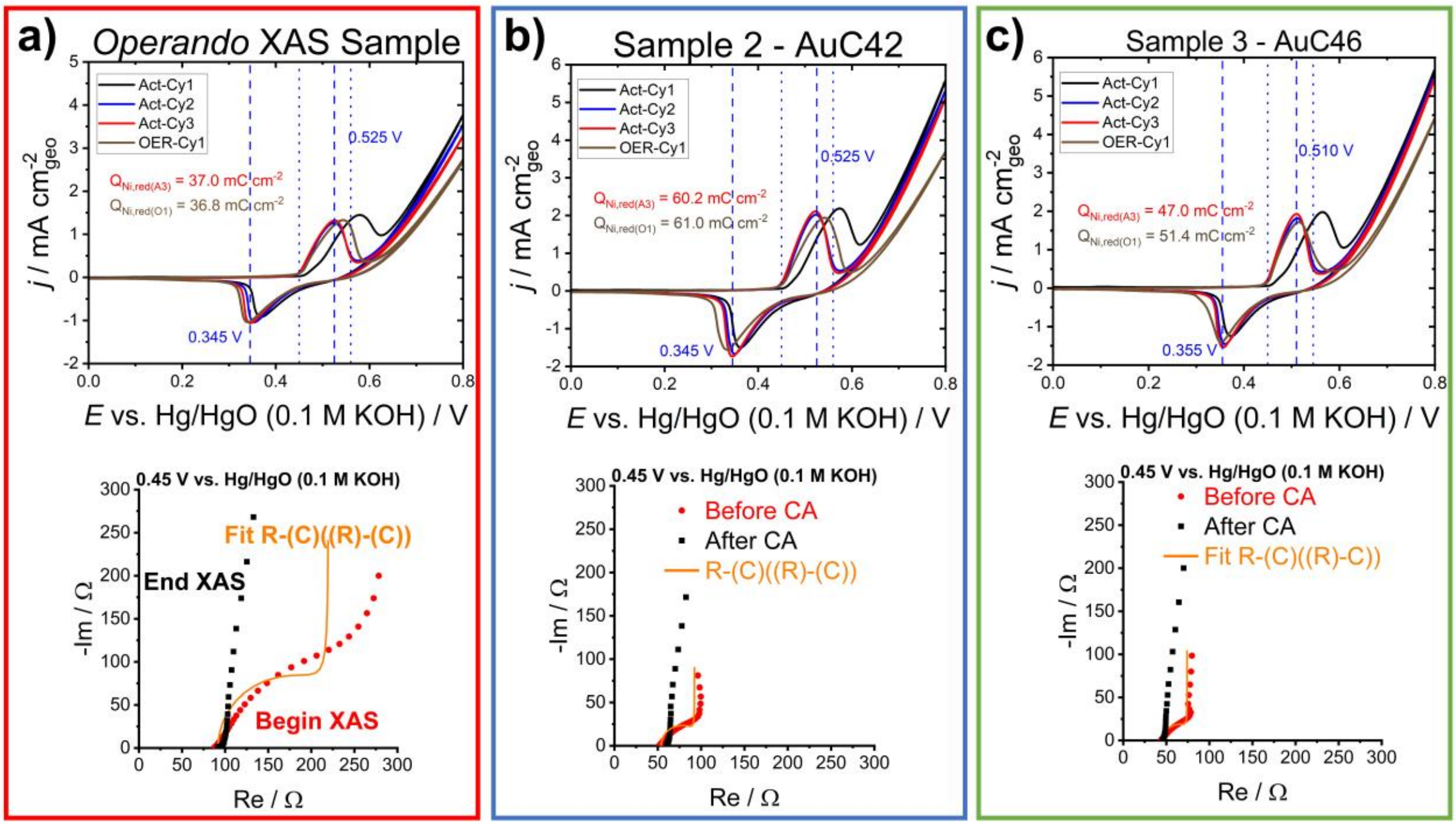

Figure S35. CV cycles and EIS spectra at 0.45 V vs. Hg|HgO|0.1 M KOH (non-*IR* corrected) for three different samples: a) sample used for *operando* XAS investigations, b) and c) two samples investigated electrochemically only. All three samples were deposited as described in SI-1.3 and investigated using the electrochemical protocol presented in SI-1.4. Although the amount of electrochemically active nickel varied, reflected by the charge extracted from Ni reduction peak, the EIS spectra at 0.45 V vs. Hg|HgO|0.1 M KOH (foot of the Ni oxidation peak) before CA (start of XAS scan for beamtime sample in a) differed from the ones after CA (end of XAS scan for beamtime sample in a). Furthermore, the EIS spectra before CA in a), b), and c) show a similar shape but differ in absolute impedance values. However, the three spectra can be fitted using a circuit model with two R-C elements.

Reproducibility of two R-C Elements at 1.37 V at the beginning of XAS scan

The occurrence of two R-C elements in the EIS spectrum before extended conditioning at 1.37 V vs. RHE (beginning of XAS scan for beamtime experiment in Figure S30a and main manuscript), and before CA at 1.37 V vs. RHE for the two samples investigated electrochemically only in Figures S30b+c does not seem to depend only on the experimental timescale. Moreover, the amount of electrochemically active Ni centers seems crucial. The charge of the Ni centers is approximated by the charge density extracted from the Ni-reduction peak (37.0 mC cm^{-2} in the third CV cycle; Figure 3a in the main manuscript). We could not observe this phenomenon of an EIS spectrum with two R-C elements at 1.37 V before CA in any other setup but the *operando* XAS cell (Figure S35). Notably, the charge density of electrochemically active Ni on Au-RDE (Figure S6b) or in the *operando* Raman cell (Figure S7a) was significantly smaller compared to the *operando* XAS cell (ca. 5 to 37 mC cm^{-2}). Likely, as proposed above, a certain number of Ni centers in the $Ni(OH)_2$ state is needed to observe the complexation with hydroxyl ions in EIS. Otherwise, the transformation of $Ni(OH)_2$ to NiOOH species might already not be limited by a significant resistance on the timescale of the experiment anymore.

2.6.2 Reproducibility of the EIS Fitting Using Constant Phase Elements (CPEs)

It is usual in the EIS literature to model imperfect capacitors using constant phase elements (CPEs) to improve the goodness of the fits. Even if their exact physical meaning remains unclear, CPEs can be understood as a distribution of capacitances that depends on the frequency of the input (potential) signal and usually reflects inhomogeneities at the electrode interface.[43,44] The impedance of a CPE can be analytically expressed as follows:

$$Z = \frac{1}{Q(j\omega)^{\alpha}} \qquad \text{(S3)}$$

Where Q is the apparent capacitance (in $Fs^{\alpha-1}$) and α (comprised between 0 and 1) reflects the dispersion of the capacitance with the frequency. When α=1, the CPE is an ideal capacitor. In the following, we have considered the case $\alpha \neq 1$ for each capacitor of the equivalent electrical circuits described in Figure S32, keeping all the other elements of the circuits for each respective fit. The results are summarized in Table S25 for the fits in the OCP region (i.e., modelling C_{DL} as a CPE) and in Table S26 and Table S27 for the other fits (i.e., modelling $C_{pseudo,1}$ and $C_{pseudo,2}$ as CPEs). The effective capacitances have been recalculated based on a mathematical formalism initially developed by Brug et al.[43] The equations (S4) to (S7) were used for the cases described in Figure S27a-d, with α being the CPE dispersion parameter of each capacitance:

$$C_{DL} = (R_{ser})^{\frac{1-\alpha}{\alpha}} (Q_{DL})^{\frac{1}{\alpha}} \qquad \text{(S4)}$$

$$ZC_{pseudo,1} = \left(\frac{1}{R_{ser}} + \frac{1}{R_{CT,\ni 2}}\right)^{\frac{\alpha-1}{\alpha}} \left(Q_{pseudo,1}\right)^{\frac{1}{\alpha}} \qquad \text{(S5)}$$

$$C_{pseudo,2} = (R_{ser})^{\frac{1-\alpha}{\alpha}} \left(Q_{pseudo,2}\right)^{\frac{1}{\alpha}} \qquad \text{(S6)}$$

$$C_{pseudo,2} = \left(\frac{1}{R_{ser}} + \frac{1}{R_{CT,OER}}\right)^{\frac{\alpha-1}{\alpha}} \left(Q_{pseudo,2}\right)^{\frac{1}{\alpha}} \qquad \text{(S7)}$$

The variation between the fit without CPE and the one considering a CPE has been calculated, and the results are given in percentage, relative to the fit without CPE. For the OCP measurements (Table S25), the variation is about 1% on the determination of the double-layer capacitance, indicating a very accurate fit without considering a CPE distribution on C_{DL}. For the measurement at the foot of the Ni oxidation, and before starting the XAS scan (Table S26), a small variation is observed for $C_{pseudo,2}$ (also of about 1%), indicating here again a precise determination of the pseudocapacitance associated with the oxidation of Ni^{3+} to Ni^{4+}. However, a significant variation (of 15%) is observed for $C_{pseudo,1}$, indicating a lack of precision for the determination of the pseudocapacitance associated with the oxidation of Ni^{2+} to Ni^{3+}, as well as for $R_{CT,Ni2}$, for which an accurate determination is not achievable. This also underlines the limitations of the equivalent electrical circuit used in that case. For all other measurements in the Ni oxidation region (Table S27), a good agreement is observed between the two fits ($C_{pseudo,2}$ variation is always less than 6%). In the OER region, under catalytic operation, the determination of $C_{pseudo,2}$ and $R_{CT,OER}$ becomes less precise (variation of about 20%), but still reliable. Overall, we can say that both fitting methods (with or without modelling capacitors

with CPEs) lead to the same values for all parameters within a maximal variation of 20%, validating the equivalent electrical circuits used, except for the one at 1.37 V vs RHE before the XAS scan.

Table S25. Summary of the fitting parameters modelling C_{DL} as a CPE for the EIS data recorded in the OCP region simultaneously with operando XAS investigations (corresponding to the equivalent electrical circuit given in Figure S32a).

	R_{series} / Ω				Q_{DL} * 10^{-6} / $Fs^{\alpha-1}$			α_{DL}			C_{DL} / µF cm^{-2}_{geo}			
Potential	abs. Value	abs. Error	rel. Error	variation	abs. Value	abs. Error	rel. Error	abs. Value	abs. Error	rel. Error	abs. Value	abs. Error	rel. Error	variation
OCP_after Activation	98.2	1.7	1.7%	8.6%	82.1	3.5	4.2%	0.861	0.009	1.1%	75.2	1.2	1.7%	0.5%
OCP_after Full Set	99.7	1.0	1%	7.7%	61.9	1.0	1.6%	0.864	0.004	0.5%	55.6	0.4	1%	1.1%

Table S26. Summary of the fitting parameters modelling $C_{pseudo,1}$ as a CPE and $C_{pseudo,2}$ as an ideal capacitor (to avoid having two CPEs in the same fit) for the EIS data recorded at 1.37 V vs RHE at the beginning of the XAS scan (corresponding to the equivalent electrical circuit given in Figure S32b).

R_{series} / Ω				$Q_{pseudo,1}$ * 10^{-3} / $Fs^{\alpha-1}$			$\alpha_{pseudo,1}$			$C_{pseudo,1}$ / mF cm^{-2}_{geo}				$R_{CT,Ni2}$ / Ω				$C_{pseudo,2}$ / mF cm^{-2}_{geo}			
abs. Value	abs. Error	rel. Error	variation	abs. Value	abs. Error	rel. Error	abs. Value	abs. Error	rel. Error	abs. Value	abs. Error	rel. Error	variation	abs. Value	abs. Error	rel. Error	variation	abs. Value	abs. Error	rel. Error	variation
90.4	0.4	0.4%	1.5%	47.0	2.6	5.6%	0.607	0.018	3%	4.6	0.24	5.3	15%	312	25	8.1%	90%	30.2	1.8	6.0%	1.3%

Table S27. Summary of the fitting parameters modelling $C_{pseudo,2}$ as a CPE and $C_{pseudo,1}$ as an ideal capacitor for the EIS data recorded at 1.37 V vs RHE at the end of the XAS scan and all following measurements at higher potentials (corresponding to the equivalent electrical circuits given in Figure S32c and d).

	R_{series} / Ω				$Q_{pseudo,2}$ * 10^{-3} / $Fs^{\alpha-1}$			$\alpha_{pseudo,2}$			$C_{pseudo,2}$ / mF cm^{-2}_{geo}				$R_{CT,OER}$ / Ω			
Potential	abs. Value	abs. Error	rel. Error	variation	abs. Value	abs. Error	rel. Error	abs. Value	abs. Error	rel. Error	abs. Value	abs. Error	rel. Error	variation	abs. Value	abs. Error	rel. Error	variation
1.37 V_2 - End XAS	93.9	1.4	1.5%	3.4%	13.2	0.1	0.9%	0.963	0.029	3.0%	27.2	0.4	1.5%	0.4%				
1.41 V_1 - Begin XAS	95.0	1.4	1.5%	0.6%	16.4	0.1	0.5%	0.898	0.021	2.3%	34.5	0.5	1.5%	0.6%				
1.41 V_2 - End XAS	95.1	1.1	1.2%	2.2%	14.5	0.1	0.7%	0.889	0.021	2.4%	30.2	0.4	1.4%	1.7%				
1.47 V_1 - Begin XAS	96.6	1.1	1.1%	0.6%	17.4	0.1	0.4%	0.854	0.019	2.2%	38.0	0.5	1.7%	6.4%				
1.47 V_2 - End XAS	97.0	1.0	1.0%	0.3%	16.7	0.1	0.4%	0.854	0.019	2.0%	36.2	0.5	1.4%	5.8%				
1.56 V_1 - Begin XAS	98.3	0.3	0.3%	2.2%	14.6	0.1	0.5%	0.873	0.030	3.4%	26.4	0.4	1.7%	19.0%	53.0	0.6	1.1%	20.8%
1.56 V_2 - End XAS	99.0	0.4	0.4%	1.2%	14.7	0.1	0.8%	0.861	0.028	3.2%	26.4	0.6	2.1%	12.3%	55.0	0.6	1.4%	21.9%

2.7 Raman Data

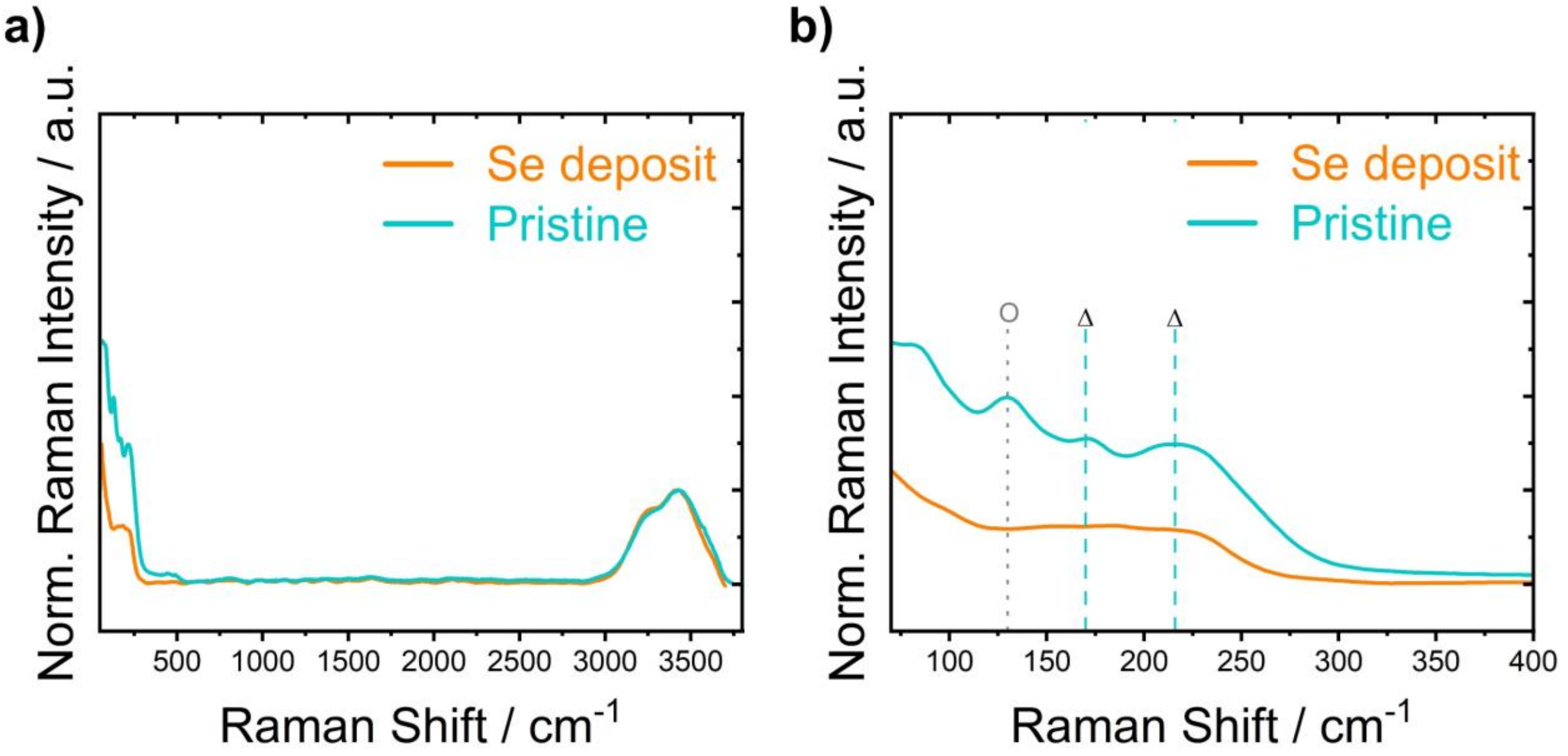

Figure S36. *Operando* Raman spectra of pristine nickel selenide and pure selenium electrodeposits in 100 mM KOH + 1 mM $Fe(NO_3)_3$ at OCP measured during 5 min. The spectra are presented as the mean of the spectra during the experiment; b) Expansion of the low-wavenumber region. As expected, the bands at 167 cm^{-1} and 216 cm^{-1}, which are related in literature reports to E_g (167 cm^{-1}) and A_g (216 cm^{-1}) vibrations in NiSe,[45–47] are absent in the pure Se deposit. However, the band at 129 cm^{-1} related to chain deformations in SeO_2[48] is only present in the pristine NiSe deposit (marked with O in b).[48]

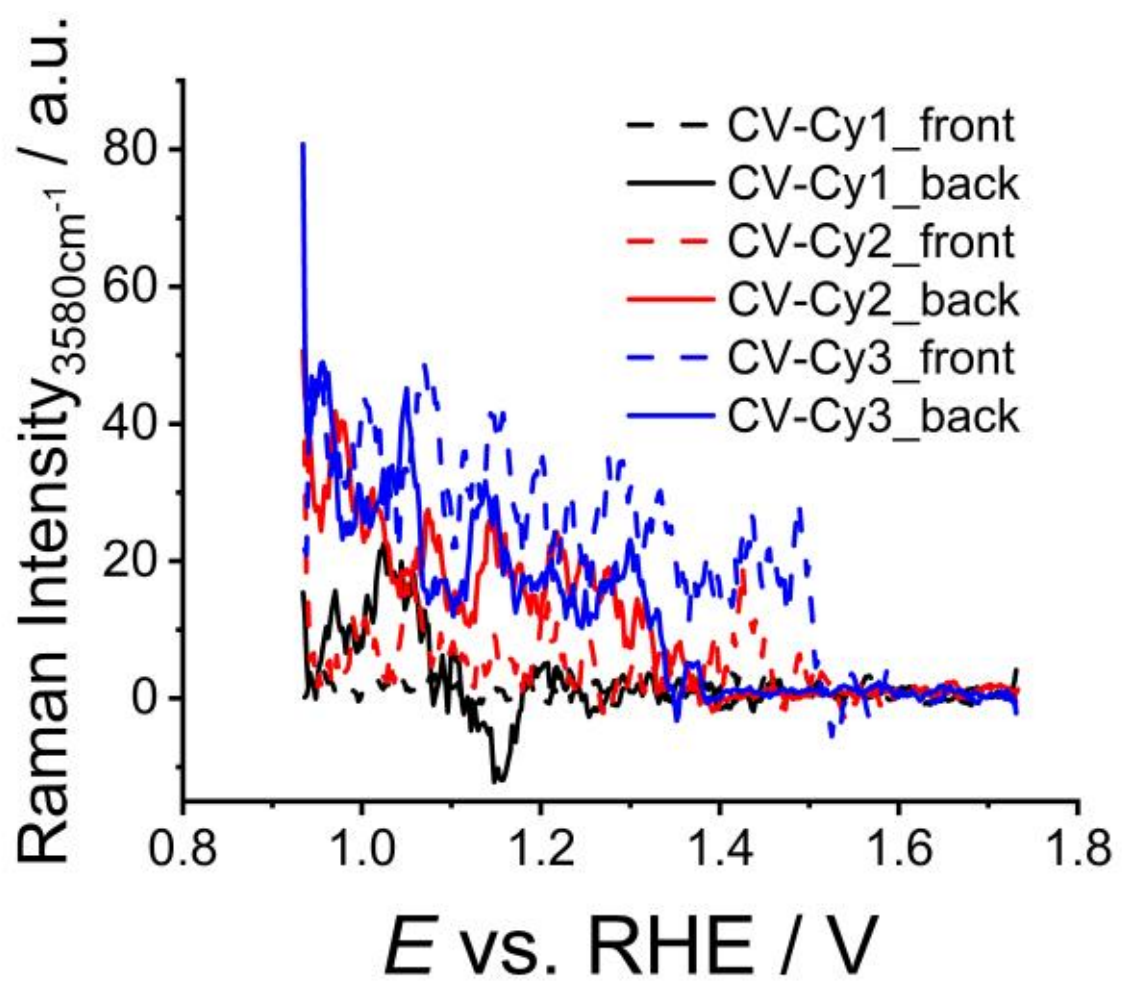

Figure S37. Raman intensity of the band at 3580 cm^{-1} related to OH-stretching in β-$Ni(OH)_2$[47] plotted against the applied potential for three CV cycles during the activation period. Notably, the intensity of the band at 3580 cm^{-1} increased with ongoing cycling, especially at potentials cathodic to (= lower than) the Ni reduction peak (1.35 V vs. RHE, see Figure S7).

2.8 DEMS Data

During the experimental sequence described in SI-1.8 (CVs in $H_2{}^{18}O$-rich electrolyte, electrolyte exchange, CVs in $H_2{}^{18}O$-lean electrolytes), the mass spectrometric response for masses 32 (i.e., $^{16}O_2$) and 34 (i.e., $^{16}O^{18}O$) was monitored continuously. This is exemplified in Figure S38, which displays the raw data from one experiment. Figure S38a (top) shows the faradaic current measured by the potentiostat as a function of time, whereas Figure S38a (middle and bottom) show the ionic current (i.e., mass spectrometric response) for masses 32 and 34. Since data acquisition for the mass spectrometer and potentiostat were started at different times, both data sets are shifted on the time axis. To facilitate data assignment, we have colored the time domain red, in which the CVs were recorded in the $H_2{}^{18}O$-rich electrolyte. Blue indicates the time domain of the electrolyte exchange, and green indicates the time domain in which the CVs were recorded in the $H_2{}^{18}O$-lean electrolyte. Figure S38b shows an enlargement of the mass spectrometric response for mass 34 in the green time domain, which shows that the signal for mass 34 is observed during oxygen evolution.

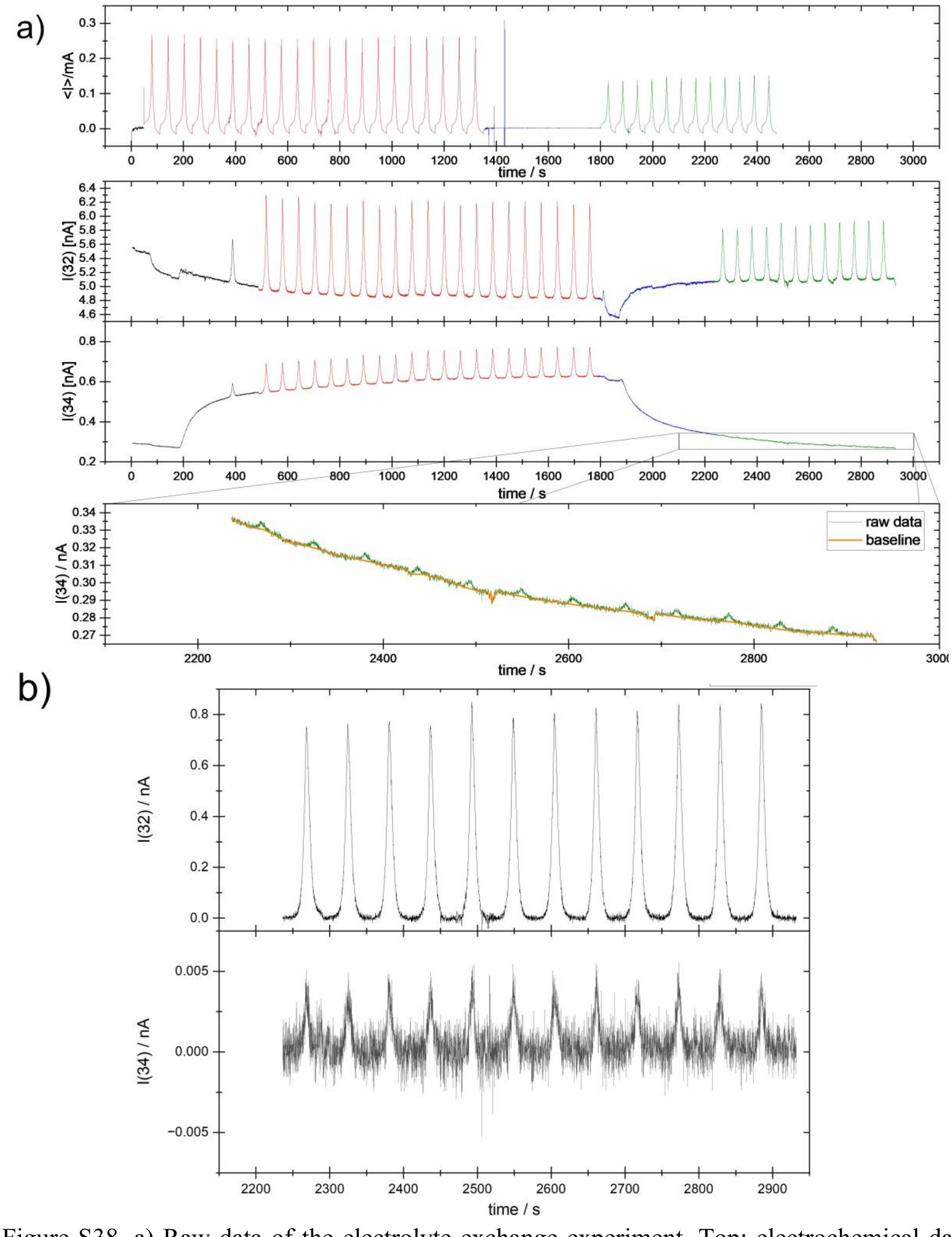

Figure S38. a) Raw data of the electrolyte exchange experiment. Top: electrochemical data; middle: ionic current for masses 32 and 34; bottom: ionic current for mass 34 after the exchange to the $H_2{}^{18}O$-lean electrolyte has been conducted (green) and baseline (orange). Colors indicate time regimes: black: start-up phase; exchange to the $H_2{}^{18}O$-rich electrolyte; red: cycling in $H_2{}^{18}O$-rich electrolyte; blue: exchange to the $H_2{}^{18}O$-lean electrolyte; green: cycling in $H_2{}^{18}O$-lean electrolyte; b) baseline corrected ionic currents for mass 32 (top) and 34 (bottom) obtained during cycling in the $H_2{}^{18}O$-rich electrolyte.

For data evaluation, only the data obtained after the electrolyte exchange are considered. The mass spectrometric data were baseline-corrected. The bottom plot of Figure S38a shows the

baseline (orange), Figure S38b shows baseline-corrected data. After baseline correction, the signals in the ionic current for mass 32 and 34 due to each scan into the OER-region were integrated. This yields an ionic charge ($Q_I(32)$ and $Q_I(34)$) for each cycle that can be transformed into a mol fraction $\chi(^{16}O^{18}O)$ for each scan according to Equation (S8):

$$\chi(^{16}O^{18}O)=\frac{Q_I(34)}{Q_I(32)+Q_I(34)} \quad \text{(S8)}$$

The implicit assumption of Equation (S8) is that the mass spectrometer features the same sensitivity for $^{16}O^{18}O$ and $^{16}O_2$, which is reasonable as the sensitivity difference of the mass spectrometer for the same molecules with similar mass is very similar.[49] In Figure S39a, b, and c the $\chi(^{16}O^{18}O)$ values determined for three distinct electrolyte exchange experiments are plotted versus the charge passed in the OER region (Q(OER)). The latter was determined by integrating each CV over the entire cycle, which should cancel any current contribution due to (pseudo)capacitive processes. As outlined previously,[50] $\chi(^{16}O^{18}O)$ should decline exponentially as a function of Q(OER) if the oxygen evolution proceeded via the lattice oxygen exchange mechanism. This is shown in the simulated data (red dots and blue triangles) calculated via the equation system derived elsewhere.[50] For these simulations, we assume that 1.53 nmol of lattice oxygen atoms exist in the surface of the nickel catalyst. For a surface area of 0.283 cm^2 and for a typical roughness factor of 1.17 for this catalyst. This corresponds to a single monolayer of catalyst. In the first simulation, it is assumed that all these oxygen atoms participate in the lattice oxygen exchange mechanism (red circles) and that this is the only pathway of electrochemical oxygen evolution. In the second simulation, we assume that only a quarter of the surface oxygen atoms participate in the lattice exchange mechanism and that only 10% of the oxygen is evolved via this pathway. Note that simulations were done for the Q(OER)-values that were observed experimentally. Since $\chi(^{16}O^{18}O)$ for a given cycle changes with the charge transferred in that cycle, the simulated $\chi(^{16}O^{18}O)$ values also change (in particular the first one).

The simulations in Figure S39a, b, and c highlight that we would be able to observe the lattice oxygen exchange mechanism in the experimental data through an exponential decline of the $\chi(^{16}O^{18}O)$, even if only a fraction of the lattice surface atoms participated in the lattice oxygen exchange mechanism and if only a fraction of the oxygen is evolved via this pathway. However, our experimentally determined $\chi(^{16}O^{18}O)$-values do not decrease at all when the passed faradaic charge increases. Already, the first $\chi(^{16}O^{18}O)$-value is 0.4% in each experiment, which corresponds to the value expected from natural isotope abundance. Accordingly, our data show that no or a negligible fraction of oxygen is evolved via the oxygen evolution pathway.

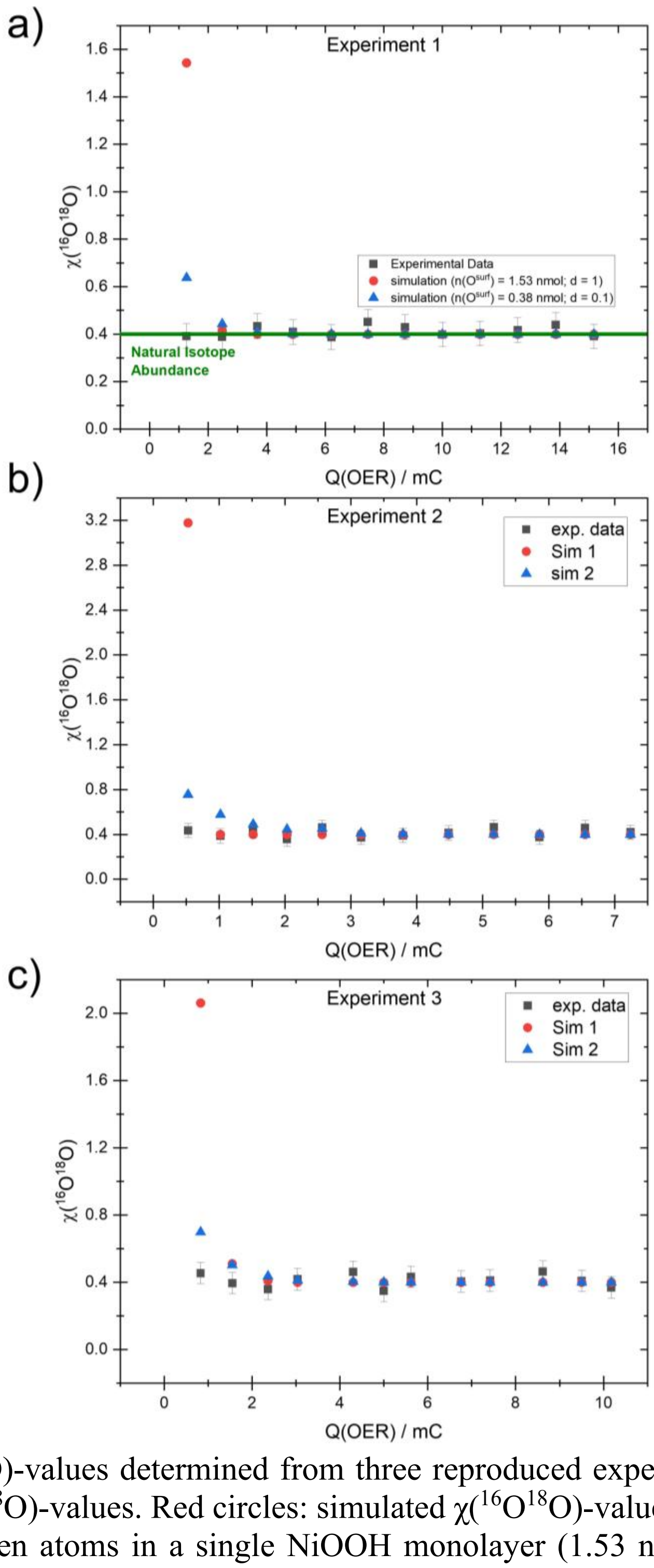

Figure S39. $\chi(^{16}O^{18}O)$-values determined from three reproduced experiments (black squares) and simulated $\chi(^{16}O^{18}O)$-values. Red circles: simulated $\chi(^{16}O^{18}O)$-values under the assumption that all surface oxygen atoms in a single NiOOH monolayer (1.53 nmol; A_{geo} = 0.283 cm^2; roughness factor: 1.17) participated in the lattice oxygen exchange mechanism and that this is the only pathway of oxygen evolution. Blue triangles: simulated $\chi(^{16}O^{18}O)$-values under the assumption that only ¼ of all lattice atoms participate in the lattice oxygen exchange mechanism and that only 10% of the total oxygen formed is evolved via this pathway.

2.9 SFC-ICP-MS Data

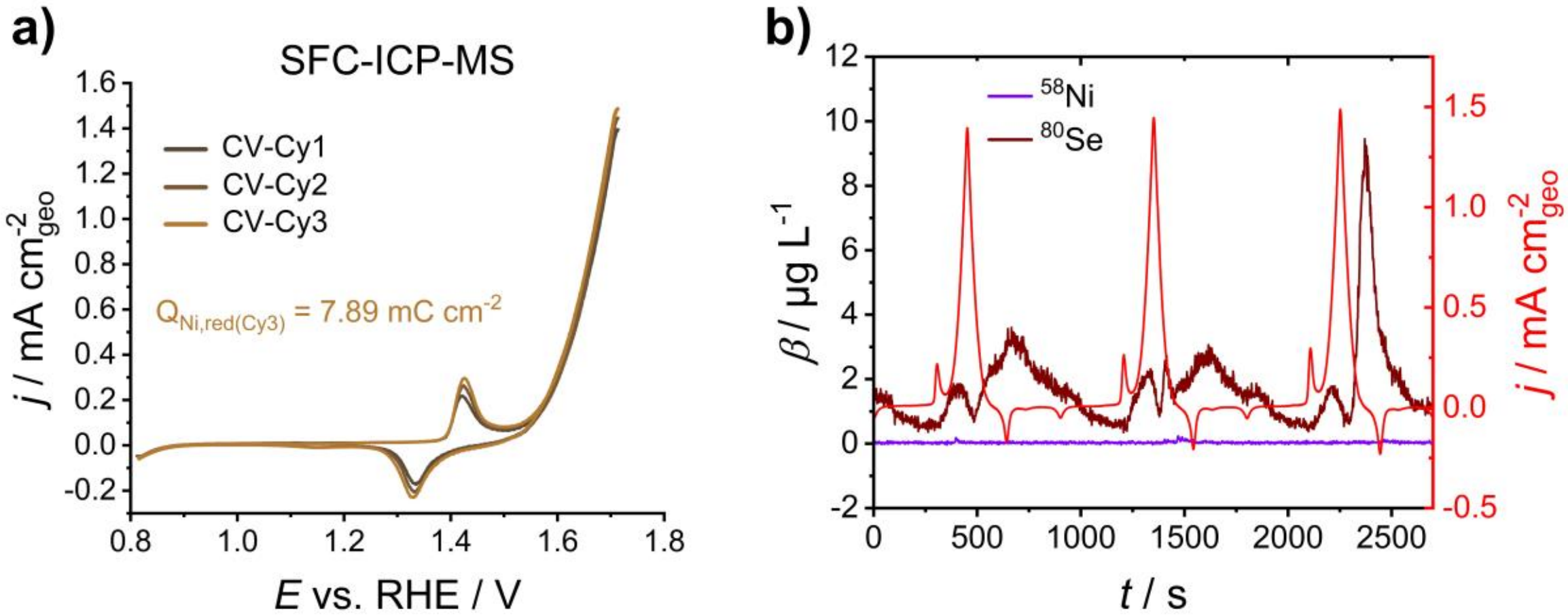

Figure S40. SFC-ICP-MS investigations on Au-modified carbon paper with electrodeposited nickel selenide according to SI-1.3. Experimental details of the SFC-ICP-MS measurement procedure are described in SI-1.9. a) Three CV curves resulting from the activation period (see protocol in SI-1.4); b) mass concentration of ^{58}Ni and ^{80}Se detected in the electrolyte during CV-cycling against the timescale of the experiment. The red curve is the current response of the three consecutive CV curves (right ordinate). The amount of detected ^{58}Ni (blue line, left ordinate) is negligible, indicating that no Ni is leached out under *operando* conditions. In contrast to this, significant amounts of ^{80}Se are detected in the electrolyte during cycling. Under OER conditions (high anodic current densities), the ^{80}Se concentration increased first but decreased again when the OER potential region was passed on the back scan of each CV cycle. Notably, the ^{80}Se concentration increased even further in the region of the Ni reduction peak (highest cathodic current response). The highest Se concentration was detected in the third CV cycle. These data indicate that Se is especially leached out during nickel reduction.

References

(1) Oesch, U.; Janata, J. Electrochemical Study of Gold Electrodes with Anodic Oxide Films—II. Inhibition of Electrochemical Redox Reactions by Monolayers of Surface Oxides. *Electrochim. Acta* **1983**, *28* (9), 1247–1253. DOI: 10.1016/0013-4686(83)85012-9.

(2) Cao, X.; Hong, Y.; Zhang, N.; Chen, Q.; Masud, J.; Zaeem, M. A.; Nath, M. Phase Exploration and Identification of Multinary Transition-Metal Selenides as High-Efficiency Oxygen Evolution Electrocatalysts through Combinatorial Electrodeposition. *ACS Catal.* **2018**, *8* (9), 8273–8289. DOI: 10.1021/acscatal.8b01977.

(3) Wang, M.; Árnadóttir, L.; Xu, Z. J.; Feng, Z. In Situ X-ray Absorption Spectroscopy Studies of Nanoscale Electrocatalysts. *Nanomicro. Lett.* **2019**, *11* (1), 47. DOI: 10.1007/s40820-019-0277-x.

(4) Anantharaj, S.; Kundu, S.; Noda, S. "The Fe Effect": A Review Unveiling the Critical Roles of Fe in Enhancing OER Activity of Ni and Co Based Catalysts. *Nano Energy* **2021**, *80*, 105514. DOI: 10.1016/j.nanoen.2020.105514.

(5) Hiege, F.; Chang, C.-W.; Trost, O.; van Halteren, C. E. R.; Hosseini, P.; Bendt, G.; Schulz, S.; Feng, Z.; Linnemann, J.; Tschulik, K. Morphological Degradation of Oxygen Evolution Reaction-Electrocatalyzing Nickel Selenides at Industrially Relevant Current Densities. *ACS Appl. Mater. Interfaces* **2025**, *17* (29), 41893–41903. DOI: 10.1021/acsami.5c05381.

(6) Bard, A. J.; Faulkner, L. R.; White, H. S. *Electrochemical Methods*:*Fundamentals and Applications*, Third Edition; Wiley, 2022.

(7) Ravel, B.; Newville, M. ATHENA, ARTEMIS, HEPHAESTUS: Data Analysis for X-ray Absorption Spectroscopy Using IFEFFIT. *J. Synchrotron Radiat.* **2005**, *12* (4), 537–541. DOI: 10.1107/S0909049505012719.
(8) Cai, Z.; Zhou, D.; Wang, M.; Bak, S.-M.; Wu, Y.; Wu, Z.; Tian, Y.; Xiong, X.; Li, Y.; Liu, W.; Siahrostami, S.; Kuang, Y.; Yang, X.-Q.; Duan, H.; Feng, Z.; Wang, H.; Sun, X. Introducing Fe^{2+} into Nickel–Iron Layered Double Hydroxide: Local Structure Modulated Water Oxidation Activity. *Angew. Chem.* **2018**, *130* (30), 9536–9540. DOI: 10.1002/ange.201804881.
(9) Fairley, N.; Fernandez, V.; Richard-Plouet, M.; Guillot-Deudon, C.; Walton, J.; Smith, E.; Flahaut, D.; Greiner, M.; Biesinger, M.; Tougaard, S.; Morgan, D.; Baltrusaitis, J. Systematic and Collaborative Approach to Problem Solving Using X-ray Photoelectron Spectroscopy. *Appl. Surf. Sci. Adv.* **2021**, *5*, 100112. DOI: 10.1016/j.apsadv.2021.100112.
(10) Morgan, D. J. The Utility of Adventitious Carbon for Charge Correction: A Perspective From a Second Multiuser Facility. *SIA* **2025**, *57* (1), 28–35. DOI: 10.1002/sia.7360.
(11) Biesinger, M. C.; Payne, B. P.; Lau, L. W. M.; Gerson, A.; St. Smart, R. C. X-ray Photoelectron Spectroscopic Chemical State Quantification of Mixed Nickel Metal, Oxide and Hydroxide Systems. *SIA* **2009**, *41* (4), 324–332. DOI: 10.1002/sia.3026.
(12) Barr, T. L.; Seal, S. Nature of the Use of Adventitious Carbon as a Binding Energy Standard. *J. Vac. Sci. Technol. A* **1995**, *13* (3), 1239–1246. DOI: 10.1116/1.579868.
(13) Trotochaud, L.; Young, S. L.; Ranney, J. K.; Boettcher, S. W. Nickel-Iron Oxyhydroxide Oxygen-Evolution Electrocatalysts: The Role of Intentional and Incidental Iron Incorporation. *J. Am. Chem. Soc.* **2014**, *136* (18), 6744–6753. DOI: 10.1021/ja502379c.
(14) Zhang, S.; Scheu, C. Evaluation of EELS Spectrum Imaging Data by Spectral Components and Factors from Multivariate Analysis. *Microscopy (Oxf.)* **2018**, *67* (suppl_1), i133-i141. DOI: 10.1093/jmicro/dfx091.
(15) Jusys, Z.; Massong, H.; Baltruschat, H. A New Approach for Simultaneous DEMS and EQCM: Electro-oxidation of Adsorbed CO on Pt and Pt-Ru. *J. Electrochem. Soc.* **1999**, *146* (3), 1093–1098. DOI: 10.1149/1.1391726.
(16) Bondue, C. J.; Koper, M. T. A DEMS approach for the direct detection of CO formed during electrochemical CO2 reduction. *J. Electroanal. Chem.* **2020**, *875*, 113842. DOI: 10.1016/j.jelechem.2020.113842.
(17) Wolter, O.; Heitbaum, J. Differential Electrochemical Mass Spectroscopy (DEMS) — a New Method for the Study of Electrode Processes. *Ber. Bunsenges. Phys. Chem.* **1984**, *88* (1), 2–6. DOI: 10.1002/bbpc.19840880103.
(18) Klemm, S. O.; Topalov, A. A.; Laska, C. A.; Mayrhofer, K. J. Coupling of a High Throughput Microelectrochemical Cell with Online Multielemental Trace Analysis by ICP-MS. *Electrochem. Commun.* **2011**, *13* (12), 1533–1535. DOI: 10.1016/j.elecom.2011.10.017.
(19) Topalov, A. A.; Cherevko, S.; Zeradjanin, A. R.; Meier, J. C.; Katsounaros, I.; Mayrhofer, K. J. J. Towards a Comprehensive Understanding of Platinum Dissolution in Acidic Media. *Chem. Sci.* **2014**, *5* (2), 631–638. DOI: 10.1039/C3SC52411F.
(20) Jenewein, K. J.; Kormányos, A.; Knöppel, J.; Mayrhofer, K. J. J.; Cherevko, S. Accessing *In Situ* Photocorrosion under Realistic Light Conditions: Photoelectrochemical Scanning Flow Cell Coupled to Online ICP-MS. *ACS Meas. Sci. Au* **2021**, *1* (2), 74–81. DOI: 10.1021/acsmeasuresciau.1c00016.
(21) Dellis, J.-L. *ZfitGUI 1.0.4*, 2020. https://www.mathworks.com/matlabcentral/fileexchange/69811-zfitgui (accessed 2025-08-06).
(22) Oertzen, G. U. von; Harmer, S. L.; Skinner, W. M. XPS and Ab Initio Calculation of Surface States of Sulfide Minerals: Pyrite, Chalcopyrite and Molybdenite. *Mol. Simul.* **2006**, *32* (15), 1207–1212. DOI: 10.1080/08927020601081616.

(23) Zhu, J.; Ni, Y. Phase-Controlled Synthesis and the Phase-Dependent HER and OER Performances of Nickel Selenide Nanosheets Prepared by an Electrochemical Deposition Route. *CrystEngComm* **2018**, *20* (24), 3344–3352. DOI: 10.1039/C8CE00381E.
(24) Yuan, B.; Luan, W.; Tu, S. One-Step Solvothermal Synthesis of Nickel Selenide Series: Composition and Morphology Control. *CrystEngComm* **2012**, *14* (6), 2145. DOI: 10.1039/c2ce06474j.
(25) Biesinger, M. C.; Payne, B. P.; Grosvenor, A. P.; Lau, L. W.; Gerson, A. R.; Smart, R. S. Resolving Surface Chemical States in XPS Analysis of First Row Transition Metals, Oxides and Hydroxides: Cr, Mn, Fe, Co and Ni. *Appl. Surf. Sci.* **2011**, *257* (7), 2717–2730. DOI: 10.1016/j.apsusc.2010.10.051.
(26) Mansour, A. N.; Melendres, C. A. Characterization of α -$Ni(OH)_2$ by XPS. *Surf. Sci. Spectra* **1994**, *3* (3), 255–262. DOI: 10.1116/1.1247754.
(27) Lawson, K.; Wallbridge, S. P.; Catling, A. E.; Kirk, C. A.; Dann, S. E. Determination of Layered Nickel Hydroxide Phases in Materials Disordered by Stacking Faults and Interstratification. *J. Mater. Chem. A* **2023**, *11* (2), 789–799. DOI: 10.1039/D2TA07655A.
(28) Biesinger, M. C.; Lau, L. W. M.; Gerson, A. R.; Smart, R. S. C. The Role of the Auger Parameter in XPS Studies of Nickel Metal, Halides and Oxides. *PCCP* **2012**, *14* (7), 2434–2442. DOI: 10.1039/c2cp22419d. Published Online: Jan. 16, 2012.
(29) van der Heijden, O.; Park, S.; Eggebeen, J. J. J.; Koper, M. T. M. Non-Kinetic Effects Convolute Activity and Tafel Analysis for the Alkaline Oxygen Evolution Reaction on NiFeOOH Electrocatalysts. *Angew. Chem. Int. Ed.* **2023**, *62* (7), e202216477. DOI: 10.1002/anie.202216477.
(30) Khadke, P.; Tichter, T.; Boettcher, T.; Muench, F.; Ensinger, W.; Roth, C. A Simple and Effective Method for the Accurate Extraction of Kinetic Parameters Using Differential Tafel Plots. *Sci. Rep.* **2021**, *11* (1), 8974. DOI: 10.1038/s41598-021-87951-z.
(31) Hiege, F.; Sicking, L. M.; Kanokkanchana, K.; Cignoni, P.; Dudarev, V.; Ludwig, A.; Tschulik, K. The Crucial Role of Rotation Speed on the Determination of Tafel Slopes of Electrocatalysts in Rotating Disk Electrode Experiments. *ACS Electrochem.* **2025.** DOI: 10.1021/acselectrochem.5c00210.
(32) Bernt, M.; Hartig-Weiß, A.; Tovini, M. F.; El-Sayed, H. A.; Schramm, C.; Schröter, J.; Gebauer, C.; Gasteiger, H. A. Current Challenges in Catalyst Development for PEM Water Electrolyzers. *Chem. Ing. Tech.* **2020**, *92* (1-2), 31–39. DOI: 10.1002/cite.201900101.
(33) Haverkort, J. W.; Rajaei, H. Voltage Losses in Zero-Gap Alkaline Water Electrolysis. *J. Power Sources* **2021**, *497*, 229864. DOI: 10.1016/j.jpowsour.2021.229864.
(34) Yao, Y.; Zhao, G.; Guo, X.; Xiong, P.; Xu, Z.; Zhang, L.; Chen, C.; Xu, C.; Wu, T.-S.; Soo, Y.-L.; Cui, Z.; Li, M. M.-J.; Zhu, Y. Facet-Dependent Surface Restructuring on Nickel (Oxy)hydroxides: A Self-Activation Process for Enhanced Oxygen Evolution Reaction. *J. Am. Chem. Soc.* **2024**, *146* (22), 15219–15229. DOI: 10.1021/jacs.4c02292.
(35) Gallenberger, J.; Moreno Fernández, H.; Alkemper, A.; Li, M.; Tian, C.; Kaiser, B.; Hofmann, J. P. Stability and Decomposition Pathways of the NiOOH OER Active Phase of NiO_x Electrocatalysts at Open Circuit Potential Traced By *ex situ* and *in situ S*pectroscopies. *Catal. Sci. Technol.* **2023**, *13* (16), 4693–4700. DOI: 10.1039/D3CY00674C.
(36) Ali-Löytty, H.; Louie, M. W.; Singh, M. R.; Li, L.; Sanchez Casalongue, H. G.; Ogasawara, H.; Crumlin, E. J.; Liu, Z.; Bell, A. T.; Nilsson, A.; Friebel, D. Ambient-Pressure XPS Study of a Ni–Fe Electrocatalyst for the Oxygen Evolution Reaction. *J. Phys. Chem. C* **2016**, *120* (4), 2247–2253. DOI: 10.1021/acs.jpcc.5b10931.
(37) *Proceedings of the Symposium on Lithium Batteries*:*Presented at the Lithium Battery Symposium of the 194th Electrochemical Society Fall Meeting (November 1 - 6, 1998) held in Boston, Massachusetts,* Electrochemical Society; Proceedings volume / Electrochemical Society, 98-16; Electrochemical Society, 1999.

(38) Farley, N.; Gurman, S.; Hillman, A. Dynamic EXAFS Study of Discharging Nickel Hydroxide Electrode with Non-Integer Ni Valency. *Electrochim. Acta* **2001**, *46* (20-21), 3119–3127. DOI: 10.1016/S0013-4686(01)00603-X.
(39) Risch, M.; Klingan, K.; Heidkamp, J.; Ehrenberg, D.; Chernev, P.; Zaharieva, I.; Dau, H. Nickel-Oxido Structure of a Water-Oxidizing Catalyst Film. *Chem. Commun. (Cambridge, U.K.)* **2011**, *47* (43), 11912–11914. DOI: 10.1039/C1CC15072C.
(40) N. Pronkin, S.; Yu. Shokina, N.; Pham-Huu, C. Redox Transitions in Pseudocapacitor Materials: Criteria and Ruling Factors. In *Redox Chemistry - From Molecules to Energy Storage*; Fontaine, O., Ed.; IntechOpen, 2022. DOI: 10.5772/intechopen.104084.
(41) Lasia, A. *Electrochemical Impedance Spectroscopy and its Applications*; Springer New York, 2014. DOI: 10.1007/978-1-4614-8933-7.
(42) Dionigi, F.; Strasser, P. NiFe-Based (Oxy)hydroxide Catalysts for Oxygen Evolution Reaction in Non-Acidic Electrolytes. *Adv. Energy Mater.* **2016**, *6* (23). DOI: 10.1002/aenm.201600621.
(43) Brug, G. J.; van den Eeden, A.; Sluyters-Rehbach, M.; Sluyters, J. H. The Analysis of Electrode Impedances Complicated by the Presence of a Constant Phase Element. *J. Electroanal. Chem. Interf. Electrochem.* **1984**, *176* (1-2), 275–295. DOI: 10.1016/S0022-0728(84)80324-1.
(44) Lasia, A. The Origin of the Constant Phase Element. *J. Phys. Chem. Lett.* **2022**, *13* (2), 580–589. DOI: 10.1021/acs.jpclett.1c03782.
(45) Zhao, B.; Liu, J.; Xu, C.; Feng, R.; Sui, P.; Wang, L.; Zhang, J.; Luo, J.-L.; Fu, X.-Z. Hollow NiSe Nanocrystals Heterogenized with Carbon Nanotubes for Efficient Electrocatalytic Methanol Upgrading to Boost Hydrogen Co-Production. *Adv. Funct. Mater.* **2021**, *31* (8). DOI: 10.1002/adfm.202008812.
(46) Khaladkar, S.; Gund, G.; Maurya, O.; Sinha, B.; Salame, P.; Dubal, D.; Deshmukh, R.; Kalekar, A. Interface Engineering of Nickel Selenide and Graphene Nanocomposite for Hybrid Supercapacitor. *Adv. Energy Sustain. Res.* **2023**, *4* (7). DOI: 10.1002/aesr.202300013.
(47) Louie, M. W.; Bell, A. T. An Investigation of Thin-Film Ni-Fe Oxide Catalysts for the Electrochemical Evolution of Oxygen. *J. Am. Chem. Soc.* **2013**, *135* (33), 12329–12337. DOI: 10.1021/ja405351s.
(48) Anderson, A.; Sanders, A.; Smith, W. Raman Spectra of Selenium Dioxide at Low Temperatures. *J. Raman Spectrosc.* **2000**, *31* (5), 403–406. DOI: 10.1002/1097-4555(200005)31:5<403:AID-JRS529>3.0.CO;2-K.
(49) Wood, K. V.; Grange, A. H.; Taylor, J. W. Mass discrimination effects in a quadrupole mass spectrometer. *Anal. Chem.* **1978**, *50* (12), 1652–1654. DOI: 10.1021/ac50034a021.
(50) Bondue, C. J.; He, B.; Hosseini, P.; Boitel-Aullen, G.; Li, T.; Tschulik, K. Evaluating Differential Electrochemical Mass Spectrometry as a Tool to Probe the Lattice Oxygen Exchange Mechanism of Oxygen Evolution at Transition Metal Oxide Catalysts. *ChemCatChem* **2025.** DOI: 10.1002/cctc.202501346.
